# Concealed truth: Modeling reveals unique Quaternary distribution dynamics and refugia of four related endemic keystone *Abies* taxa on the Tibetan Plateau

**DOI:** 10.1002/ece3.5866

**Published:** 2019-12-04

**Authors:** Qinli Xiong, Marwa Waseem A. Halmy, Mohammed A. Dakhil, Bikram Pandey, Fengying Zhang, Lin Zhang, Kaiwen Pan, Ting Li, Xiaoming Sun, Xiaogang Wu, Yang Xiao

**Affiliations:** ^1^ CAS Key Laboratory of Mountain Ecological Restoration and Bioresource Utilization & Ecological Restoration Biodiversity Conservation Key Laboratory of Sichuan Province Chengdu Institute of Biology Chinese Academy of Sciences Chengdu China; ^2^ State Key Laboratory of Urban and Regional Ecology Research Center for Eco‐Environmental Sciences Chinese Academy of Sciences Beijing China; ^3^ Department of Environmental Sciences Faculty of Science Alexandria University Alexandria Egypt; ^4^ University of Chinese Academy of Sciences Beijing China; ^5^ Botany and Microbiology Department Faculty of Science Helwan University Cairo Egypt; ^6^ College of Biology and Environmental Sciences Jishou University Jishou China

**Keywords:** *Abies* forest, ecological niche models, fossils, phylogeography, Quaternary refugia

## Abstract

Understanding the factors driving the Quaternary distribution of *Abies* in the Tibetan Plateau (TP) is crucial for biodiversity conservation and for predicting future anthropogenic impacts on ecosystems. Here, we collected Quaternary paleo‐, palynological, and phylogeographical records from across the TP and applied ecological niche models (ENMs) to obtain a profound understanding of the different adaptation strategies and distributional changes in *Abies* trees in this unique area. We identified environmental variables affecting the different historical biogeographies of four related endemic *Abies* taxa and rebuilt their distribution patterns over different time periods, starting from the late Pleistocene. In addition, modeling and phylogeographic results were used to predict suitable refugia for *Abies forrestii*, *A. forrestii* var. *georgei*, *A. fargesii* var. *faxoniana*, and *A. recurvata*. We supplemented the ENMs by investigating pollen records and diversity patterns of cpDNA for them. The overall reconstructed distributions of these *Abies* taxa were dramatically different when the late Pleistocene was compared with the present. All *Abies* taxa gradually receded from the south toward the north in the last glacial maximum (LGM). The outcomes showed two well‐differentiated distributions: *A. fargesii* var. *faxoniana* and *A. recurvata* occurred throughout the Longmen refuge, a temporary refuge for the LGM, while the other two *Abies* taxa were distributed throughout the Heqing refuge. Both the seasonality of precipitation and the mean temperature of the driest quarter played decisive roles in driving the distribution of *A. fargesii* var. *faxoniana* and *A. recurvata*, respectively; the annual temperature range was also a key variable that explained the distribution patterns of the other two *Abies* taxa. Different adaptation strategies of trees may thus explain the differing patterns of distribution over time at the TP revealed here for endemic *Abies* taxa.

## Graphical abstract

All *Abies* species gradually receded from the south toward the north in the last glacial maximum (LGM). *Abies fargesii* var. *faxoniana* and *A. recurvata* occurred throughout the Longmen refuge, which was a temporary refuge only for the LGM, while the other two *Abies* taxa were distributed throughout the Heqing refuge. Different adaptation strategies may explain the different patterns of distribution of the four endemic *Abies* taxa.

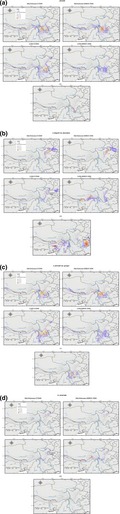

## 1 INTRODUCTION

**Figure 1:**
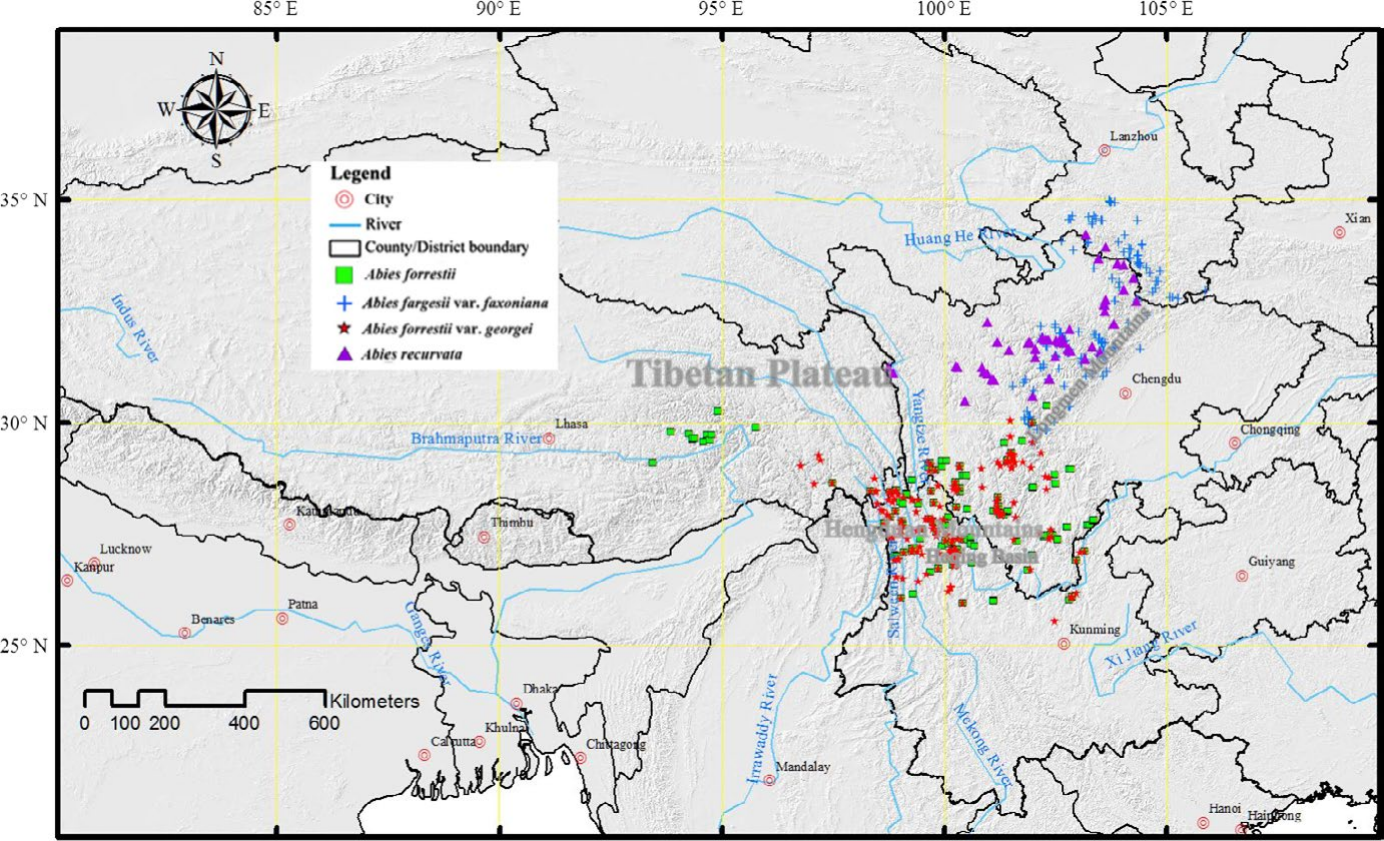
Current distribution localities of the four endemic *Abies* across the Tibetan Plateau

The Tibetan Plateau (TP) is one of the world's major hotspots of plant species diversity, harboring 12,000 species of vascular plant, including 3,673 endemic species (Wen, Zhang, Nie, Zhong, & Sun, 2014; Yu et al., 2019; Zhang et al., 2016). From a geographical standpoint, the TP is unique (Favre et al., 2015; Liu et al., 2016): Over time, great fluctuations in temperature (Liu et al., 2016; Shen et al., 2016) and precipitation (Liu et al., 2016; Shen, Piao, Cong, Zhang, & Jassens, 2015) have occurred, in addition to the effects of elevation on generating different climatic conditions (Favre et al., 2015). These factors, along with the past geological history and uplifting of the TP, together explain its high level of species richness and endemism (Favre et al., 2015; McCain & Grytnes, 2010; Xing & Ree, 2017). The unique features that characterize this region have allowed *Abies* taxa to persist and evolve over an extended period of time (Guan & Zhou, 1983; Xiang, Cao, & Zhou, 2006). The speciation events of *Abies* on the TP, and related migrations of the tree genus have become a hot research topic. Recent studies indicated the most important speciation events had occurred during the late Eocene, due to regional drying that led to a geographic isolation of drought‐sensitive taxa (Wang, Xu, et al., 2017; Xiang et al., 2015, 2006). Historical geographic distributions of four related endemic *Abies* trees (*A. fargesii* var. *faxoniana* [Rehder and E. H. Wilson] Tang S. Liu, *A. forrestii* Coltm.‐Rog., *A. forrestii* var. *georgei* [Orr] Farjon, and *A. recurvata* Mast.) constitute an unresolved biogeographic and paleo‐botanical conundrum. These four taxa vary in their morphological traits and have distinct environmental requirements (China Forest Editorial Board, 1997; Guan & Zhou, 1983). Presently, *A. fargesii* var. *faxoniana* and *A. recurvata* occur exclusively in the northeastern area of the Hengduan Mountains (Figure 1), which are located in the eastern part of the TP (Guan & Zhou, 1983). That region is characterized by a relatively warm climate with a wet summer (He, He, & Wu, 2015). By contrast, both *A. forrestii* var. *georgei* and *A. forrestii* are distributed only in TP's southeastern part (Figure 1), specifically in the southern portion of the Hengduan Mountains range (Fang, Wang, & Tang, 2011), featuring a cold and extended sunny climate with a dry winter (China Forest Editorial Board, 1997; He et al., 2015). The current distribution areas of these *Abies* taxa presumably arose from species adaptations, as the species have shifted their ranges over time (Liu, Fang, & Piao, 2002; WWF, 2017).

These four related taxa of *Abies* in the TP face serious threats due to habitat destruction and climate change (Xiang & Rushforth, 2013a, 2013b). Rising temperature and extreme precipitation have been highlighted as major factors driving the population dynamics of *Abies* taxa (He et al., 2015; Wang, Xu, et al., 2017). Further, as explained by Ordonez and Svenning (2016), historic climate data have proved instrumental for describing past events and explaining the current distribution of species. Paleoecology can provide further insight for understanding the long‐term influence of environmental changes on *Abies* taxa and their ecosystems (Tinner & Valsecchi, 2013). Also, accurate responses of plant distributions to historical climate change could serve as good predictors of ongoing climate change (Waltari et al., 2007; Willis & McElwain, 2002). Both Asian Monsoon and uplifting of the TP during the Eocene which created a unique climatic conditions have influenced the distribution and diversification of *Abies* in this area (Cao, Herzschuh, Ni, Zhao, & Böhmer, 2015; Xiang, Cao, & Zhou, 2007). Yet, studies of the driving factors or dynamics of spatial distribution patterns of *Abies* taxa and their response to habitat changes at the TP remain surprisingly limited (Xiang et al., 2006). Knowing which factors drove historical changes in *Abies* distributions might help enhance mitigation strategies for its species and contribute to the conservation of the Tibetan forests in the face of projected climate change scenarios. Ecological niche models (ENMs) can offer perspectives for answering inquiries concerning species' historic and future potential distributions (García‐Callejas & Araújo, 2016; Shrestha & Bawa, 2014). Not surprisingly then, ENMs are widely used for species distribution modeling (Dan & Seifert, 2011; Elith & Leathwick, 2009) and for assessing the degree of ecological segregation among different co‐occurring taxa (Yi, Cheng, Wieprecht, & Tang, 2014). By applying ENMs to paleo‐climate data, we can elucidate the potential past distributions of species (Du, Hou, Wang, Mao, & Hampe, 2017).

Based on pollen fossil records supported by other biological evidence, the climatic oscillations during the Quaternary period significantly influenced biome shifts at the TP (Zhang, Fengquan, & Jianmin, 2000). Evidence from its fossil records revealed the TP served as a vital refugium for numerous plant species that survived the Quaternary glaciations, including those of *Abies* (Wang & Liu, 1994). The southeast region of the TP provided refugia for the northerly biome during the Quaternary glaciation while it gradually retreated from north to south (Du et al., 2017). Identification of Quaternary refugia is based on various forms of historical biogeographic evidence, particularly those derived from paleo‐ecological studies. Such evidentiary studies helped to identify glacial refugia in the TP over the most critical periods of the Pleistocene for exemplary taxa including *Ginkgo biloba*, *Pedicularis longiflora*, *Primula secundiflora*, evergreen oaks (*Quercus aquifolioides*), and *Primula sikkimensis* (Du et al., 2017; Gong, Chen, Dobes, Fu, & Koch, 2008; Wang, Gong, Hu, & Hao, 2008; Yang, Li, Ding, & Wang, 2008). Knowledge of Quaternary refuge distributions of species long ago (Cao et al., 2015), as well as remaining refuge distributions under current climate change conditions (Tinner & Valsecchi, 2013), can inform the future protection of *Abies* trees against climate change. Several studies have addressed the biogeographic history of *Abies* species in the TP (e.g., Peng et al., 2015; Shao, Zhang, Phan, & Xiang, 2017; Xiang et al., 2015), but a thorough understanding of it still eludes us. This may be due, in part, to the lack of using integrative approaches that incorporate both palynological and phylogeographical data. The inherent biases and complexities of the palynological approach—its limitation for providing *Abies* taxonomic identifications to the species level, dearth of taxonomic precision, inability to account for underrepresented taxa in fossil records, and underestimation of migration distances and shifts in some *Abies* species distribution—have collectively hindered its ability to infer the location and timing of refugia. This has added to the difficulty in defining the spatial and temporal range of distribution of various species in the past.

This study applied ENMs to integrated paleo‐climatic data, fossil pollen records, and previous phylogeographic data of *Abies* taxa for modeling and describing the current and Quaternary distributions of four endemic *Abies* taxa. Through the use of this integrative approach, we sought to provide a better understanding of how climate fluctuations have impacted the distribution dynamics of *Abies* taxa in Hengduan Mountains biodiversity hotspot.

Ecological niche models, *Abies* paleo‐records, and phylogeography each presumes certain stability to some extent in ecological niche dimensions. Nonetheless, the integration of these employed approaches may offer better insight while also improving the accuracy of ENM applications intended to predict the potential Quaternary refugia of the study area. We hypothesized that the different adaptation strategies driven by environmental factors resulted in differing distribution patterns for the four endemic *Abies* in this area in the late Pleistocene. A better understanding of divergent adaptation strategies of *Abies* species may thus provide an effective instrument to robustly identify those vulnerable areas for which proactive conservation measures are needed in the high‐altitude region of the TP.

## 2 METHODS

### 2.1 Study area and species

The TP is surrounded by the Himalayan Mountains to the south, the Kunlun and the Qilian Mountains to the north, the Pakistan Karakorum Mountain range stretches along its west side, while the mountains of Hengduan border the east side of the plateau (Zhang, Li, & Zheng, 2002; Figure 1). Subalpine coniferous forest composed of *Abies* and *Picea* is the dominant forest type mainly covering the southeastern region of the TP (Li, 1999), particularly in the Hengduan Mountains ranges. *Abies* species are widespread in this area, dominating the subalpine forests at elevations of 2,500–4,100 m a.s.l. (Li, 1993; Mackinnon et al., 1996).

*Abies fargesii* var. *faxoniana* is the representative tree taxon of dark coniferous forests distributed in the northern part of the Hengduan Mountains area, east of the TP (Guan & Zhou, 1983), bordered to the east by the Longmen Mountains, to the west by Jinchuan County, and to the north by the Balang Mountains and Tao River Basin, with an elevation range of 2,500–3,000 m a.s.l. (Xiang et al., 2006). *Abies recurvata* is an endemic species in the TP; it grows along the basin of Bailongjiang River south of Gansu Province and in the north and northwest of Sichuan Province, across a broad elevation of 2,300–3,600 m a.s.l. Both *A. forrestii* and *A. forrestii* var.* georgei* are the main taxa of the forest south of Hengduan Mountains, at the southeastern side of the TP (China Forest Editorial Board, 1997; Fang et al., 2011). *Abies forrestii* grows mainly in southwest Sichuan Province and the northern part of Yunnan Province, eastern Tibetan, as well as in Kachin State in Myanmar and Bhutan (Fang et al., 2011). *Abies forrestii* var.* georgei* is distributed mainly in southwestern Sichuan and northwestern Yunnan and occurs from the eastern section of the Brahmaputra River in Tibet to downstream of the Yalong River located in Sichuan Province (China Forest Editorial Board, 1997). *Abies forrestii* var. *georgei* is found at 3,500–4,500 m a.s.l., while *A. fargesii* var.* faxoniana* grows at a lower elevation, from 2,500 to 3,000 m a.s.l. (China Forest Editorial Board, 1997). Recently, *A. recurvata* and *A. fargesii* var. *faxoniana* have been accorded conservation status, being judged vulnerable taxa (Xiang & Rushforth, 2013a, 2013b), while the status of *A. forrestii* var. *georgei* is currently of least concern (Zhang, Katsuki, & Rushforth, 2013b) and that of *A. forrestii* (syn. *A. forrestii* var. *forrestii*) is considered as near‐threatened (Zhang, Katsuki, & Rushforth, 2013a). Studies have suggested the taxonomic boundaries are well‐established at the species and intraspecific levels of the *Abies* genus (e.g., Aguirre‐Planter et al., 2012; Cinget, Lafontaine, Gérardi, & Bousquet, 2015; Shao & Xiang, 2015; Shao et al., 2017; Wang, Abbott, Ingvarsson, & Liu, 2014); however, their intrageneric classification is an active area of research (Eckenwalder, 2009; Farjon, 2010). These four taxa selected for study vary in their morphological traits and have dissimilar environmental requirements, according to information from other studies (e.g., China Forest Editorial Board, 1997; Guan & Zhou, 1983; Shao et al., 2017).

### 2.2 Taxa occurrence data

Occurrence records of the four *Abies* taxa were obtained from a field survey conducted in September 2015 and June 2016, and from collected data archived in numerous databases, namely. the GBIF (Global Biodiversity Information Facility, http://www.gbif.org), the CVH (Chinese Virtual Herbarium, http://www.cvh.org.cn/), the NSII (National Specimen Information Infrastructure, http://www.nsii.org.cn/), and other related sources (see all references in the “Additional Reference List” in Appendix S1). Redundant points were removed to ensure a minimum of 1,000 m was maintained as the distance separating data records, to minimize the influence of spatial autocorrelation on the modeling results. In total, 212, 69, 184, and 220 valid effective occurrence records for these four taxa were collected (Figure 1).

### 2.3 Environmental data and selection of variables

To represent the environmental conditions in different climatic periods, 19 bioclimatic predictors obtained from the WorldClim database (Hijmans, Cameron, Parra, Jones, & Jarvis, 2005) were used, in addition to two indirect gradients predictors (slope and aspect). Downscaled data from two general circulation models (GCMs), namely the CCSM4 and MIROC‐ESM (Brady, Otto‐Bliesner, Kay, & Rosenbloom, 2013; Raes et al., 2014; Sueyoshi et al., 2013), were used to obtain climatic data for the LGM (21 kyr BP) and the mid‐Holocene (6 kyr BP). The LIG data (120–140 kyr BP), with a spatial resolution of 30 arc seconds, were obtained following Otto‐Bliesner, Marshall, Overpeck, Miller, and Hu (2006) from the WorldClim 1.4 (http://www.worldclim.org; Hijmans et al., 2005). The last two variables corresponded to indirect abiotic gradients: The slope and aspect derived from the digital terrain model (DTM) were extracted from the Shuttle Radar Topography Mission (SRTM) (http://srtm.csi.cgiar.org/) at 30 arc‐second resolution. Principal component analysis (PCA) was performed on standardized and centered data of the 19 current bioclimatic predictors and two indirect gradients (Raes et al., 2014). The first three components (PCs) accounted for 98.48% of the variance in the dataset employed in the analysis (Figure S1).

Multicollinearity can cause the mode to overfit the data used. Therefore, the Pearson correlations were tested for all pairwise combinations of the 19 climate datasets (Raes et al., 2014). The SDMtoolbox (Brown, 2014; Brown, Bennett, & French, 2017) was used within the framework of ArcGIS 10.0 (ESRI, Redlands, CA, USA) to conduct a correlation analysis among the 19 climate datasets' variables' grids that represented the study area. Only uncorrelated variables with a spatial correlation value below 0.75 were retained (Table S1; Raes et al., 2014). To calibrate the ENMs for each species, nine selected predictive variables were used: mean diurnal range (MDR), temperature annual range (TAR), mean temperatures of driest (TDQ) and wettest (TWQ) quarter, precipitation of wettest (Pmax), driest month (Pmin), precipitation of seasonality (PS), and warmest (PHQ) and coldest (PCQ) quarter, along with another two predictors: slope and aspect.

### 2.4 Modeling algorithm: Maxent

The maximum entropy (Maxent) modeling technique (Phillips, Anderson, & Schapire, 2006) was applied to the above data, it being one of the most commonly used and accurate ways to predict species' distributions and habitat suitability (Dan & Seifert, 2011; Elith & Leathwick, 2009). The models for each taxon were first calibrated for the current distributions relative to the current climate; the most accurate models were then applied to the historical data (LIG, LGM, mid‐Holocene) to get predictions for those past periods. The occurrence records datasets were randomly split (75%–25%), where the 75% portion for each taxon was used to calibrate the algorithm, and the remaining 25% used for evaluating the produced ENMs (Bueno et al., 2017). The version of Maxent used was obtained from http://biodiversityinformatics.amnh.org/open_source/maxent/; for each taxon, *n* = 200 replicate runs were undertaken in Maxent 3.4.0.

### 2.5 Model calibration and evaluation

The ENM models developed to determine the potential distribution of *Abies* taxa had a logistic output format, which quantifies habitat suitability as a continuous probability value ranging from 0 to 1 (Phillips & Dudik, 2008). The potential distribution curves were estimated for each predictor, and the percentage corresponding to the relative contribution of each one to the habitat suitability models were assessed using Maxent's built‐in jackknife test. To evaluate the models and assess their performance, the receiver operating characteristic (ROC) curve was plotted, for which the area under the curve (AUC) was estimated at the possible thresholds (Raes et al., 2014). The ROC curve is generated by plotting the sensitivity versus 1–specificity for the entire set of runs. The AUC provides a widely used metric of model accuracy (Fielding & Bell, 1997). Models attaining an AUC value > 0.7 are deemed of acceptable performance (Swets, 1988).

The response curves for the predictors used in building the models were then generated. Through them, we could gain insight into the quantitative relationships among investigated environmental predictors and the logistic probability of species existence. More specifically, such response curves mirror the favorable ecological niche of the species (Dan & Seifert, 2011).

To minimize the biases to the objective methods that integrated sensitivity with specificity, the maximum training sensitivity plus specificity (Tmax) and the minimum training presence (Tmin) logistic thresholds (Vedelsørensen, Tovaranonte, Bøcher, Balslev, & Barfod, 2013) were each retrieved from Maxent. This was done in addition to using the normal rank grading method (Raes et al., 2014).

### 2.6 Multivariate analysis of bioenvironmental data

Redundancy analysis (RDA) was conducted for a relative assessment of the different environmental requirements of *A. forrestii*, *A. fargesii* var. *faxoniana*, *A. forrestii* var. *georgei*, and *A. recurvata*. The RDA was performed on the set of environmental variables, based on the their relative contribution percentage to the species habitat suitability models produced by Maxent (mean diurnal range, temperature annual range, mean temperature of wettest/driest quarter, precipitation of seasonality, precipitation of warmest/coldest quarter, and slope), ranked according to their quantitative importance by a forward selection process. Those variables were used to analyze the effect of climate and topography on the pattern of four *Abies* taxa distributions. The R software environment and its “vegan” package (R Development Team, 2008) were used to carry out this statistical analysis and produce its graphics.

### 2.7 Paleoclimate, fossil, and phylogeographic records

Climate variables representing the LGM and the mid‐Holocene paleoclimates were obtained from CCSM4 and MIROC‐ESM general circulation models (GCMs) for inclusion in the analysis. Furthermore, data from the last interglacial period (Otto‐Bliesner et al., 2006) were obtained from sporopollen, carbon isotope measurements, calcium carbonate, and ice cores for the area under study (Table S2). According to our review, the fossil and pollen sequences dated from or around the 6 kyr BP, 21 kyr BP, and 120–140 kyr BP periods in the TP (Table 1). Indications of when the assessed pollen sequence chronology overlapped with the LIG, LGM, or mid‐Holocene periods are given in Table 1. Published cpDNA data were used to derive diversity patterns for use in the phylogeographical validation of the model hypotheses (Peng et al., 2012; Zhan, 2011). Since phylogeographical investigations of *Abies* taxa are limited at the TP, the evaluation of published cpDNA trnL‐F, trnS‐G, and ndhkC data was possible for only two *Abies* taxa, namely *A. fargesii* var. *faxoniana* (Zhan, 2011) and *A. recurvata* (Peng et al., 2012) (Table S3; Appendix S1). Additional details on the genetic diversity index and expansion calculations are given as Appendix S1.

**Table 1:** Comparison between published pollen records and model predictions for forest occurrence at LIG (120–140 kyr BP), LGM (21 kyr BP), and mid‐Holocene (6 kyr BP) as shown in the stability area map of Figures 3 and S3 (unless otherwise specified)

Code	Site name	Latitude longitude	Estimated chronology (kyr BP)	Site type	Reference^a^	Model prediction	Match between model by CCSM (MIROC) and data
1	Heqing Basin, Yunnan Province	25.850N 100.483E	349–128	*Abies* spp.	Xiao et al. (2006)	Inside area of stability at LIG	Yes, model by Otto‐Bliesner et al. (2006)
2	Kunming Basin, Yunnan Province	25.250N 102.517E	201–120	*Tsuga chinensis*, Chenopodiaceae, *Quercus* spp., Gramineae	Xu et al. (2009)	Outside area of stability at LIG	Yes, model by Otto‐Bliesner et al. (2006)
2–1	Diancang Mountain, Dali, Yunnan Province	25.646N 100.109E	122–118	*Abies* spp.	Kuang et al. (2002)	Inside area of stability at LIG	Yes, model by Otto‐Bliesner et al. (2006)
2–2	Beihai Lake, Tengchong, Yunnan Province	25.250N 102.517E	150–126	*Abies* spp.	Bao (2010)	Inside area of stability at LIG	Yes, model by Otto‐Bliesner et al. (2006)
2–3	Zhang'an, Pingliang, Gansu Province	35.568N 105.659E	200–140	*Corylus* spp., *Nitraria* spp., *Ephedra* spp., Chenopodiaceae, Compositae, *Pinus* spp., *Quercus* spp., Chenopodiaceae, Gramineae	Liu and Su (1994)	Outside area of stability at LIG	Yes, model by Otto‐Bliesner et al. (2006)
2–4	Xiyan Mountain, Huining, Gansu Province	35.658N 105.007E	200–140	Tamaricaceae, *Pinus* spp., *Betula* spp., *Quercus* spp., *Corylus* spp.	Liu (1992)	Outside area of stability at LIG	Yes, model by Otto‐Bliesner et al. (2006)
2–5	Luochuan, Gansu Province	35.717N 109.517E	127–123	Betulaceae, Chenopodiaceae, Urticaceae, Chenopodiaceae, Compositae, Rosaceae, Urticaceae, Compositae, Humulus, Ranunculaceae, Cruciferae	Li (2008)	Outside area of stability at LIG	Yes, model by Otto‐Bliesner et al. (2006)
2–6	Naqu, Tibet	31.467N 91.508E	116–37	*Selaginella* spp., *Lycopodium* spp., *Osmunda* spp., *Pleuromanes* spp., Cyathea, *Asplenium* spp., Filicinae, *Concentricystes* spp., *Carya* spp., Aquifoliaceae, *Pinus* spp., *Quercus* spp.	Zhao et al. (2005)	Outside area of stability at LIG	Yes, model by Otto‐Bliesner et al. (2006)
2–7	Qi Mountain, Shaanxi Province	34.456N 107.623E	128–10	*Quercus* spp., *Ailanthus* spp., *Carpinus* spp., *Ulmus* spp., *Corylus* spp., *Juglans* spp., *Pterocarya* spp., *Platycarya* spp., *Artemisia* spp.	Zhao and Huang (1999)	Outside area of stability at LIG	Yes, model by Otto‐Bliesner et al. (2006)
2–8	Wugong, Shaanxi Province	34.232N 109.367E	110–100	*Artemisia* spp., *Pinus* spp., *Betula* spp., *Quercus* spp., *Alnus* spp., *Corylus* spp., *Concentricystes* spp., *Lycopodium* spp., *Juglans* spp., *Morus* spp., *Acer* spp., *Pterocarya* spp., *Fagus* spp., *Selaginella* spp., *Polygonum* spp.	Liu (1989)	Outside area of stability at LIG	Yes, model by Otto‐Bliesner et al., 2006
2–9	Zari, Tibet	30.928N 85.626E	120–119	*Pinus* spp., Chenopodiaceae, Gramineae, Cyperaceae	Yu (2008)	Outside area of stability at LIG	Yes, model by Otto‐Bliesner et al. (2006)
3	Luoji Mountain, Xichang, Sichuan Province	27.756N 102.328E	22	*Abies* spp.	Jiang et al. (2000)	Inside area of stability at LGM	Yes (Yes)
4	Heqing Basin, Yunnan Province	25.850N 100.483E	28.87–16.98	*Abies* spp.	Xiao et al. (2006)	Inside area of stability at LGM	Yes (Yes)
5	Hongya County, Sichuan Province	29.583N 102.433E	22.5–20.5	*Abies* spp.	Shi (2012)	Inside area of stability at LGM	Yes (Yes)
6	Butuo County, Southwestern Sichuan Province	27.717N 102.867E	22.0–11.8	*Abies* spp.	Liu et al., (2003)	Inside area of stability at LGM	Yes (Yes)
7	Northwest of Daliang Mountain, Sichuan	28.100N 103.500E	30–14	*Abies* spp.	Liu et al., (2003)	Inside area of stability at LGM	Yes (Yes)
8	Yanbian County, Sichuan Province	26.734N 101.653E	25.66	*Abies* spp.	Ye et al. (1986)	Inside area of stability at LGM	Yes (Yes)
9	Lugu Town, Mianning County, Sichuan Province	28.283N 102.183E	22	*Abies* spp.	Cheng (2010)	Inside area of stability at LGM	Yes (Yes)
10	Xiaohaizi, Leibo, Sichuan Province	28.412N103.781E	16	*Abies* spp.	Liu et al. (2004)	Inside area of stability at LGM	Yes (Yes for Tmin area)
11	Wuben Village, Panzhihua City, Sichuan Province	26.689N 101.725E	20.455	*Abies* spp.	Ye et al. (1986)	Inside area of stability at LGM	Yes (Yes)
12	Jiantang County, Shangri‐la County, Yunnan Province	27.817N 99.700E	16.552–16.952	*Cornopteris acutiloba, Blechnidium melanopus, Gymnotheca involucrata, Acorus calamus, Selaginella doedenleinii, Peperomia duclouxii, Girardihia palmata*	Shi (2012)	Outside area of stability at LGM	Yes for Tmax area (No)
13	Jiantang County, Shangri‐la County, Yunnan Province	27.817N 99.700E	24.827–25.458	*Gymnotheca involucrata, Acorus calamus, Isoetes japonica, Tamarix chinensis, Cedrus deodara, Fokienia hodginsis, Jungermannia rotundata, Barbula rigidula, Helwingia japonica*	Shi (2012)	Outside area of stability at LGM	Yes for Tmax area (No)
14	Daganba, Guizhou Province	26.500N 105.700E	23	*Pinus* spp., *Fagus* spp., *Quercus* spp., Cyperaceae, Gramineae	Han and Yu (1988)	Outside area of stability at LGM	Yes (Yes for Tmax area)
15	Shillong Village, Caohai, Bijie City, Guizhou Province	26.800N 104.167E	22	*Pinus* spp., *Salix* spp., Cyperaceae, Gramineae, *Lycopodium japonicum*	Chen (1987)	Outside area of stability at LGM	No (No)
16	Kunming Basin, Yunnan Province	25.250N 102.517E	30–20	*Pinus* spp., *Artemisia* spp., Chenopodiaceae, *Myriophyllum verticillatum*	Xu et al. (2009)	Outside area of stability at LGM	No (No)
17	ZhaojiaYuanzi, Caohai, Bijie City, Guizhou Province	26.888N 104.221E	21.0–19.1	*Cyclobalanopsis* spp., *Fagus* spp., *Quercus* spp., Ericaceae, Betulaceae, *Pinus* spp., *Tsuga*	Chen et al. (1993)	Outside area of stability at LGM	No (No)
18	Hongya County, Sichuan Province	29.583N 103.267E	22.5–20.5	Deciduous broad‐leaved forest (*Hicriopteris glauca*, *Polypodiodes chinensis*, *Alnus cremastogyne*, *Alnus ferdinandicoburgii*, *Acorus calamus*, *Saururus chinensis*, *Cyclobalanopsis glauca*, *Pinus* spp., *Tsuga dumosa*, *Morus alba*)	Shi (2012)	Outside area of stability at LGM	Yes (Yes)
2–10	Peigucuo Lake, Tibet	28.001N 85.001E	31–15	*Abies* spp.	Yu (2008)	Inside area of stability at LGM	Yes (Yes)
2–11	Lantian, Shaanxi Province	34.154N 109.331E	85–10	*Abies* spp.	Li (2005)	Inside area of stability at LGM	Yes for Tmin area (No)
2–12	Diexi, Sichuan Province	32.041N 103.679E	30.83–16.902	*Abies* spp.	Wang and Wang (2013)	Inside area of stability at LGM	Yes (Yes)
2–13	Lugu Lake, Yunnan Province	27.667N 100.800E	22.77–21.86	*Abies* spp.	Liao (2017)	Inside area of stability at LGM	Yes (Yes)
2–14	Songpinggou, Sichuan Province	32.050N 103.670E	20.18–19	*Abies* spp.	Mao (2011)	Inside area of stability at LGM	Yes (Yes)
2–15	Beihai Lake, Tengchong, Yunnan Province	25.830N 98.750E	32–15	*Abies* spp.	Bao (2010)	Inside area of stability at LGM	Yes (Yes)
2–16	Tushi Lake, Tibet	28.810N 85.586E	20	Cyperaceae, *Artemisia* spp., fern	Yu (2008)	Outside area of stability at LGM	Yes (Yes)
2–17	Dayanggou, Gansu Province	35.397N 103.913E	35–17	*Pinus* spp., *Picea* spp., *Artemisia* spp.	Wu et al. (1985)	Outside area of stability at LGM	Yes (Yes)
2–18	Xiaowangou, Tongwei, Gansu Province	35.278N 105.446E	20.714–10	Polypodiaceae, *Pinus* spp., *Picea* spp., *Lycopodium japonicum* Thunb. ex Murray, Chenopodiaceae	Wu et al. (1985)	Outside area of stability at LGM	Yes (Yes)
2–19	Landigou, Tongwei, Gansu Province	35.201N 105.578E	23.783–15.135	Polypodiaceae, *Pinus* spp., *Picea* spp., Chenopodiaceae	Wu et al. (1985)	Outside area of stability at LGM	Yes (Yes)
2–20	Jingning, Gansu Province	35.500N 105.833E	23.4–14.6	Cupressaceae, Zygophyllaceae, Compositae, Gramineae, Chenopodiaceae, *Polygonaceae*, Ranunculaceae, Ulmaceae	Tang et al. (2007)	Outside area of stability at LGM	Yes (Yes)
2–21	Fu County, Shaanxi Province	36.000N 109.500E	73–10	*Corylus* spp., *Quercus* spp., *Ailanthus* spp., *Quercus* spp., *Ulmus* spp., *Betula* spp., *Celtis* spp., Oleaceae,* Cornus* spp., *Salix* spp., *Toxicodendron vernicifluum*, *Platycarya strobilace*, *Pinus* spp., *Picea* spp., *Tsuga* spp.	Li and Ke (2006)	Outside area of stability at LGM	Yes (Yes)
2–22	Renacuo Lake, Gaize, Tibet	32.833N 84.250E	33.4–10.6	*Pinus* spp., *Ulmus* spp., *Picea* spp., *Juglans* spp., *Betula* spp., *Cedrus* spp., *Artemisia* spp., *Humulus* spp., *Selaginella* spp., *Microlepia* spp., *Ephedra* spp., *Corylus* spp.	Li (2014)	Outside area of stability at LGM	Yes (Yes)
2–23	Milin, Tibet	29.216N 94.213E	23–18	*Pinus* spp.	Pan et al. (2013)	Outside area of stability at LGM	Yes (No)
2–24	Huangheyuan, Qinghai Province	34.700N 97.500E	28–8	Chenopodiaceae, Gramineae, Tamaricaceae	Han et al. (2011)	Outside area of stability at LGM	Yes (Yes)
2–25	Qiao County, Jingning, Gansu Province	35.500N 105.833E	44–11	*Pinus* spp., *Picea* spp., *Abies* spp., *Tsuga* spp., *Larix* spp., *Betula* spp., *Quercus* spp., *Ulmus* spp., *Artemisia* spp., *Pyrrosia* spp., *Selaginella* spp., *Selaginella sinensis*, *Hicriopteris* spp., *Microlepria* spp., *Acer* spp., *Juglans* spp., *Salix* spp.	Li et al. (2006)	Outside area of stability at LGM	Yes (Yes)
2–26	Gasikule Salt Lake, Qinghai Province	38.085N 90.938E	30–20	Moraceae, Tamaricaceae, Actinidiaceae, Scrophulariaceae, Gramineae, Chenopodiaceae, Compositae, Leguminosae, Ranunculaceae, Thalictrum, Solanaceae, Rosaceae, Berberidaceae, Convolvulaceae, Labiatae, Caryophyllaceae, Cruciferae, Cyperaceae, Polypodiaceae	Ye et al. (2013)	Outside area of stability at LGM	Yes (Yes)
2–27	Jiuquan, Gansu Province	39.720N 99.370E	16	*Pinus* spp., *Picea* spp.	Shen (2008)	Outside area of stability at LGM	Yes (Yes)
2–28	Duantouliang, Tengger Desert, Gansu Province	37.500N 104.8E	28–23	*Betula* spp., *Artemisia* spp., *Juniperus* spp., *Pinus* spp., *Quercus* spp., *Salix* spp., *Rosa* spp., Hippophae	Ma et al. (1998)	Outside area of stability at LGM	Yes (Yes)
19	Hongyuan region, Zoige Plateau, Sichuan Province	32.788N 102.527E	7.4–5.0	*Abies* spp.	Wang et al. (2006)	Inside area of stability at mid‐Holocene	Yes (Yes for Tmin area)
20	Arming River Basin, Western Sichuan Province	28.275N 102.167E	10.3–4.1	*Abies* spp.	Cheng et al. (2010)	Inside area of stability at mid‐Holocene	Yes (Yes)
21	Heqing Basin, Yunnan Province	25.850N 100.483E	6.98	*Abies* spp.	Xiao et al. (2006)	Inside area of stability at mid‐Holocene	Yes (Yes)
22	Butuo County, Southwestern Sichuan Province	27.717N 102.867E	8.6–4.0	*Abies* spp.	Liu et al. (2003)	Inside area of stability at mid‐Holocene	Yes (Yes)
23	Lugu Town, Mianning County, Sichuan Province	28.283N 102.183E	6	*Abies* spp.	Cheng (2010)	Inside area of stability at mid‐Holocene	Yes (Yes)
24	Yihai, Mianning County, Sichuan Province	28.700N 102.183E	5	*Abies* spp.	Chen (1985)	Inside area of stability at mid‐Holocene	Yes (Yes)
25	Lugu Lake Watershed, Yunnan Province	27.667N 100.800E	7–5	*Abies* spp.	Zhang et al. (2016)	Inside area of stability at mid‐Holocene	Yes (Yes)
26	Lake Rukche area, Gorkha Himal, Central Nepal	28.296N 84.776E	7.8–2.75	Humid oak forests with demanding elements dominated the vegetation cover	Schlütz and Zech (2004)	Outside area of stability at mid‐Holocene	No (No)
27	Anlong County, Sichuan Province	25.167N 105.157E	5.99	*Pteris cretica*, *Dipteris conjugata*, *Pyrrosia lingua*, *Polypodiode snipponica*, *Aleuritopteris pseudofarinosa*, *Keteleeria fortunei*, *Taxodium distichum*, *Adiantum Capillusveneris*	Mao (1991)	Outside area of stability at mid‐Holocene	Yes (Yes)
28	Fanjing Mountain, Guizhou Province	27.786N 108.560E	5.53	*Cyclobalanopsis glauca*, *Quercus* spp., *Castanopsis* spp., Betulaceae, Polypodiaceae, Athyriaceae	Chen (1989)	Outside area of stability at mid‐Holocene	Yes (Yes)
29	Shillong Village, Caohai Town, Weining County, Bijie City, Guizhou Province	26.800N 102.167E	6.0–4.8	*Picea asperata*, *Tsuga chinensis*, *Salix* spp., *Pinus* spp., *Quercus* spp., *Cyclobalanopsis glauca*, *Athyrium filixfemina*, *Aleuritopteris pseudofarinosa*, *Lycopodium japonicum*	Chen (1987)	Outside area of stability at mid‐Holocene	Yes for Tmax area (No)
30	Zoige Plateau, Sichuan Province	34.083N 102.167E	6.42–3.79	Cyperaceae, Gramineae, Asteraceae, Equisetaceae, Ranunculaceae, *Pinus* spp., Oleaceae, *Betula* spp., *Quercus* spp., *Castanopsis* spp.	Guo et al. (2012)	Outside area of stability at mid‐Holocene	Yes for Tmax area (Yes)
31	Shangri‐la County, Yunnan Province	27.817N 99.700E	5.5	*Blechnum orientale*, *Dipteris conjugate, Hicriopteris glauca*, *Gymnotheca involucrata*, *Acorus calamus*, *Mimosa pudica*, *Tamarix chinensis*, *Cupressus duclouxiana*, *Platycladus orientalis*, *Betula platyphylla*, *Ginkgo biloba*, *Rhapis excelsa*, *Vitex negundo*	Shi (2012)	Outside area of stability at mid‐Holocene	No (No)
32	Guanzhai Village, Zhijin County, Bijie City, Guizhou Province	26.769N 105.897E	6.71	*Pinus* spp., *Aleuritopteris pseudofarinosa*, *Artemisia* spp.	Zhu and Li (1994)	Outside area of stability at mid‐Holocene	Yes (Yes)
33	Waqie Town, Hongyuan County, Sichuan Province	33.150N 102.850E	8.512–5.000	Meadow	Huang et al. (2012)	Outside area of stability at mid‐Holocene	Yes for Tmax area (No)
34	Chengdu Plain, Sichuan Province	30.500N 103.000E	4	*Abies* spp.	Luo et al. (2008)	Inside area of stability at mid‐Holocene	Yes (Yes)
35	Mianning Area, Sichuan Province	29.000N 102.167E	8.8–5.0	*Abies* spp.	Dong et al. (2000)	Inside area of stability at mid‐Holocene	No (Yes)
36	Xiaohaizi, Leibo, Sichuan Province	28.412N103.781E	9.0–5.3	*Abies* spp.	Liu et al. (2004)	Inside area of stability at mid‐Holocene	Yes (Yes)
2–29	Heqing, Yunnan Province	26.573N 100.128E	6.98	*Abies* spp.	Xiao et al. (2006)	Inside area of stability at mid‐Holocene	Yes (Yes)
2–30	Heihe farm, Zoige Plateau, Sichuan Province	33.906N 102.536E	9–3	*Abies* spp.	Liu et al. (1995)	Inside area of stability at mid‐Holocene	Yes for Tmin area (Yes for Tmin area)
2–31	Zoige Plateau, Sichuan Province	33.773N 102.550E	6.46	*Abies* spp.	Cai (2008)	Inside area of stability at mid‐Holocene	Yes for Tmin area (Yes for Tmin area)
2–32	Hongyuan peatland, Sichuan Province	32.778N 102.517E	11.5–3	*Abies* spp.	Zhou et al. (2011)	Inside area of stability at mid‐Holocene	Yes (Yes for Tmin area)
2–33	Heqing, Dali County, Yunnan Province	26.564N 100.175E	6.98	*Abies* spp.	Xiao et al. (2006)	Inside area of stability at mid‐Holocene	Yes (Yes)
2–34	Dawan, Gansu Province	34.800N 105.915E	9.5–7.5	*Abies* spp.	Tang and An (2007)	Inside area of stability at mid‐Holocene	Yes (Yes)
2–35	Lantian, Shaanxi Province	34.152N 109.324E	5	*Abies* spp.	Li and Sun (2005)	Inside area of stability at mid‐Holocene	Yes for Tmin area (Yes for Tmin area)
2–36	Anlong County, Guizhou Province	25.233N 105.355E	5.99	*Pinus* spp., Gramineae, *Keteleeria* spp., *Cupressus* spp.	Mao (1991)	Outside area of stability at mid‐Holocene	Yes (Yes)
2–37	Fanjing Mountain, Guizhou Province	27.786N 108.560E	5.53–0.16	Gramineae, Polypodiaceae, Athyriaceae, Sinopteridaceae, Cyperaceae, Gramineae, Sinopteridaceae, Polypodiaceae, Athyriaceae	Chen (1989)	Outside area of stability at mid‐Holocene	Yes (Yes)
2–38	Dadiwan, Longzhong, Gansu Province	35.000N 105.915E	8.2–4.3	*Ulmus pumila* L., *Quercus* spp., Betulaceae	Wei et al. (2009)	Outside area of stability at mid‐Holocene	Yes for Tmax area (No)
2–39	Sujiawan, Gansu Province	35.539N 104.526E	8.2–4.3	*Ulmus pumila* L., *Quercus* spp., Betulaceae	Wei et al. (2009)	Outside area of stability at mid‐Holocene	Yes (Yes)
2–40	Landigou, Tongwei County, Gansu Province	35.201N 105.578E	15.135–0	*Pinus* spp., Rosaceae, Labiatae, Malvaceae	Wu et al. (1985)	Outside area of stability at mid‐Holocene	Yes for Tmax area (Yes for Tmax area)
2–41	Majiacha, Dingxi County, Gansu Province	35.563N 104.654E	7.823–7.663	*Pinus* spp., Chenopodiaceae, Ephedraceae, Compositae, Polypodiaceae	Wu et al. (1985)	Outside area of stability at mid‐Holocene	Yes (Yes)
2–42	Xishuangbanna, Yunnan Province	22.001N 100.837E	7.25–0	*Pinus* spp., *Podocarpus* spp., *Keteleeria* spp., *Dacrydium* spp., *Terminalia* spp., *Homonoia* spp., *Lithocarpus* spp., *Castanopsis* spp., *Alnus* spp., *Ilex* spp., *Quercus* spp., *Liquidambar* spp., *Fagus* spp., *Carya* spp., *Casuarina* spp., *Trema* spp., *Pterocarya* spp., *Rhoiptelea* spp., *Apodytis* spp., *Tilia* spp., *Engelhardtia* spp., *Gleditsia* spp., *Duabanga* spp., *Erythrina* spp., *Bridelia* spp., *Phyllanthus* spp., *Mallothus* spp., *Cephalanthus* spp.	Xu et al. (1998)	Outside area of stability at mid‐Holocene	Yes (Yes)
2–43	Yan'an, Shaanxi Province	36.585N 109.490E	8.13–4.37	*Pinus* spp., Gramineae, Ranunculaceae	He et al. (2000)	Outside area of stability at mid‐Holocene	Yes (Yes)
2–44	Qilian Mountain, Qinghai Province	38.202N 102.772E	7–6.3	*Picea* spp., *Sabina* spp.	Zhu et al. (2001)	Outside area of stability at mid‐Holocene	Yes (Yes)
2–45	Fanjing Mountain, Guizhou Province	27.940N 108.614E	10.0–8.1	Symplocaceae, Cyperaceae, Gramineae, Chenopodiaceae, Compositae, Caryophyllaceae, Polygonaceae, Ranunculaceae, Rosaceae, Balsaminaceae, Cruciferae, Labiatae, Geraniaceae, Typhaceae, Athyriaceae, Polypodiaceae, Sinopteridaceae, Dennstaedtiaceae, Pteridaceae, Vittariaceae	Chen et al. (1992)	Outside area of stability at mid‐Holocene	Yes (Yes)
2–46	Huining County, Gansu Province	35.859N 105.007E	5–0	*Betula* spp., *Quercus* spp., *Pinus* spp.	Liu (1992)	Outside area of stability at mid‐Holocene	Yes (Yes)
2–47	Tanggula Mountain, Tibet	34.214N 92.437E	11–4	*Pinus* spp., *Picea* spp., *Alnus* spp., *Castanea* spp., *Ulmus* spp., *Artemisia* spp., *Ephedra* spp., *Chenopodium* spp., *Dianthus* spp., *Sparganium* spp.	Wu et al. (2006)	Outside area of stability at mid‐Holocene	Yes (Yes)
2–48	Wuda, Dangxiong, Yunnan Province	30.507N 91.240E	9–4	*Humulus* spp., *Quercus* spp., *Corylus* spp., *Rhododendron* spp., *Artemisia* spp., Sensu spp., Cyperaceae, Liliaceae, Labiatae, Oleaceae	Wang et al. (1981)	Outside area of stability at mid‐Holocene	Yes (Yes)

Note

Numbers refer to localities shown in Figures S4 and S5.

a All references of this table are shown in Appendix S1.

## 3 RESULTS

### 3.1 Climate scenarios

The summarized data for 14 deposits of the LGM and the mid‐Holocene obtained from the final interpolated models (CCSM4 and MIROC‐ESM), and for one point of the LIG climate data for TP, are presented in Tables S2 and S4. Also provided in Table S2 are the discrepancies between GCMs' values through the CCSM4 and MIROC‐ESM simulations and the paleoclimates, which were obtained from calcium carbonate, sporopollen, and ice cores for the different climate scenarios applied. Annual mean temperature (Tann) and annual precipitation (Pann) had consistently higher accuracy in the CCSM4 than MIROC‐ESM simulation, but with no significant differences for the mid‐Holocene in the CCSM4 and MIROC‐ESM simulations. In general, the temperature simulations by the CCSM4 and MIROC‐ESM models for the LGM were better than those for the mid‐Holocene, while precipitation simulations were better than temperature simulations for all periods.

### 3.2 Potential distributions of *Abies* taxa in the LIG, LGM, and mid‐Holocene

**Figure 2:**
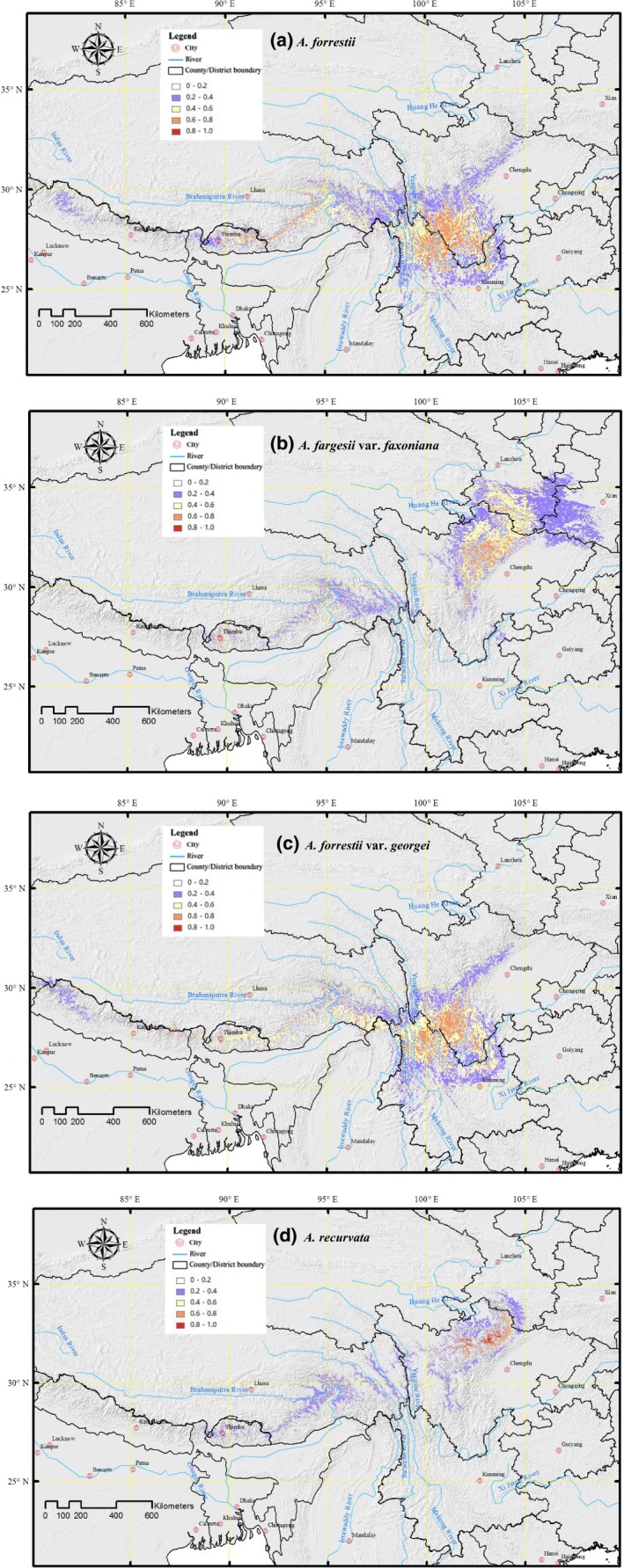
Precited potential distribution of the four *Abies* taxa across the Tibetan Plateau based on current climate (a) *A. forrestii*, (b) *A. fargesii* var. *faxoniana*, (c) *A. forrestii* var. *georgei*, and (d) *A. recurvata*

**Figure 3:**
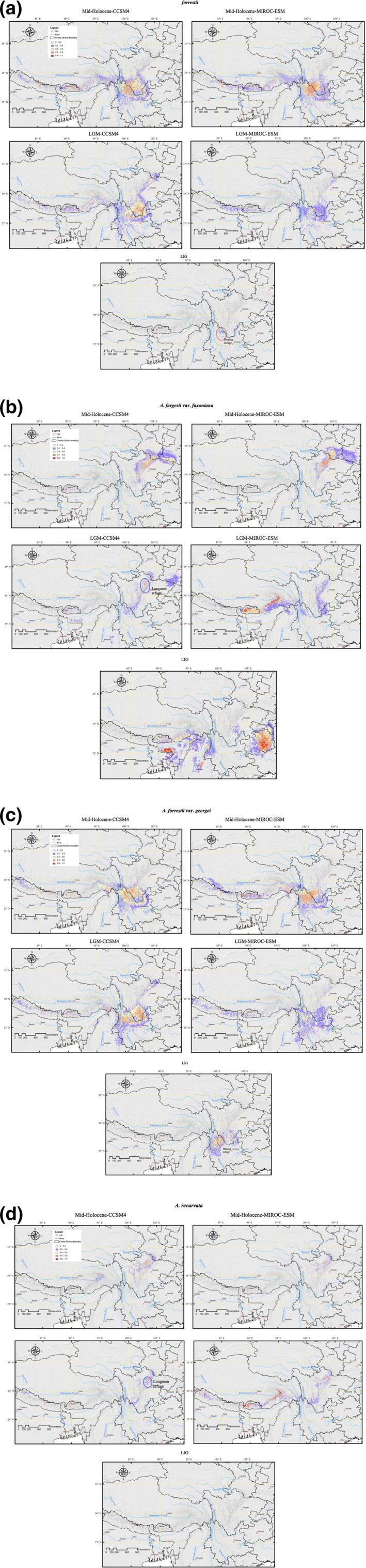
Precited potential distribution for the four *Abies* during the mid‐Holocene and LGM under two general circulation models (CCSM and MIROC) and LIG: (a) *Abies forrestii*, (b) *A. fargesii* var. *faxoniana*, (c) *A. forrestii* var. *georgei*, and (d) *A. recurvata*. The area surrounded by the red circle is Heqing refuge. The area surrounded by the blue circle is Longmen refuge. Refer Figure S3 for more information regarding the potential distribution of *Abies* at Tmax (maximum training sensitivity and specificity) and Tmin (minimum training presence) logistic thresholds

The resulting ENMs provided high AUC scores (*A. forrestii*: 0.986 ± 0.002 (Mean ± *SD*); *A. fargesii* var. *faxoniana*: 0.988 ± 0.002; *A. forrestii* var. *georgei*: 0.986 ± 0.002; and *A. recurvata*: 0.996 ± 0.002 (Figure S2). In comparing the projection for the LGM, based on both CCSM4 and MIROC‐ESM GCM climate models, and that of LIG with the present day (Figures 3 and S3), evidently their general reconstructed distributions were significantly different, but not so for the mid‐Holocene (Figures 2, 3, and S3). In particular, a contraction in the continuity of the four related species' suitable area can be noticed across the southern part of the Hengduan Mountains—along with the Yangtze River, the Mekong River, and the Salween River—and in the eastern TP area. By contrast, an expansion in the northern and western TP between the LIG and the present can be observed (see Figures 2, 3, and S3; Table 2). Corresponding expansions of *A. forrestii* and *A. forrestii* var. *georgei* ca. 21 kyr BP are also well conveyed in the results. A relatively stable temperature range and heavier precipitation (Tables S4 and 3) strongly affected the aforementioned *Abies* distributions (Figures 3 and S3), allowing their extension from glacial refugia with an inclination toward shifting to higher latitudes. Yet, this expansion was mostly constrained over the LIG, which indicated that the warm period could have featured unfavorable climatic conditions for these two *Abies* species (Figures 3 and S3).

**Table 2:** General distributional information of the four *Abies* (*A. forrestii*, *A. faxoniana*, *A. forrestii* var. *georgei*, and *A. recurvata*) as shown in the maps of Figures 2, 3, and S3

Specie	Type	S suitable (≥0.8) area (km^2^)	A Suitable (≥0.6, <0.8) area (km^2^)	B Suitable (≥0.4, <0.6) area (km^2^)	C Suitable (≥0.2, <0.4) area (km^2^)	Total (≥0.2) area (km^2^)	Suitable area (km^2^)	
							Tmin	Tmax
*A. forrestii*	Present	19	48,056	127,222	155,630	330,927	575,193	506,963
	Mid‐Holocene‐CCSM4	8	33,069	114,477	152,431	299,985	554,217	479,717
	LGM‐CCSM4	0	17,896	115,218	160,892	294,006	655,833	545,815
	LIG	0	0	0	5,827	5,827	65,281	45,001
	LGM‐MIROC‐ESM	22	223	18,158	108,716	127,119	376,521	307,458
	Mid‐Holocene‐MIROC‐ESM	962	40,859	86,007	143,554	271,382	537,309	455,632
*A. fargesii* var. *faxoniana*	Present	2	17,371	86,993	156,088	260,454	1,015,485	437,243
	Mid‐Holocene‐CCSM4	6	9,600	47,374	90,092	147,072	886,447	304,109
	LGM‐CCSM4	0	2,499	23,430	85,783	111,712	1,003,468	293,912
	LIG	12,555	59,971	105,214	205,662	383,402	1,418,397	706,712
	LGM‐MIROC‐ESM	6,801	16,282	35,767	117,939	176,789	1,137,294	376,207
	Mid‐Holocene‐MIROC‐ESM	548	13,287	30,093	81,242	125,170	684,203	221,859
*A. forrestii* var. *georgei*	Present	227	38,637	125,680	133,350	297,894	847,235	413,867
	Mid‐Holocene‐CCSM4	0	29,550	127,711	117,458	274,719	907,786	405,818
	LGM‐CCSM4	340	38,778	123,381	153,810	316,309	1,164,859	502,603
	LIG	6	9,529	38,073	57,439	105,047	598,437	180,265
	LGM‐MIROC‐ESM	452	4,094	51,403	167,095	223,044	1,093,381	432,592
	Mid‐Holocene‐MIROC‐ESM	206	43,653	101,185	136,557	281,601	1,154,214	458,615
*A. recurvata*	Present	1,506	7,412	17,444	70,318	96,680	212,514	204,202
	Mid‐Holocene‐CCSM4	54	2,489	7,498	26,551	36,592	92,329	88,000
	LGM‐CCSM4	0	1,857	5,792	23,387	31,036	102,615	95,272
	LIG	0	0	0	0	0	942	587
	LGM‐MIROC‐ESM	3,875	10,539	27,265	63,618	105,297	255,833	245,404
	Mid‐Holocene‐MIROC‐ESM	61	1,551	3,174	9,675	14,461	37,085	35,057

**Table 3:** The top seven (relative basis) contributions of the environmental variables to the MaxEnt model

*Abies forrestii*		*A. fargesii* var. *faxoniana*		*A. forrestii* var. *georgei*		*A. recurvata*	
Variable	Percent contribution (%)	Variable	Percent contribution (%)	Variable	Percent contribution (%)	Variable	Percent contribution (%)
TAR	28.0	PS	21.0	TAR	24.2	TDQ1	30.0
TWQ	13.6	PCQ	18.5	PHQ	22.9	MDR	23.3
Slope	13.4	TAR	15.8	TWQ	19.6	PHQ	16.9
PHQ	13.4	PHQ	15.3	MDR	11.9	Slope	11.3
TDQ	13.1	TWQ	13.2	TDQ	9.6	PS	9.9
PCQ	6.5	TDQ	12.3	Slope	3.9	TWQ	6.2
MDR	6.4	Slope	1.1	Pmax	3.1	PCQ	1.1

Abbreviations: MDR, mean diurnal range; PCQ, precipitation of coldest quarter; PHQ, precipitation of warmest quarter; Pmax, precipitation of wettest month; PS, precipitation of seasonality; TAR, temperature annual range; TCQ, mean temperature of coldest quarter; TDQ, mean temperature of driest quarter; TWQ, mean temperature of wettest quarter.

The stability surfaces steadily predicted an area extending along the central corridor of the *A. forrestii* and *A. forrestii* var. *georgei* distributions, and stretching from the eastern edge of the Salween River, northwards to the western edge of the Yangtze River (Figure 3), hereon referred to as the Heqing refuge (Figures 1 and 3). Habitable areas of *A. forrestii* and *A. forrestii* var. *georgei* were observed in the southern Hengduan Mountains area, where currently there are no *A. forrestii* and *A. forrestii* var. *georgei* trees occurring.

Unlike above, the distribution of *A. recurvata* since the mid‐Holocene stretched to the adjacent mountain chains west of the Hengduan Mountains, and spread to the northeastern edge of the Longmen chain (Figures 1, 2, 3 and S3). This contrasted with the distribution of *A. fargesii* var. *faxoniana*, which had shrunk and shifted somewhat northwards during the LGM (Figures 3 and S3, Table 2). When compared with the LGM, *A. fargesii* var. *faxoniana* displayed a wider and continuous potential distribution area for the present and the mid‐Holocene. In general, the model showed suitable habitats occurring in areas toward both the northern and western parts of the Hengduan Mountains area. A refugial area of *A. fargesii* var. *faxoniana* and *A. recurvata* was inferred, occurring in the north of Sichuan Province, and referred to hereon as the Longmen refuge (Figures 1 and 3). However, much of this Longmen refuge area, which is now forested, was not projected as a suitable area for forests under 120–140 kyr BP prevailing environments, regardless of the forest characterization used (Figures 2, 3 and S3). Therefore, this area was not inferred to have maintained a considerable steady habitat area for *A. fargesii* var. *faxoniana* and *A. recurvata*, but rather to have served as temporary refuge only for these two taxa.

### 3.3 Fossil and phylogeographic records

A classification of the fossils and pollen is given in Table 1. Concurrence and spatial constituencies were found between the potential distribution and pollen records of *Abies* species across the TP (Figures S4 and S5; Table 1). Overall, the pollen of *Abies* species appeared throughout the entire eastern TP, from the Hengduan Mountains to the Longmen Mountains, during the Quaternary period. However, at the LIG, the distribution area of *Abies* almost disappeared entirely from the northern regions of the TP (Figures 3 and S3 and S5).

Unfortunately, at the LIG, only three pollen locations (Heqing Basin, Kunming Basin, Yunnan Province) were able to convey the status of the *Abies* populations in the TP. Hence, the *Abies* refugia during this period cannot be understood well on the basis of paleopalynological evidence alone. Nonetheless, it should be noted that all 11 records agreed with the projections made by the GCMs during the LIG according to Otto‐Bliesner et al. (2006) (Figures S4 and S5).

**Figure 4:**
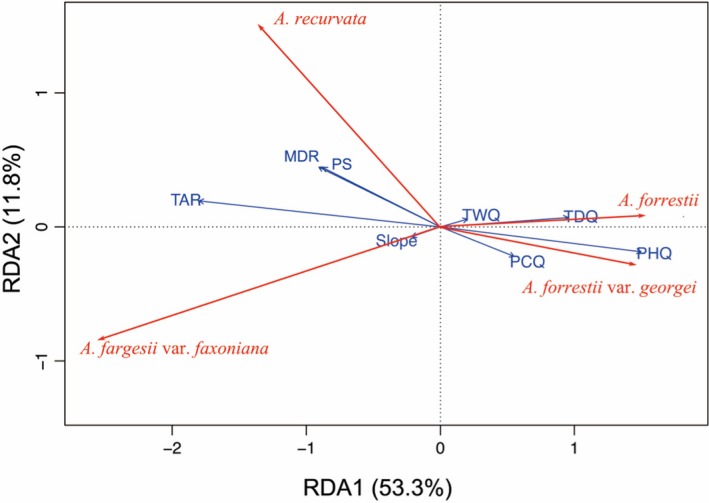
Ordinate plot from redundancy analysis (RDA) on the four *Abies* change trends (red arrows) and their relationships with environmental variables (blue arrows). MDR, mean diurnal range; Pann, annual precipitation; PHQ/WCQ, precipitation of warmest/coldest quarter; PS, precipitation seasonality; TAR, temp annual range; TD, mean temp of driest quarter; Tmin, min temp of coldest month; TWQ/TDQ, mean temp of wettest/driest quarter

Thirty‐two pollen datasets representing the LGM (a period of 21 kyr BP) indicated an expansion of grassland vegetation and other non‐*Abies* plants, which is in line with the predictions from the 21 kyr BP consensus map by the GCMs obtained through the CCSM4 simulation (Table 1, Figures 4 and S4). Only three records (codes 15–17) contradicted the predictions made by the CCSM4 simulation, which projected the occurrence of *Abies* in these regions, while six pollen datasets disagreed with projections made by the MIROC‐ESM simulation. Therefore, the result of the MIROC‐ESM simulation seems to overestimate the forest distribution over the 21 kyr BP period (Figures S4 and S5). The most outstanding fossil evidence was detected in Hongya County (code 5), Xiaohaizi County, and Leibo County (code 10) in the eastern Hengduan Mountains, areas that are located far from the current distribution of these four *Abies* taxa. This finding is of great significance in helping to understand the past distributions of *Abies* trees. Pollen investigations suggested that *Abies* taxa occupied mostly low‐elevation mountain ranges, generally at the base of mountains during cold and dry times, when not undergoing fast expansion to nearby areas during the postglacial recovery. Taken together, these findings suggest that *Abies* persisted in refuges in the Heqing and Longmen areas during the last ice age.

The predictions obtained by combining the 6 kyr BP models produced by the GCMs through CCSM4 and MIROC‐ESM simulations (Table 1; Figures S4 and S5) were inconsistent, as only two pollen records were available. The Hongyuan region, the Zoige Plateau, and Sichuan Province (code 19) are currently situated in the *A. recurvata* forest ecotone, indicating that the forest margin represented in Figure 3c during the mid‐Holocene by the GCMs obtained via MIROC‐ESM had gradually shifted northward. This proposition accords with the pollen and phylogeographic records at this location, reflecting an overall tendency of forest extension to have occurred during 6–10 kyr BP based on the available phylogeographic evidence.

Concerning the *A. fargesii* var. *faxoniana* and *A. recurvata* populations, those from the Longmen refugia exhibited higher cpDNA diversity compared with the nonrefugia areas situated at the south or west of the Longmen Mountains (Figure S6; Table S3). More details regarding the haplotype, haplotype diversity, and nucleotide diversity for each of the sampled locations are included in Table S3. Each of the aforementioned two taxa has a relatively high total genetic diversity (H_T_) and lower within‐population diversity (H_S_), respectively. According to the PERMUY analysis, the N_ST_ exceeded the G_ST_ for each species, and a significant phylogeographical structure was detected for the *A. fargesii* var. *faxoniana* and *A. recurvata* populations (Table S3). Furthermore, Tajima's *D* and Fu's *Fs* tests of neutrality all produced significant negative values (Table S3). These outcomes indicate strong agreement between the observed and expected distributions 9. The elapsed times since the species extension were projected to be 1,542, and 8,086 kyr BP for *A. fargesii* var. *faxoniana* and *A. recurvata*, respectively.

### 3.4 Environmental predictors of the studied *Abies* taxa

Estimates of the relative contribution (percentage) of each environmental predictor used to build Maxent models are presented in Table 3. The pluviometric‐related variables (i.e., PS, PHQ, and PCQ), the thermometric variables (i.e., MDR, TAR, TWQ, and TDQ), and the topographic variables (i.e., slope) all significantly influenced the ENMs. Importantly, they could enhance our understanding of distribution patterns of the four endemic *Abies* taxa. The slope contributed very high gains (>1.0) when used alone for two *Abies* species (Figure S7), meaning that slope was important as climatic factors. However, the respective contributions of environmental variables to the four tree taxa were quite different (Figure S7). The Maxent model showed that TAR was the most significant variable for explaining the distribution of *A. forrestii* and *A. forrestii* var. *georgei*. TWQ and PHQ also played decisive roles in explaining the distribution model of *A. forrestii* and *A. forrestii* var. *georgei*. PS, by contrast, was the key variable explaining the distribution of *A. fargesii* var. *faxoniana*. However, the MDR of temperature and the TDQ were prominent in the distribution model of *A. recurvata*.

The RDA (Figure 4) revealed a clear distinction between the environmental requirements of the four *Abies* taxa, especially on the axes denoting climatic variables (temperature and rainfall), in contrast to the overlapping topographic features between the two habitats. This revealed the TAR and PHQ variables explained 29.0% of the variation in the trends of the four *Abies* taxa. The remaining environmental variables together explained 40.1% of the variation‐species relationships expressed in the RDA model. Its unexplained residual variation may have resulted from integrated effects of other factors (e.g., soil type, land cover, and interspecific competition).

## 4 DISCUSSION

### 4.1 Model accuracy and prediction uncertainty

The calibrated potential distribution models for *A. forrestii*, *A. fargesii* var. *faxoniana*, *A. forrestii* var. *georgei*, and *A. recurvata* attained high AUC values (Figure S2), thus indicating all models had excellent performance (Raes et al., 2014). The modeling technique we used relied on a powerful method (Maxent) that deals strictly with species' occurrence records (Elith & Leathwick, 2009; Phillips et al., 2006). The outcomes from these ENMs might be among the most accurate attainable for the used dataset (occurrence records and environmental data) (Raes et al., 2014). According to the values of the evaluation metrics used, the Maxent model under the CCSM4 simulation was more accurate than that under the MIROC‐ESM simulation at the LGM and during the mid‐Holocene for the study area. However, inadequacies from paleoclimatic scenarios might have increased the uncertainty of these predictive models. For instance, issues could appear because of the appearance of dissimilar climatic settings when ENMs are projected across orthogonal gradients of climatic changes, specifically for those that happened during mid‐Holocene period. In this case, modeling methods will have unfamiliar or erratic performance when used for prediction in those areas (Alba‐Sánchez & López‐Merino, 2010). More issues may have emerged as the GCMs' model data could have undervalued the heating and precipitation fluctuations across the Tibetan Plateau (TP), particularly during the mid‐Holocene period (Li, Wang, & Li, 2013). Therefore, the models presented here could have overestimated the *Abies* distribution during the LGM and the mid‐Holocene (Tinner & Valsecchi, 2013). Yet overfitting should not be construed as low performance, because the models used climatic data alone to predict the potential distributions of the *Abies* taxa, consequently overlooking other contributing factors (e.g., soil type, land cover, and interspecific competition; Wang, Jia, Wang, Zhua, & McDowell, 2017) that might have been involved in shaping the pattern of distributions; this was clearly suggested by the PCA and RDA results (Figures S1 and 4). The spatial resolution of the applied models may have posed an additional constraint, mostly in those regions with intricate topography (Adhikari, Barik, & Upadhaya, 2012). Finally, the local climate may vary greatly from the climate actually being simulated in the corresponding grid box of the models. Improving the models' resolution through explicit consideration of environmental predictors with higher resolution should enhance the depiction of the studied region (Alba‐Sánchez & López‐Merino, 2010). Moreover, the overpredictions of the models for *A. forrestii* and *A. forrestii* var. *georgei* in the western area (Himalaya Mountains area) for the LGM and the mid‐Holocene could be explained by the limited rate of species proliferation. Likewise, in the eastern area of Guizhou Province in the LGM and mid‐Holocene, the overpredictions found there are consistent with its geographic structures possibly acting as plant dispersal barriers (Richards & Bock, 1973; in the case of this research: north–south directional Hengduan Mountains).

### 4.2 *Abies* distribution and refugia based on ENMs, paleorecords, and phylogeography

By combining ecological niche characteristics derived from the environmental characteristics of identified records of Tibetan endemic *Abies* taxa with their paleoecological investigations, we obtained a more refined depiction of the distribution, discontinuities, and segregation among these tree taxa. This will help provide a greater understanding of the ecology and climatic niche of *Abies* taxa and could also contribute to improving relevant climate change mitigation strategies. At the LIG, the combined effects of increasing fluctuations of temperature and precipitation throughout the TP (see Table S4) resulted in a contraction and disintegration of the *Abies* populations (Figures 3 and S3, Table 2). However, these events did not reach the level of severity that would lead to total extinction of the *Abies* taxa. Notably, topography was a critical factor, and this could have driven the shifting of tree populations in the basins rather than in the plains or mountains, albeit under a stable climate (Willis & McElwain, 2002), when seeking to persist and avoid extirpation. Thus, the Heqing Basin region, in addition to providing refugia for other species (Du et al., 2017), had sufficient topographical heterogeneity to provide many suitable microhabitats for the persistence of *A. forrestii* and *A. forrestii* var. *georgei*. Geographical overlapping among the *Abies* taxa was widespread throughout the glacial and interglacial times (Du et al., 2017).

During the LGM, the collective effects of reduced annual precipitation and lower summer and winter temperatures across the TP (Table S4) resulted in a shortened growing season and lowered level of atmospheric CO_2_, down to 200 ppm (Braconnot et al., 2007). In addition, and most remarkably, it apparently caused the contraction and fragmentation of *A. fargesii* var. *faxoniana* and *A. recurvata* populations (Figure 3; Table 3). Conversely, the other two *Abies* taxa expanded their populations in this area during the same period, because smooth fluctuations of temperature and precipitation were more important for them than were changes in other environmental variables (Table 3). Analogous results have been reported for two other tree species at the TP, *Taxus wallichiana* and *Quercus aquifolioides* (Du et al., 2017; Yu, Zhang, Gao, & Qi, 2014). In the Northern Hemisphere, particularly in East Asia and western North America, the movement and shift of northern *Abies* taxa southward to the glacial region and the migration of southern *Abies* taxa northward to the interglacial region have been reported (Alba‐Sánchez & López‐Merino, 2010; Terhürne‐Berson, Litt, & Cheddadi, 2004). Our results contradict that finding, since during the LGM all four endemic *Abies* taxa gradually receded from south to north in the TP. This process of receding may have been caused by Quaternary climatic fluctuations (Xiang et al., 2007). Extensive climatic changes characterized the Quaternary, which involved the repeated development and retreat of glaciers with sporadic warming periods (Owen, 2009). Such a transformation could have created various habitats where the *Abies* species migrated to find niche locations in the TP, whenever the global climate became colder or warmer (Cao et al., 2015). Additionally, the plateau uplifting has formed a natural separation between the temperate and subtropical zones. Further, it would have been difficult for cold air from Siberia and warm humid air from the Pacific Ocean to infiltrate to the central part of the southeastern TP, as the mountains acted as a barrier during the glaciations. In this way, a receding pattern of *Abies* taxa formed from south to north along the TP. Liang et al. (2018) also revealed numerous montane species in the Hengduan Mountains shift to higher elevations not only northward but also westward. The plateau uplifting and climatic fluctuations during the Quaternary overwhelmingly influenced the endemic biome, which shifted from south to north and was associated with glaciation in the late Pleistocene period (Zhang et al., 2000). In parallel, topography was a crucial factor in setting and identifying the range within which populations could expand unimpeded along the continuous mountain ranges (Xiang et al., 2006). In this respect, the Longmen and Hengduan Mountains ranges provided sufficient topographical variability for *Abies* to spread into other regions during the LGM (Figure 3).

During the mid‐Holocene, an extension of the *Abies* taxa populations is well reverberated by their present outcomes, in that those endemic *Abies* taxa displayed larger and continuous potential distribution areas relative to those at the LIG and LGM (see Figure 3). The increased precipitation and warming (Table S4) would have allowed *A. fargesii* var. *faxoniana*, *A. forrestii*, and *A. forrestii* var. *georgei* to spread; however, *A. recurvata* relied less on precipitation for its expansion since the last glacial period (Wang, Xu, et al., 2017). Their areas of habitation, as well as their spread with increasing elevation in the mountainous regions, are features very similar to the contemporary distribution of these taxa. By comparing and relating the three predictions (current ENM, mid‐Holocene ENM, and LGM ENM), we found that the reconstructed distribution patterns differed significantly for each *Abies* taxa, this finding perhaps reflecting the varied responses of each taxa to differing environmental variables (see Figures 2, 3, 4, and S3; Tables 2 and 3).

The ENMs yielded at least four well‐differentiated distribution ranges for the related Tibetan *Abies* species (Figures 2 and 3): *A. fargesii* var. *faxoniana* occurred in the Longmen and Daba Mountains, the range of *A. recurvata* was distributed in the Longmen Mountains and the Yangtze River Basin at the LGM, and both *A. forrestii* and *A. forrestii* var. *georgei* were found along the Heqing Basin during the LIG. Previous studies (e.g., Zhan, 2011) have suggested that western Sichuan Province was probably a refuge for *Abies* taxa since the LIG. The overlap observed between past *Abies* refugia, as drawn from the fossil‐pollen accounts, and the potential distributions of its taxa provide new insights into how *Abies* species were distributed across the TP during the Quaternary period. The palynological data provided evidence for an established ecologically steady area in which conditions safeguarded the residual *Abies* populations, maintaining their survival in the face of the extreme effects arising from the drastic Quaternary climate oscillation. Niche conservatism has been documented throughout the present‐day distributions of *A. forrestii* and *A. forrestii* var. *georgei* (Figures 2, 3, and S4). For the LIG, it was revealed that the area where *Abies* populations had existed extended into the Heqing region and this area corresponded to the presumed refugia (Table 1; Figure 4). This area is considered as a major refugia of Quaternary glaciation for various tree species, namely *Taxus wallichiana*, *Quercus aquifolioides*, *Picea likiangensis*, *Dipentodon*, and *Primula secundiflora* (Du et al., 2017; Gao et al., 2007; Yu et al., 2014). The most divergent hotspot of *A. fargesii* var. *faxoniana* and *A. recurvata* was the Longmen area, where both species harbored high levels of genetic diversity (Figure S6), and the spread of their populations is supported by the significant negative values obtained for all neutrality assessments (Tajima's *D*; Fu's *F*s). We determined that a sudden population expansion for *A. fargesii* var. *faxoniana* and *A. recurvata*, respectively, began ca. 11,542 and 8,086 years ago, in the Longmen Mountains area.

By also considering the indicators of genetic diversity and nucleotide diversity (Zhan, 2011), the populations of *A. fargesii* var. *faxoniana* that attained a high *h* value and a low Pi value had probably experienced an expansion in their distribution following a long‐standing period of suffering from a low effective population size. Accordingly, all of these outcomes converge in supporting the hypothesis that both *A. fargesii* var. *faxoniana* and *A. recurvata* populations at the Longmen areas have expanded (Figure S6). The Longmen Mountains, however, were not projected to have retained an extensive area offering stable habitat for *A. fargesii* var. *faxoniana* and *A. recurvate*, but rather it served as a temporary refuge for both tree species only at the LGM.

### 4.3 Ecological variables affecting the distributions of the four related *Abies* taxa

The four related *Abies* taxa differed significantly in their climatic niche dimensions (Figure 4). This result is consistent with climate variables driving the distribution strategies of closely related plant species; that is, mean temperature of the coldest quarter significantly impacted the distribution of vegetation type (Wang, Xu, et al., 2017) and the minimum temperature of the coldest month was the main limiting factor for the growth of coniferous temperate forests in southwest China (Li, Peng, & Higa, 2016). In contrast, it was reported that *Abies* species were cold‐tolerant, hydrophilic, sensitive to high temperature, and intolerant to aridity (Wang, Jia, et al., 2017). Such inconsistent results may arise from differences in the adaptation strategies of endemic *Abies* species to the environmental variables (Table 3; Figure S7). Also, divergent and convergent strategies might lead to an over‐ or underestimation of the suitable distribution area for combinations of several different co‐occurring species, as noted by Willis and McElwain (2002) and Du et al. (2017). Our RDA suggested that *A. fargesii* var. *faxoniana* and *A. recurvata* were better capable of adapting to more magnitude of fluctuation in temperature and precipitation, while the other two taxa are adapted to the higher precipitation of the Eastern Tibetan Plateau (Figure 4). Still, the effect of temperature fluctuations on *Abies*' distributions was greater than that of either increases or decreases in temperature alone (Table 3). This conclusion is consistent with the current distribution of these four endemic *Abies* taxa: In the south, *A. forrestii* and *A. forrestii* var. *georgei* occur in wetter areas, while in the north, *A. fargesii* var. *faxoniana* and *A. recurvata* occupy an area of the TP that undergoes greater temperature magnitude of fluctuation (He et al., 2015; Wang, Jia, et al., 2017). Since most studies tend to focus on the impacts of climatic changes, they simulate increases in average temperature, thereby ignoring the effects of changes in magnitude of fluctuation of temperature (Gazol et al., 2015; Koo et al., 2017; Tinner & Valsecchi, 2013; Xiong et al., 2016). Our results suggest the importance of temperature fluctuations for affecting the growth and distribution of trees, and this variable should be considered when assessing the effects of future changes in climate on plant species. Slope is typically an important ecological factor that influences the distribution of local plant species (Adhikari et al., 2012). Consistent with other regional studies and investigations (Adhikari et al., 2012; Alba‐Sánchez & López‐Merino, 2010), we found that slope was an important contributor to the distribution of *Abies* taxa studied here across the TP.

## 5 CONCLUSIONS

The strong agreement of paleorecords with our model predictions demonstrated the merit of using the Maxent modeling approach for projecting the distributions of endemic *Abies* taxa in the TP. The overall reconstructed distributions of the *Abies* taxa differed dramatically going from the late Pleistocene to the present. All the endemic *Abies* taxa at the TP exhibited a pattern in that they receded from south to north during the late Pleistocene. In the case of *A. fargesii* var. *faxoniana* and *A. recurvata*, the Longmen refuge served only as a temporary refuge for them since the LGM, whereas both *A. forrestii* and *A. forrestii* var. *georgei* were found distributed throughout the Heqing refuge since the LIG. The annual temperature range was the most important variable for explaining the distributions of *A. forrestii* and *A. forrestii* var. *georgei*. However, seasonal precipitation and the mean temperature of the driest quarter contributed significantly to the distribution model for *A. fargesii* var. *faxoniana* and *A. recurvata*, respectively. Further, the slope also contributed substantially to the above *Abies* distribution models. Varied adaptation strategies might well be the reason for differences found in the past and current potential distribution patterns of these four related *Abies* taxa at the TP. Incorporating ecological niche characteristics from *Abies* species combined with their paleoecological investigations provides a useful approach to better understand species migrations under climate changes. It could help guide rational management strategies of forests whose keystone species exist in high‐altitude regions. Future work should focus on whether other endemic tree species (such as *Picea*, *Metasequoia*), used the same refuge as, for example, the Heqing refuge from this study.

## CONFLICT OF INTEREST

The contributing authors declare no conflict of interests regarding the publication of this article.

## AUTHOR CONTRIBUTIONS

All authors worked together to design this study. Q.X. and F.Z. collected the data. Q.X., M.A.D., and B.P. carried out the analysis. Q.X., K.P., L.Z., and M.W.A.H. wrote the draft of this manuscript. All authors contributed considerably to modify and revise this manuscript.

## Data Availability

All generated or analyzed data of this study are given in this published article and Appendix S1 provided.
