# Supplementary material for: Concealed truth: Modeling reveals unique Quaternary distribution dynamics and refugia of four related endemic keystone Abies taxa on the Tibetan Plateau
Source: Ecol Evol. 2019 Dec 4;9(24):14295–316. doi: 10.1002/ece3.5866 (PMC6953664; doi:10.1002/ece3.5866)
Supplement: Supplementary file 1 [file ECE3-9-14295-s001.docx]

**Supporting information**

# Additional Reference List

Bao, R. (2010). The characteristics of Monsoon: Evidence from between western and eastern sample plots beside Gaoligong Mountain in Yunnan over the past 60 ka and their comparason with oceanic records. China University of Geosciences, Beijing (in Chinese).

Cai, Y. (2008). Study on environmental change in Zoige Plateau：Evidence from the vegetation record since 24000 a B.P. the Chinese Academy of Sciences, Beijing, 1-76 (in Chinese).

Chen, H.J. (2011). Responses of functional traits of *Abies faxoniana* to elevated atmosphere CO_2_ concentration and temperature. Sichuan Agricultural University, Chengdu (in Chinese).

Chen, L.R. (1985). Palynological assemblages and palaeoenvironment of the Holocene in Yihai Mianning, Sichuan. *Acta Geologica Sichuan*, **3**, 127–129 (in Chinese).

Chen, P.Y. (1987). Palynological assemblages and palaeoenvironment of CK17 drilling Hole in Caohai (Grassy Sea), Weining County, Guizhou Province. *Geology of Guizhou*, **3**, 381–390 (in Chinese).

Chen, P.Y. (1989). Holocene sporo-pollen associations and palaeoenvironment of Jiulongchi Section of Fanjing Mountain, Guizhou Province. *Geology of Guizhou*, **6**, 110–116 (in Chinese).

Chen, P.Y., Lin, S.J. & Zhou, Q.Y. (1993). Study of a peat-bed section near Zhao Jiayuanzi in Caohai area, Guizhou. *Guizhou Science*, **11**, 31–39 (in Chinese).

Chen, P.Y., Zhou, Q.Y., Lin, S.J., Qiao, Y.L. & Sun, Y.M. (1992) The changes of vegetation and climate of Jiulongchi section of Fanjingshan Mountain in Guizhou Since 10000 Years Ago. *Guizhou Geology*, **2**, 167-177 (in Chinese).

Chen, X.M., Li, R.W. & Wang, L.H. (2005). Researches on the niche of the major tree and shrub species in vegetation communities of sub-alpine Forests in the upper reaches of the Minjiang River. *Journal of Sichuan Forestry Science and Technology*, **26**, 12–19 (in Chinese).

Cheng, J.W. (2010). Evolution of terraces I–III along the Anning River, western Sichuan, based on pollen records and terrace structure. *Scientia Sinica (Terrae)*, **40**, 1410–1419 (in Chinese).

Cheng, J.W., Gong, H.L. & Guo, X.L. (2010). Pollen records reflecting paleoclimate chang since Late-pleistocene in the Terraces I-III of Anning River in Western Sichuan Province. *Northwestern Seismological Journal*, **32**, 349–357 (in Chinese).

Cheng, W., Wu, N. & Luo, P. (2005). Survival analysis of *Abies faxoniana* population near timberline on the upper Minjiang River. *Acta Phytoecologica Sinica*, **29**, 349–353 (in Chinese).

Dang, C.L., Wu, Z.L. & Wang, C.Y. (1994). Studies on biomass and net primary production of *Abies georgei* Community. *Journal of Yunnan University (Natural Sciences)*, **16**, 214–219 (in Chinese).

Dong, G.B., Wu, R.J., Wu, Y.H., Shi, Y., Liu, Z.M. & Li, Y.C. (2000). Vegetation and environmental variations since Holocene in Mianning area, Sichuan province. *Acta Micropalaeontologica Sinica*, **7**, 470–477 (in Chinese).

Gan, L.M. (2014). Simulation and analysis of forest landscape dynamics in the western margin of Sichuan Basin. Sichuan Agricultural University, Chengdu (in Chinese).

Guo, B.D., Zhang, Y.D. & Wang, X.C. (2016). Response of *Picea purpurea* and *Abies faxoniana* tree rings at different slope aspects to rapid warming in western Sichuan, China. *Chinese Journal of Applied Ecology*, **27**, 354–364 (in Chinese).

Guo, C.X., Luo, F., Luo, C.X., Feng, S., Sun, Q., Yang, Z.R. & Zhang, J. (2012). Holocene pollen record in the Tangke peat profile of Sichuan and its palaeoclimatic significance. Acta *Palaeontologica Sinica*, **3**, 351–358 (in Chinese).

Han, J.E., Yu, J., Zhu, D.G., Shao, Z.G., Meng, X.G., Lu, R.P., Qian, C., He, C.G. & Wang, J. (2011). The palaeoenvironmental evolution of the Yellow River headwater basin in Qinghai Province since Early Pleistocene. *Geological Bulletin of China*, **12**, 1941-1949 (in Chinese).

Han, Q.J., Sun, X.G. & Yang, L. (2005). Scale affection and its fractal characteristics about a-diversity in *Picea-Abies* forests and coniferous broad-leaved mixed forests. *Journal of Gansu Agricultural University*, 1, 1–6 (in Chinese).

Han, Y.H. & Yu, J.B. (1988). Pollen analysis and palaeoenvironment study of late Pleistocene in Daganba, Guizhou Province. *Journal of Integrative Plant Biology*, **30**, 76–84 (in Chinese).

Hao, H., Fergusion, D.K., Chang, H. & Li, C.S. (2009). Vegetation and climate of the Lop Nur area, China, during the past 7 million years. *Climatic Change*, **113**, 323–338.

He, F.Y., He, F. & Wu, Z.D. (2015). Change of physical properties along elevation gradients in *Abies faxoniana* natural forest. *Journal of Northwest Normal University (Natural Science)*, **51**, 92–98 (in Chinese).

He, X.B., Liang, Y.M. & Tian J.L. (2000). Pollen Analysis of Holocene Loess and Vegetation Evolution in Yan'an Area. *Research of Soil & Water Conservation*, **2**, 124-127 (in Chinese).

Huang, B.X., Bai, X.Z. & Yang, X.J. (2012). Pollen record and paleovegetation evolution in 13.6 ka B.P. Peat profile in Hongyuan Area of Sichuan Province. *Shandong Land and Resources*, **28**, 534–538 (in Chinese).

Huo, X.P., Li, X.W. & Zhang J. (2009). Experimental on soil storage capacity and infiltration capability under different vegetation types in the subalpine of Western Sichuan. *Science of Soil and Water Conservation*, **7**, 74–79 (in Chinese).

Jiang, X.B., Li, J.M., Gao, T.Y., Xu, G.S. & She, S.Q. (2000). Evolution history and distribution of spruce. *Forest Investigation Design*, **1**, 30–33 (in Chinese).

Ju J.L. (2012). Conditions of moisture and its energy under Subalpine *Abies faxoniana* in Western Sichuan. Sichuan Agricultural University, Chengdu (in Chinese).

Kuang, M.S., Xie, S.Y. & Zeng, Y. (2002). Study on the Palaeovegation and Palaeoclimate since late Pleistocene in the Diancang Mountain Area in Dali of Yunnan Province. *Journal of Southwest China Normal University (Natural Science)*, **27**, 759-765 (in Chinese).

Lai, S.D., Liao, J.G. & Liu, W.Y. (1988) The transpiration rate of *Abies georgei* and the correlation with environmental factors. *Acta Botanica Sinica*, **30**, 639–643 (in Chinese).

Li, B.C. (2005). Characteristics of Holocene paleo-climate in Lantian, Shaanxi, China. *Journal of Chengdu University of Technology* (*Science and Technology Edition*), **6**, 587-591 (in Chinese).

Li, B.C. & Ke, M.H. (2006). Vegetation and climate during the Late Pleistocene in Fu County , Shaanxi. *Journal of Arid Land Resources and Environment*, **5**, 173-176 (in Chinese).

Li, B.C. & Sun, J.Z. (2005) Vegetation and climate environment during Holocene in Xi’an region of Loess Plateau, China. *Marine Geology & Quaternary Geology*, **25**, 125-132 (in Chinese).

Li, C.H., Tang, L.Y. & Feng, Z.D. (2006) High-resolution pollen records and the climatic changes it reflects in the late stage of Late Pleistocene in the Jingning area, Gansu. *Science in China* (Series D: Earth Sciences), **5**, 453-460 (in Chinese).

Li, H.T. (2014). Sedimentary characteristics and paleoenvironmental evolution of Renacuo Lake at Quaternary in the area of Gaize, Tibetan Plateau. China University of Geosciences, Beijing (in Chinese).

Li, L., Wang, J.X. & Li, Y.B. (2013). The ancient climate change research progress based on stalagmite records of Yunnan-Guizhou plateau. *Yunnan Geographic Environment Research*, **5**, 96–103 (in Chinese).

Li, W. (2008) Comparative study on climate multi-index of the late interglacial period in luochuan area. Capital normal university, Beijing (in Chinese).

Li, W.H. (1982). An introduction to Tibetan dark coniferous forest. *Natural Resources*, **2**, 1–16 (in Chinese).

Liao, J. (2017). Vegetational and environmental changes during Last Glacial Maximum (LGM) in Lugu Lake.Yunnan Normal University, Kunming (in Chinese).

Lin, B. (2006). Response and acclimation to different and varing light environment of four tree species seedings in *Abies faxoniana* community. Chengdu Institute of Biology, Chinese Academy of Sciences, Chengdu (in Chinese).

Liu, B. (2013). Soil-forming process and features of subalpine fir (*Abies faxoniana*) forests, Western China. Sichuan Agricultural University, Chengdu (in Chinese).

Liu, B.N. (1989). Effective storage period of three alpine coniferous species in Yulong Mountain. *Yunnan Forestry Science and Technology*, **4**, 29–30 (in Chinese).

Liu, G.X., Shen, Y.P. & Wang, R. (1995). The vegetation and climatic changes in Zoige during the last 20000 years determined by pollen records. *Journal of Glaciolgy and Geocryology*,**17**, 132-137 (in Chinese).

Liu, H.L., Li, C.B. & Chen, L.R. (2003). Researches on combinative characteristics of spore-pollens and analysis of ancient climate in Huolie Lake since 22 Thousand Years ago. *Journal of Sichuan Forestry Science and Technology*, **24**, 1–5 (in Chinese).

Liu, H.L., Li, C.B.& Chen, L.R. (2004). Researches on combinative characteristics of spore-pollens and analysis of ancient climate in small lake since 16 thousand years ago. *Journal of Sichuan Forestry Science and Technology*, **25**, 5–9 (in Chinese).

Liu, J.F. (1989). The sporo-pollen assemblage and their significance of the first layer paleosol in the Loess strata of Guangzhong, Shaanxi Province. *Journal of Northwest University* (*Natural Science Edition*), **1**, 77-80 (in Chinese).

Liu, J.F. (1992). The sporepollen records of glaciation-loess cycles in huining area in the west part of the Loess Plateau since 660000 a B.P. *Glacial Permafrost*, **1**, 33-43 (in Chinese).

Liu, J.F. & Su, Y. (1994). The changes of vegetation and climate in Pingliang region, Gansu Province since about 800000 YR B.P. *Geographical Research*, **4**, 90-97 (in Chinese).

Liu, L.H., Yu, Y.D. & Zhang, J.H. (1984). The division of vertical vegetation zone in Hengduan Mountain. *Acta Botanica Yunnanica*, **6**, 205–216 (in Chinese).

Liu, W.Y., Lai, S.D. & Liao, J.G. (1991). Study on photosynthesis of *Picea brachytyla* var. *complanata* and *Abies georgei*. Acta Botanca Yunnanica, 13, 425–431 (in Chinese).

Liu, Y.C., Fang, R.J. & Zhou, B.T. (1991). An analysis of the carpophytis floral composition in the areas of Jiuzhai Gou nature sanctuary. *Journal of Southwest China Teachers University (Natural Science)*, **16**, 471–478 (in Chinese).

Liu, Z.G. & Qiu, F.Y. (1986). The main vegetation types and their distribution in the Gongga Mountain. *Acta Phytoecology ET Geobotanica Sinica*, **10**, 26–34 (in Chinese).

Lu, K.L. (1983). Studies on the decay of *Picea* and *Abies* in northwest Yunnan Province. *Yunnan Forest Inventory and Planning*, **3**, 33–35 (in Chinese).

Luo, L.C. (2008) Physical and mechanical properties of nine coniferous species in Yunnan. *Journal of West China Forestry Science*, **37**, 5–11 (in Chinese).

Luo, C.K. (1981). Investigation on regeneration of *Spruce* and *Abies* in alpine dark coniferous forest region in western China. *Yunnan Forestry Science and Technology*, **1**, 1–23 (in Chinese).

Luo, L.P., Zhu L.D., Xiang, F., Yang, W.G., Kan, A.K., Luo, H., Zhang, Q. & Zhu Z.Y. (2008). Spore-pollen assemblage and environmental changes of the Chengdu Plain during the late Holocene. *Acta Palaeontologica Sinica*, **47**, 195–202 (in Chinese).

Ma, Y.Z., Zhang, H.C. & Li, J.J. (1998). On the evolution of the palynoflora and climatic environment during late pleistocence in Tengger Desert, China. *Acta Botanica Sinica*, **40**, 871-879 (in Chinese).

Mao, X. (2011). Preliminary study on lacustrine sediments at Diexi in the upper reach of the Minjiang River during the last Deglaciation. China University Of Geosciences, Beijing (in Chinese).

Mao, Z.L. (1991). Sporo-pollen associations and palaeoenvironment of CK2 Hole in Xiachahe of Haizi Farm, Anlong, Guizhou Province. *Geology of Guizhou*, **8**, 93–96 (in Chinese).

Pan, W.J., Yu, J.X. & Hao, C. (2013). Vegetation succession and environmental transition during Late Pleistocene in Milin Area, Tibetan Plateau. *Geological Science & Technology Information*, **32**, 6-11 (in Chinese).

Schlütz, F. & Zech, W. (2004). Palynological investigations on vegetation and climate change in the Late Quaternary of Lake Rukche area, Gorkha Himal, Central Nepal. *Vegetation History and Archaeobotany*, **13**, 81–90.

Shen, J.M. (2008). The cause of groundwater salinization and paleoclimate characteristics in Yanchi area of Jiuquan East Basin.Chinese Academy Of Geological Sciences, Beijing (in Chinese).

Shi, S.Q., Yuan, D.X., Luo, L.D., Hao, X.D. & Zhao, Z.Y. (2011). Comparative study on climatic change during later period of the late Pleistocene epoch with the sporopollen record from northwest Yunnan plateau and the stalagmite record from South China. *Carsologica Sinica*, **2**, 119–127 (in Chinese).

Shi, S.Q., Yuan, D.X., Luo, L.D.,Zhao, Z.Y. & Hao, X.D. (2012). Sporopollen records and climate changes since 35, 000 a B.P. Iin Hongya, Sichuan Province. *Carsologica Sinica*, **2**, 121–130 (in Chinese).

Shi, S.Q. (2012). Pollen records from northwest Yunnan plateau and west Sichuan mountain and paleoclimate significance since later period of the late Pleistocene. Southwest University, Chongqing, pp. 1–91 (in Chinese).

Sichuan Forest Editorial Board. (1992). Sichuan Forest. China Forestry Press, Beijing (in Chinese).

Su, W.H. & Peng, J.S. (2014). Tropical and temperate forest vertical landscape in Hengduan Mountain area. *Forest and Humankind*, **5**, 84–91 (in Chinese).

Sun, L., Li, X.Q., Shang, X., Zhou, X.Y. & Dodson J. (2014). Vegetation characteristics and palaeoclimate of Xiahe site in the Southern Loess Plateau during the Mid-Holocene based on fossil charcoal records. *Quaternary Sciences*, **1**, 27–34 (in Chinese).

Tang, L.Y. & An, C.B. (2007). The study on the changes of vegetation and the spore pollen of the new world vegetation in the loess plateau of longchina. . *Natural science progress*, **10**, 1371-1382.

Tang, L.Y., Li, C. & An, C.B. (2007). Vegetation history of the western Loess Plateau of China during the last 40ka based on pollen record. *Acta Palaeontologica Sinica*, 2007, 46, 45-61 (in Chinese).

Tang, L.Y., Shen, C.M., Liao, G.B. & Overpeck, J.T. (1999). New High Resolution Pollen Records from Two Lakes in Tibet. *Acta Botanica Sinica*, **8**, 896–902 (in Chinese).

Wang, L., Cai, X.H. & Wang, Y. (2013). Spatial structure of Scenic Forest of *Abies faxoniana* in Western Sichuan. *Forest Research*, **26**,622–627 (in Chinese).

Wang, P.F., Xia, Y.M. & Wang M.H. (1981). Discussion on the evolution of the new peat peat in southern Tibet. *Geological Sciences*, **2**, 144-152+189-191 (in Chinese).

Wang, X.Q. & Wang, L.S. (2013) The pollen and spore characteristics of the Diexi ancient dammed lake on the upstream of Minjiang River. *Earth Science* (*Journal of China University of Geosciences*), 38, 975-982 (in Chinese).

Wang, Y., Zhao, Z.Z., Qiao, Y.S., Wang, S.B., Li, C.Z. & Song L.F. (2006). Paleoclimatic and paleoenvironmental evolution since the late glacial epoch as recorded by sporopollen from the Hongyuan peat section on the Zoige Plateau, northern Sichuan, China. *Geological Bulletin of China*, **25**, 827–832 (in Chinese).

Wei, H.C., Ma, H,Z., Pan A.D. & Shan, F.S. (2009). Vegetation and climate changes based on the pollen records in Gansu Qinghai Area since the Last Deglaciation. *Salt lake study*, **17**, 13-18 (in Chinese).

Wu, B.L., Zhang, P.Y., Chen, H.L., Xu, X.H., Pan, W.S. & Luo, S.H. (1985). Detailed talk about g flat. In Dingxi, Gansu Province in central region since forty thousand combined pollen and vegetation change. *Journal of Gansu forestry science and technology*, **4**, 11-17 (in Chinese).

Wu, Z.H., Wu, Z.H., Ye, P.S., Hu, D.G. & Peng H. (2006). Late Cenozoic environmental evolution of the Qinghai-Tibet Plateau as indicated by the evolution of sporopollen assemblages. *Geology in China*, **5**, 966-979 (in Chinese).

Xia, B., Lan, T. & He, S.A. (1996). Canopy gaps in subalpine spruce-fir forests of the hills around Bitahai Lake, Yunnan Province. *Journal of Plant Resources and Environment*, **4**, 3–10 (in Chinese).

Xiao, X.Y., Shen, J., Xiao, H.F. & Tong, G.B. (2006). Pollen records and vegetationand climate changes in Heqing Basin, Yunnan Parovince Pleistocene. *Journal of Lake Sciences*, **18**, 369–376 (in Chinese).

Xu, Z.P., Chen, J.Q. & Xiao, J.Y. (2009). Pollen records since Late Middle-Pleistocene in the Kunming Basin, Yunnan Province and paleoclimate evolution. *Acta Geologica Sinica*, **83**, 65–77 (in Chinese).

Xiong, X.J. (2009). Effects of seasonal freeze-thaw on soil microbial activity in the subalpine fir forest. Sichuan Agricultural University, Chengdu (in Chinese).

Xu Q.H., Yang X.L. & Wang Z.H. (1998). Pollen analysis on a peat section at Ganlan Basin, Xishuangbanna, Yunnan Province. *Yunnan Geographic Environment Research*, **2**, 36-39+43-44 (in Chinese).

Ye, C.Y., Wang, Z.M. & Zhao, S.Q. (2013). Priliminary study of the relationship between pollen assemblages and Uranium distribution in Gasikule Salt Lake’s Sediments, Qaidam Basin. *Geological Review*, **59**, 479-488.

Ye, Y.Y., Yan, F.H. & Liu, Y.X. (1986). The sporo-pollen analysis of late Quaternary deposites from Dukou region, Sichuan Province, and its geological siginificance. *Scientia Geologica Sinica*, **4**, 392–402 (in Chinese).

Yu, J. (2008). The quaternary environmental evolution of typical lakes in central tibetan plateau. Chinese Academy of Geological Sciences, Beijing (in Chinese).

Yu, Y., Cao, M. & Liu, W.S. (2003). Diversity of subalpine coniferous forests and *Quercus* *sclerophyllous* Forests in Northwest Yunnan. *Journal of Mountain Science*, **21**, 568–575 (in Chinese).

Yu, Z.Z., Zhen, Y.J. & Zhou, Z.H. (1989). Selective adjustment of forest management and harvesting. *Forest Investigation Design*, **2**, 11–19+10 (in Chinese).

Zhang, G.B. (2008). Forest carbon storage dynamics at the upper stream of Minjiang River. Chinese Academy of Forestry, Beijing (in Chinese).

Zhang, F.H., Wu, X.X., Cai, X.H. & Wang, C. (2015). The influence of three different forest types on soil physical and chemical properties in subalpine areas of Western Sichuan. *Journal of Sichuan Forestry Science and Technology*, **36**, 8–12 (in Chinese).

Zhang, H., Xiao, X.Y., Yang, X.D., Long, H. & Zhang, M.H. (2016). The relationship between the vegetation and the modern pollen assemblages from the Lugu Lake in Yunnan Province, southwest China and the surrounding mountains. *Acta Micropalaeontologica Sinica*, **33**, 304–315.

Zhang, J.S. (2014). Study on different types of vegetation biomass in Beichuan Nature Reserve. Sichuan Agricultural University, Chengdu (in Chinese).

Zhang, Q.D., Xie, Q. & Yang, H. (2013). Study of water Conservation capacity litter and soil of *Abies faxoniana* in Siguniang Mountain. *Sichuan Environment*, **32**, 42–45 (in Chinese).

Zhang, Q.Y. (2006). Ecological Characteristics of timberline tree population in Eastern Qinghai-Tibetan Plateau. Chinese Academy of Sciences, Beijing (in Chinese).

Zhang, W., Liu, B.B., Li, Y.H., Feng, J., Zhang, B., Wang, Z.L. & Li, D.P. (2012). Quaternary Glacier development and environmental evolution in Qianhu Mountain, Northwestern Yunnan Province*. Acta Geographica Sinica*, **5**, 657–670 (in Chinese).

Zhao, J.B. & Huang, C.C. (1999). Environmental change of Late Pleistocene in Loess Plateau of Shaanxi Province. *Scientia Geographica Sinica*, **6**, 565-569 (in Chinese).Zhao, R. (2014). Research on the numeric dynamics of *Abies georgei* population under climate change. Kunming University of science and Technology, Kunming (in Chinese).

Zhao, X.T., Wu, Z.H. & Hu, D.G. (2005). The discovery of Late Pleistocene highstand lacustrine sediments of the Co Ngoin Lake and Adjacent Areas, Tibet. *Acta Geoscientica Sinica*, **4**, 291-298 (in Chinese).

Zhao, Y.G. & Gao, K.S. (1989). Research on the *Abies georgei* growth law. *Yunnan Forestry Science and Technology*, **1**, 24–32 (in Chinese).

Zhou, W.J., Liu, Z., Wang, H. & Liu, S.Y. (2011). The pollen record from the Hongyuan peatland in the Tibetan Plateau since 13500 year. *Journal of Earth Environment*, **5**, 605-612 (in Chinese).

Zhu, W.X. & Li, P. (1994). The sporopollen groups of the Atherupus Macrourus Dunghill in Zhijin cave and their geological siginificance. *Carsologica Sinica*, **13**, 256–260 (in Chinese).

Zhu Y., Chen F.H. & Madsen B.D. (2001). The records and environmental significance of early Holocene lake spores in the river basin [J]. *Scientific Bulletin*, **19**, 1596-1602 (in Chinese).

**Methods**

Population genetic diversity was quantified using indices of nucleotide diversity (Pi) and haplotype diversity (*h*) using the program ARLEQUIN v3.5 (Excoffier and Lischer, 2010). Average gene diversity within populations (H_S_), total gene diversity (H_T_) and between-population differentiation (the coefficient of genetic differentiation over all populations (G_ST_) and the measure of genetic differentiation that incorporates phylogenetic distance (N_ST_)) was estimated using the program PERMUT ([www.pierroton.inra.fr/genetics/labo/software/](http://www.pierroton.inra.fr/genetics/labo/software/)) with 10,000 permutation tests. A higher N_ST_ than G_ST_ indicates the presence of phylogeographical structure (Zhang *et al*., 2005). The statistical tests of Tajima’s D and Fu’s Fs values were used to detect the deviation from the neutral model of evolution using DnaSP 5.10. Tajima’s D uses mutation frequencies in the sequences to identify if a population has undergone a recent population expansion event, and is determined by the difference between the average number of nucleotide differences and the number of segregating sites estimated from pair-wise comparisons (Tajima, 1989). Similarly, Fu’s F_s_ test is more sensitive to recombination thus it helps in detecting population growth and genetic hitchhiking (Fu, 1997). The mismatch distribution for cpDNA trnL-F fragment was analysed by DNSP ver.5 software. The distribution of the observed points showed a single peak, and the overall trend gradually decreased. The populations of two *Abies* taxa (*A. fargesii* var. *faxoniana* and *A. recurvata*) were both fiting for the rapid expansion model, and the time of population expansion could be calculated (Rogers and Harpending, 1992). We used $t=\tau/(2\mu)$ [Equation (1)] to calculate the expansion time of *A. fargesii* var. *faxoniana* and *A. recurvata*, where $\tau$ is mutational time (Rogers and Harpending, 1992).

**Figure captions**

**Figure S1** PCA analysis (standardized and centered) of 19 bioclimatic variables, aspect and slope for present-day climatic conditions of the four *Abies* taxa in the Tibetan Plateau.

**Figure S2** The results of the AUC curves in developing a habitat suitability model for the four *Abies* (*Abies forrestii*, *Abies forrestii* var. *georgei*, *Abies fargesii* var. *faxoniana* and *Abies recurvata*). The red (testing) line indicates the “fit” of the model to the test data and is the real test of the model’s predictive power. The test was simulated ten times. The blue areas represent the standard deviation.

Figure S3 Maxent range maps for the four *Abies* taxa in the Tibetan Plateau, based on models of 200 replicates with random subsampling. Dark gray indicates presence where Tmin = Minimum training presence logistic threshold. Black indicates presence where Tmax = Maximum training sensitivity plus specificity logistic threshold.

**Figure S4** Potential distribution of the four *Abies* for Minimum training presence logistic threshold (yellow) and Maximum training sensitivity plus specificity logistic threshold (green) during the mid-Holocene and LGM under two different general circulation model (GCM) estimates (CCSM4 and MIROC-ESM) and LIG. The black dots shows the pollen and fossils deposits of *Abies* taxa along with the code number (Details on code numbers of *Abies* pollen were specified in Table 2).

**Figure S5** Additional information concerning the potential distribution of the four *Abies* at the LIG under Otto-Bliesner *et al*., 2006, LGM and mid Holocene under two GCM estimates (CCSM4 and MIROC-ESM). Pollen deposits during the LIG, LGM and mid Holocene (± 500 yr) with the code indication are shown (The details of pollen deposits are indicated in Table 2). Yellow indicates presence where Tmin = Minimum training presence logistic threshold. Green indicates presence where Tmax = Maximum training sensitivity plus specificity logistic threshold.

**Figure S6** Geographic distribution of *A. fargesii* var. *faxoniana* and *A. recurvata* inferred from phylogeography results (Population codes, numbers and haplotypes correspond to those in Table 3).

**Figure S7** The results of the jackknife test of the variables’ contribution in modelling the distribution of suitability of the four *Abies* taxa. The dark blue bars indicate the gain from using each variable in isolation, the light blue bars indicate the gain lost by removing the single variable from the full model, and the red bar indicates the gain using all the variables. bio2, mean diurnal range; bio7, temperature annual range; bio8, mean temperature of wettest quarter; bio9, mean temperature of driest quarter; bio13, precipitation of wettest month; bio14, precipitation of driest month; bio15, precipitation of seasonality; bio18, precipitation of warmest quarter; bio19, precipitation of coldest quarter.

**
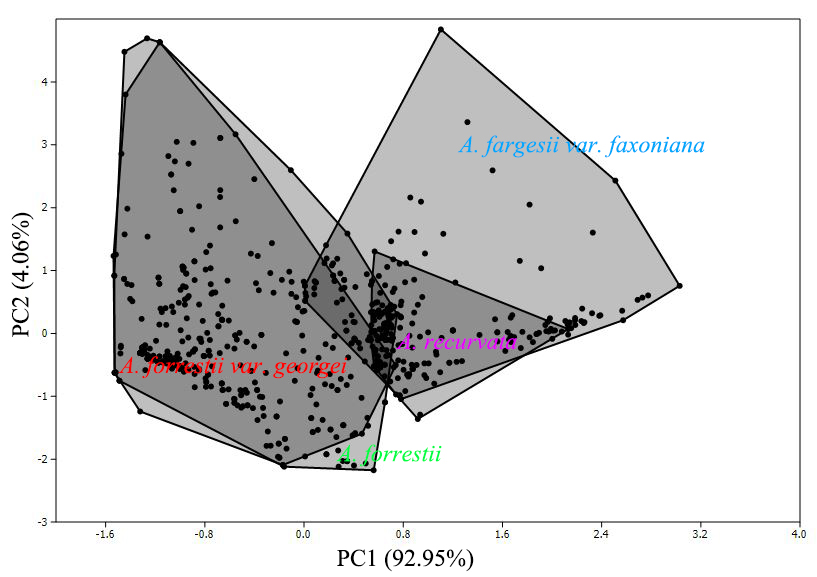
**

**Figure S1**

**
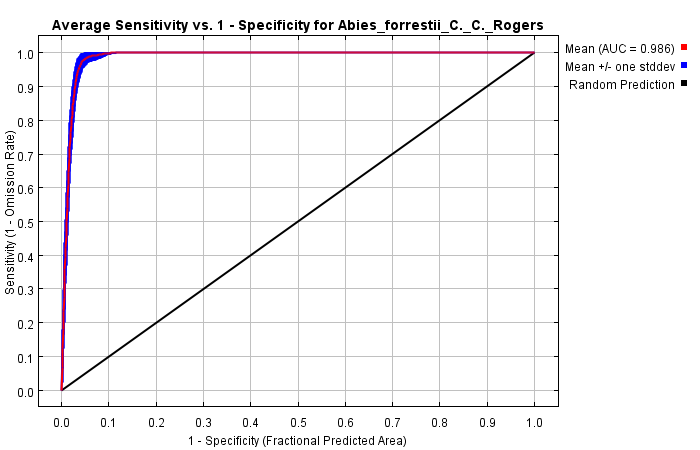

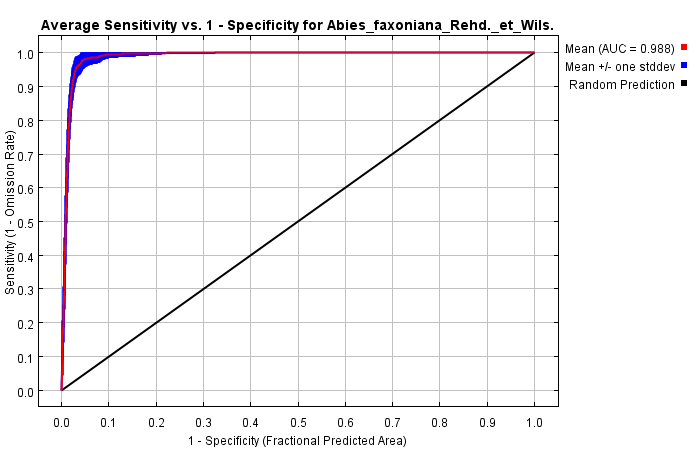

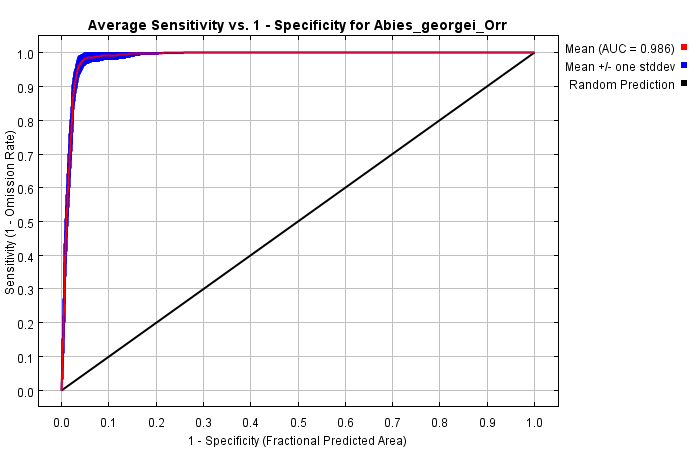

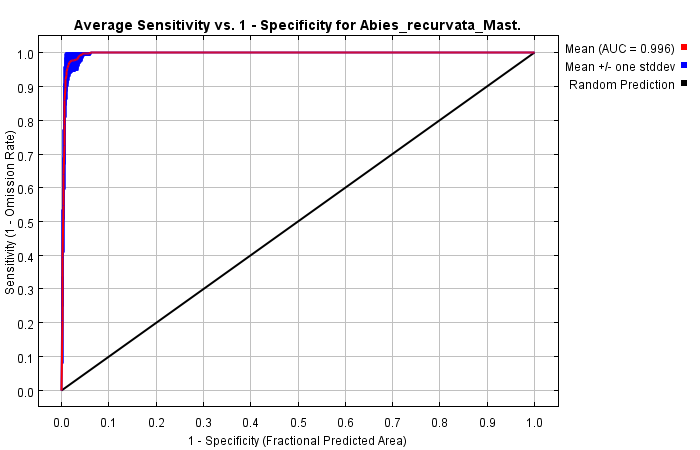
**

**Figure S2**

**
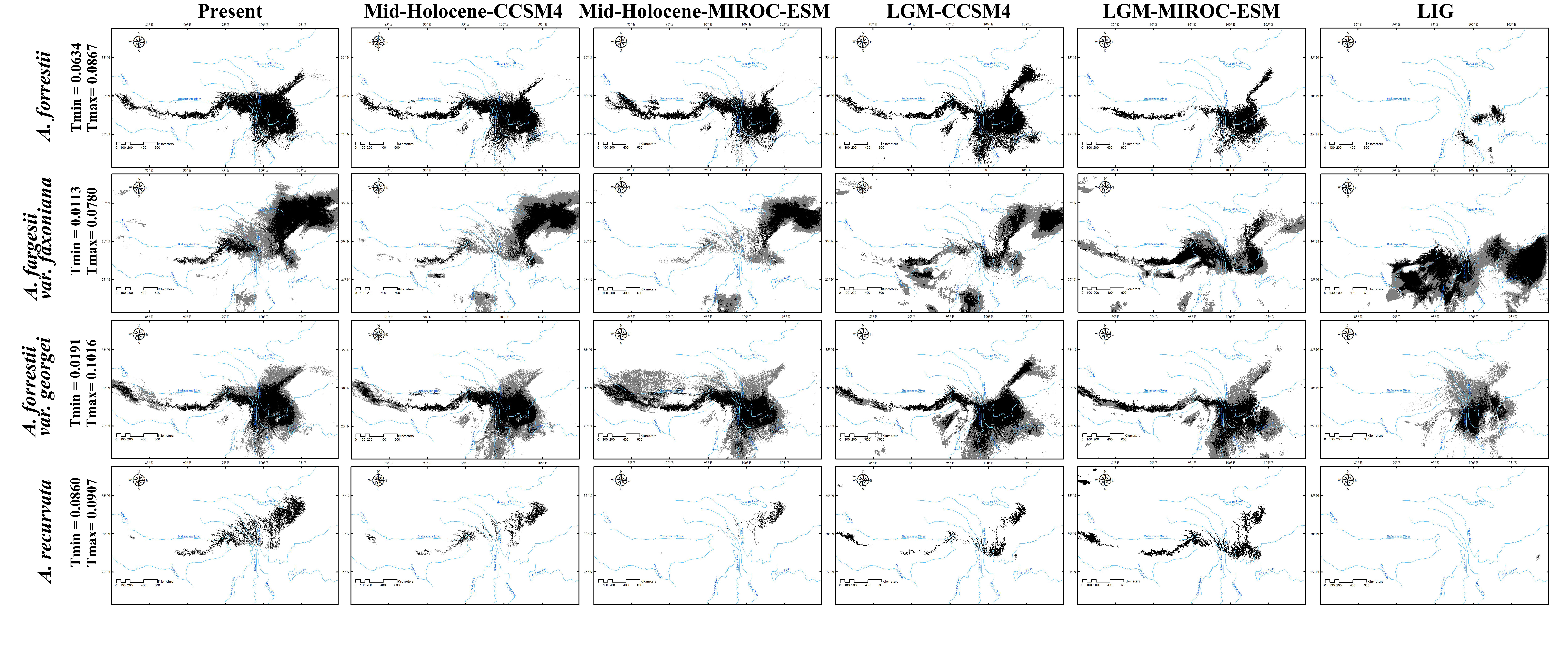
**

**Figure S3**

Mid-Holocene-CCSM4


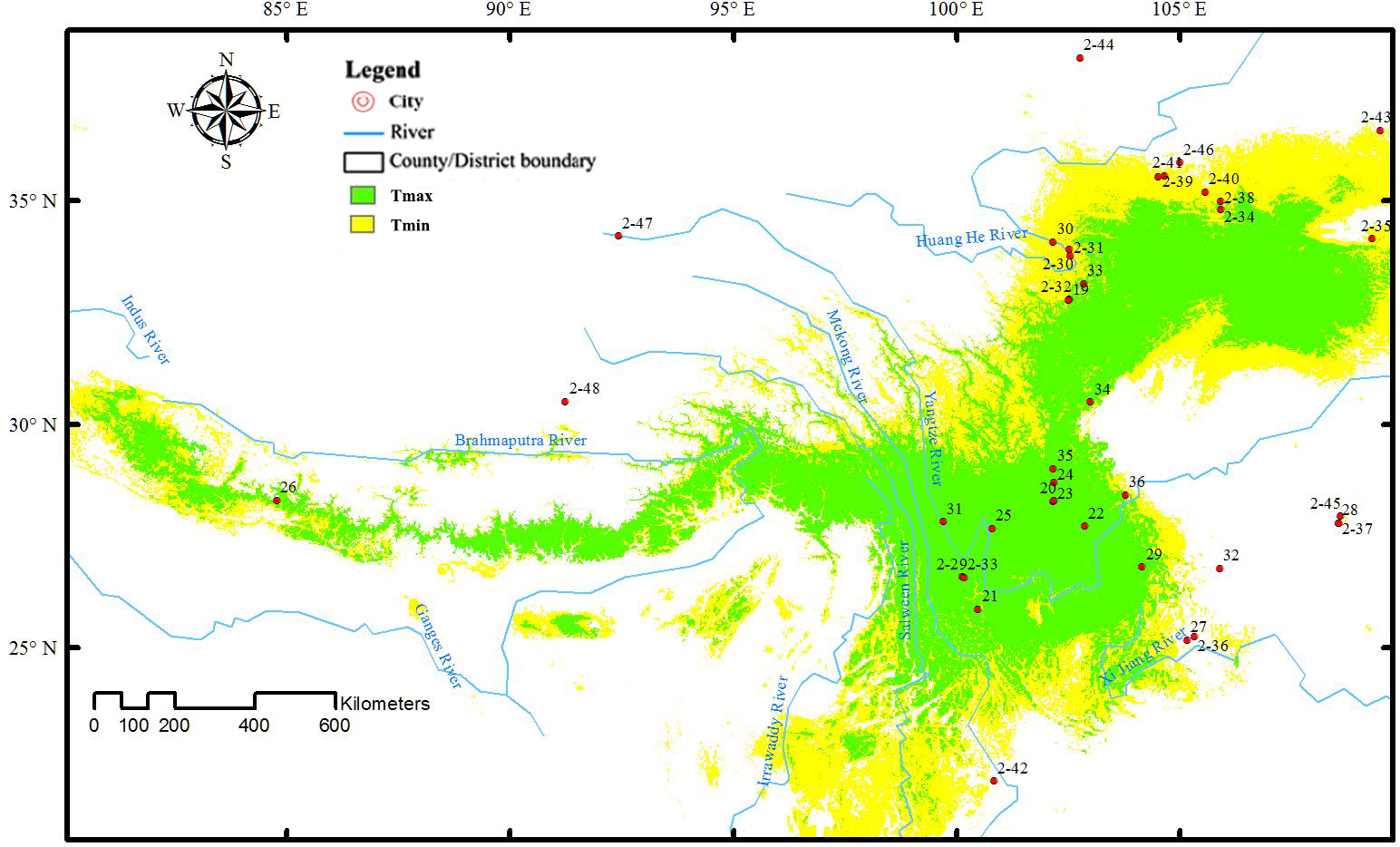


Mid-Holocene-MIROC-ESM

**
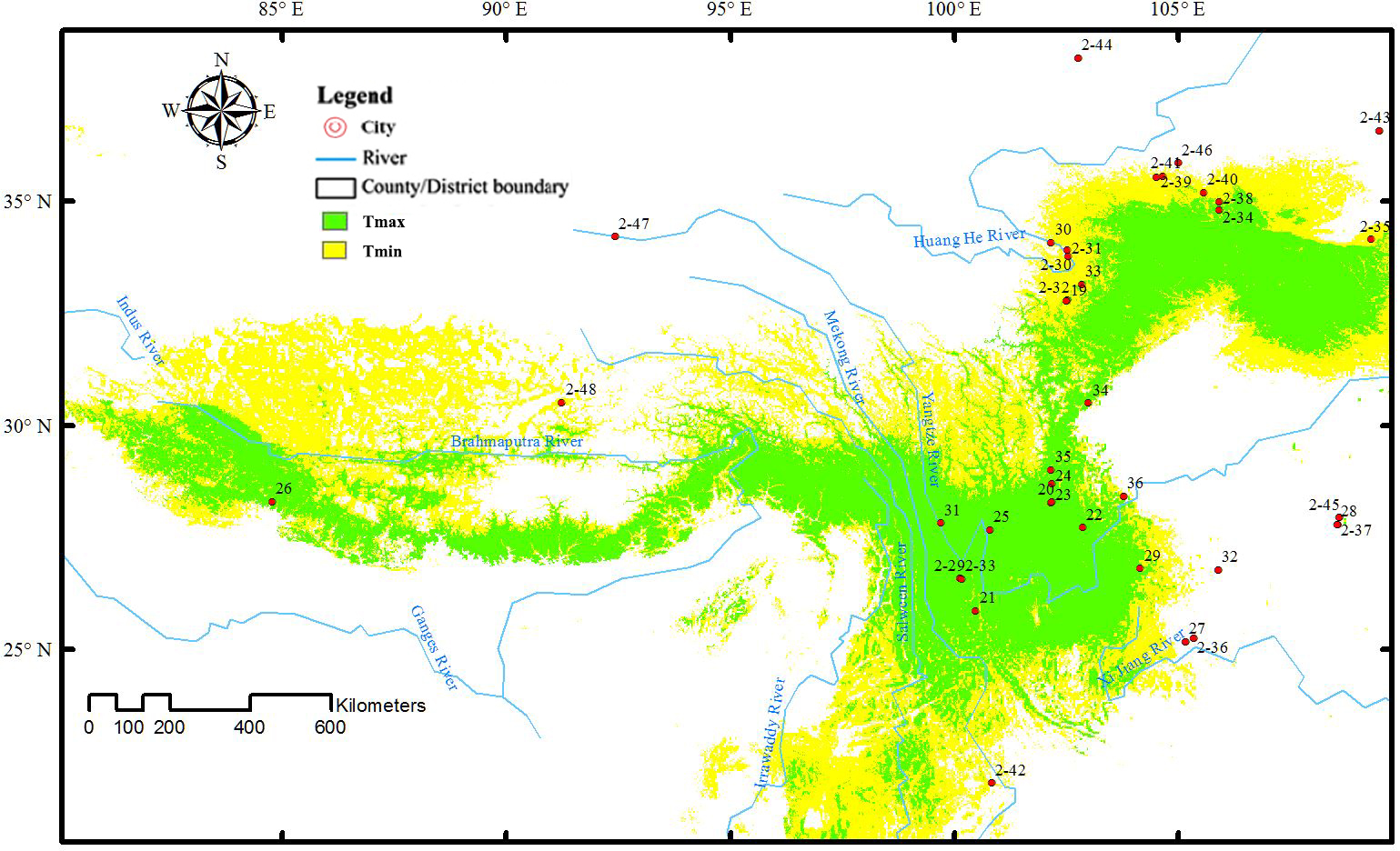
**

LGM-CCSM4

**
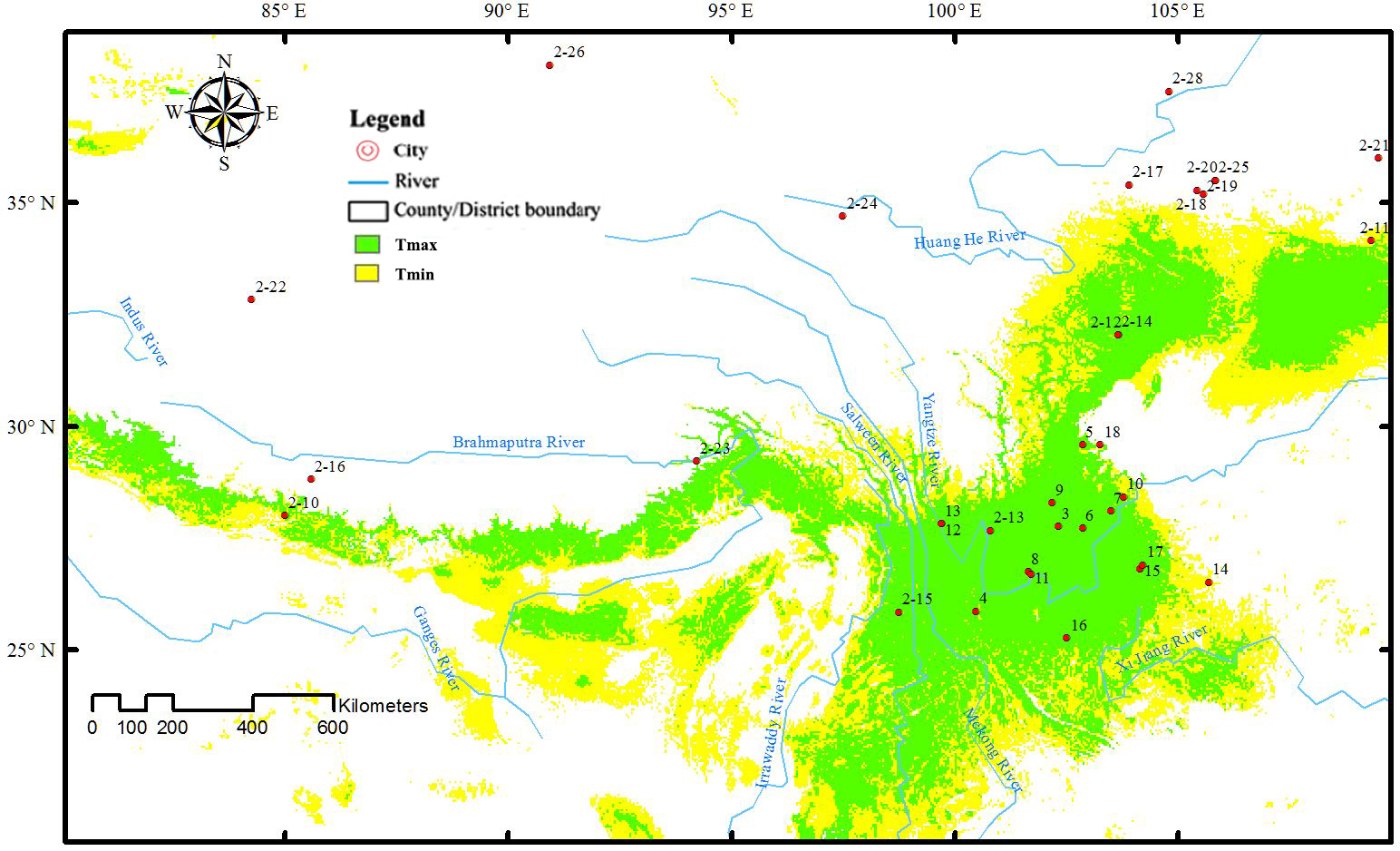
**

LGM-MIROC-ESM

**
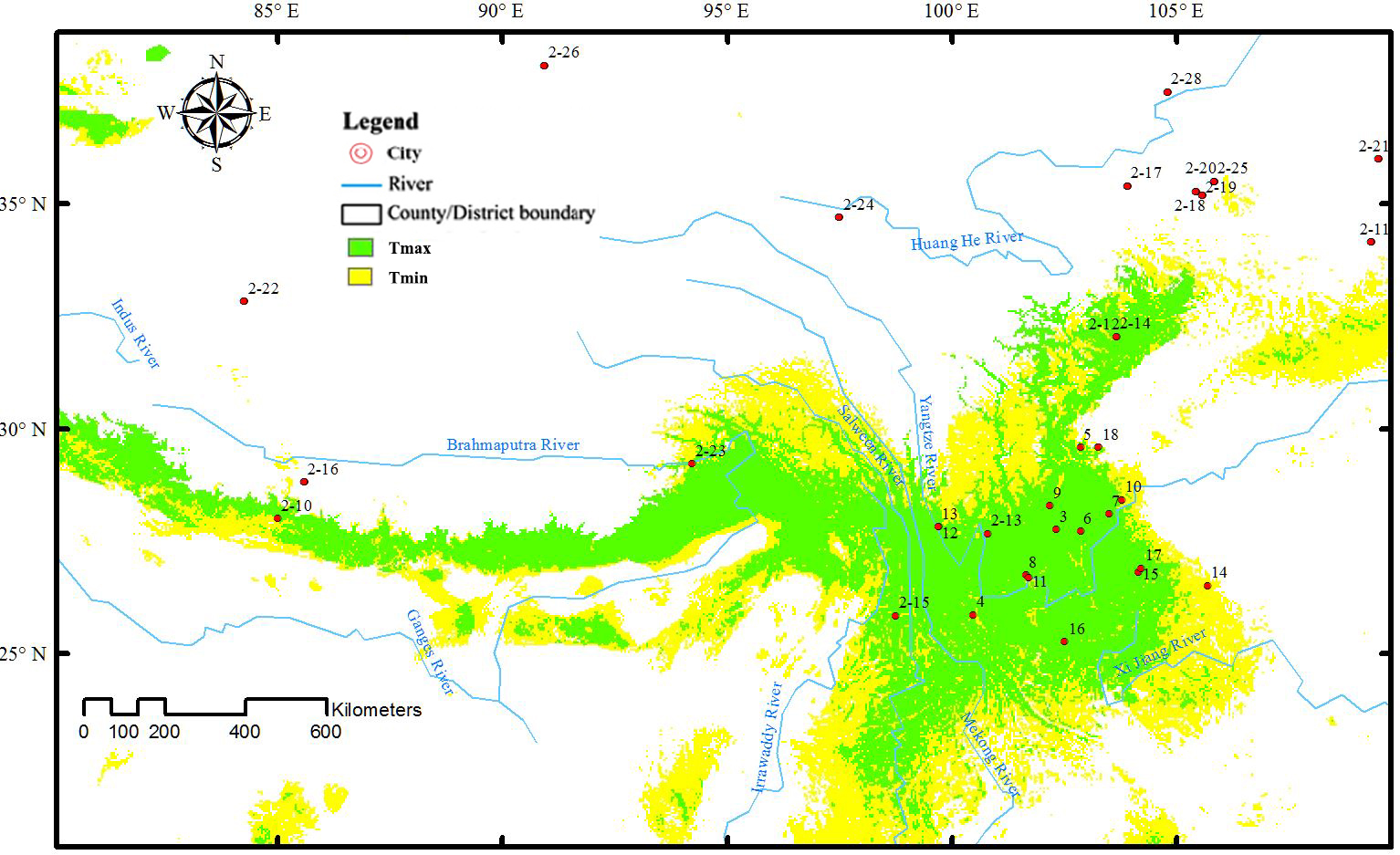
**

LIG


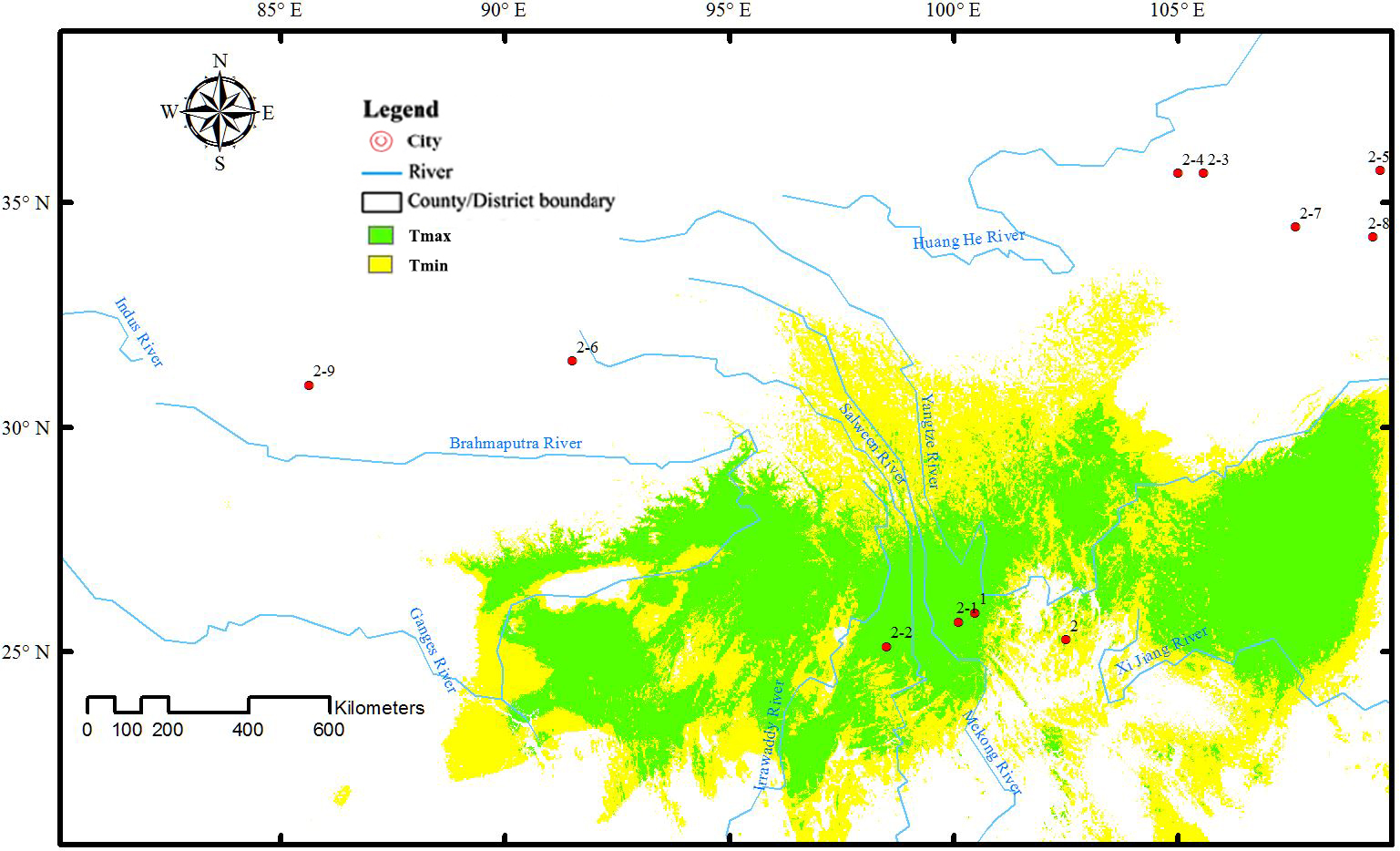


**Figure S4**

Mid-Holocene-CCSM4


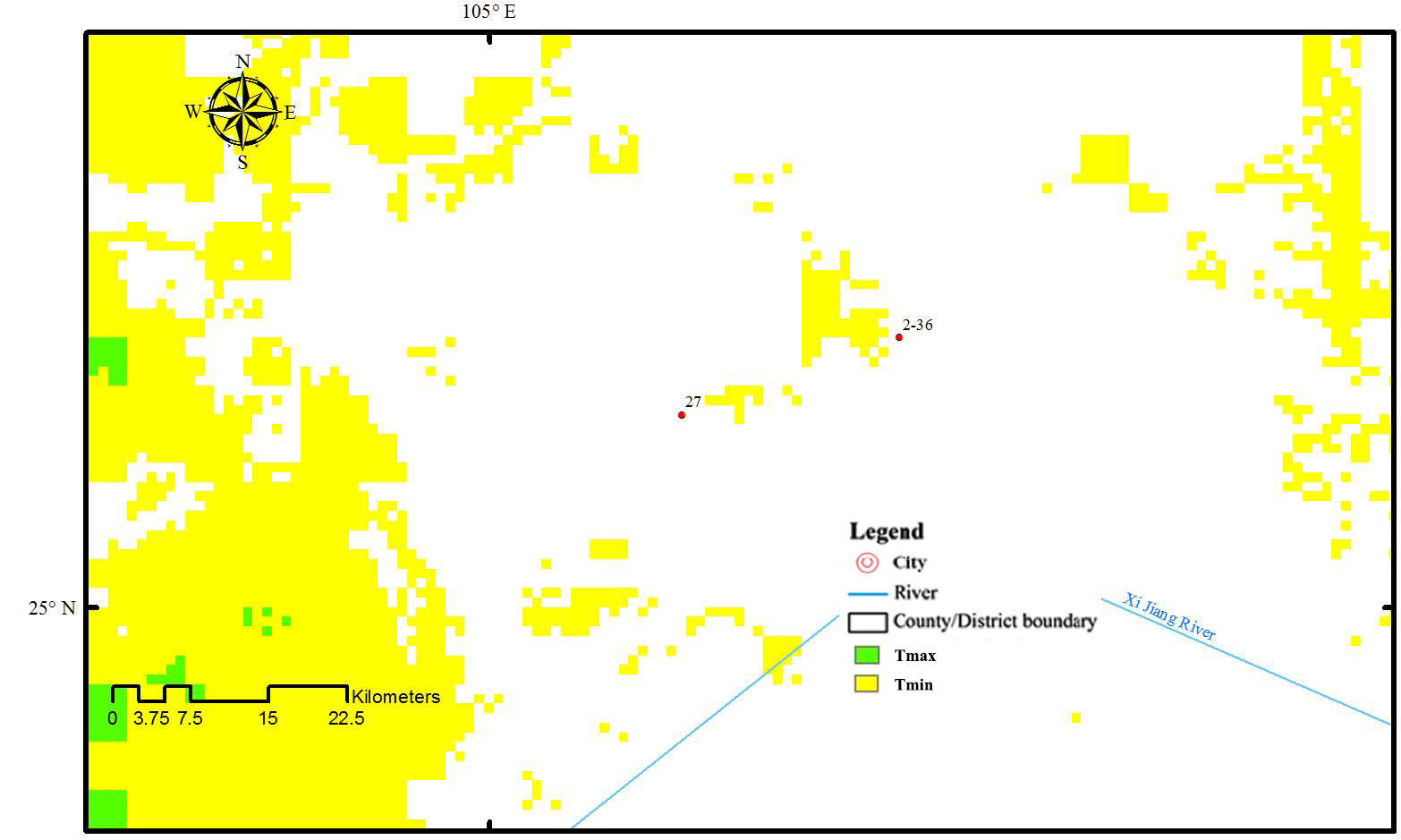

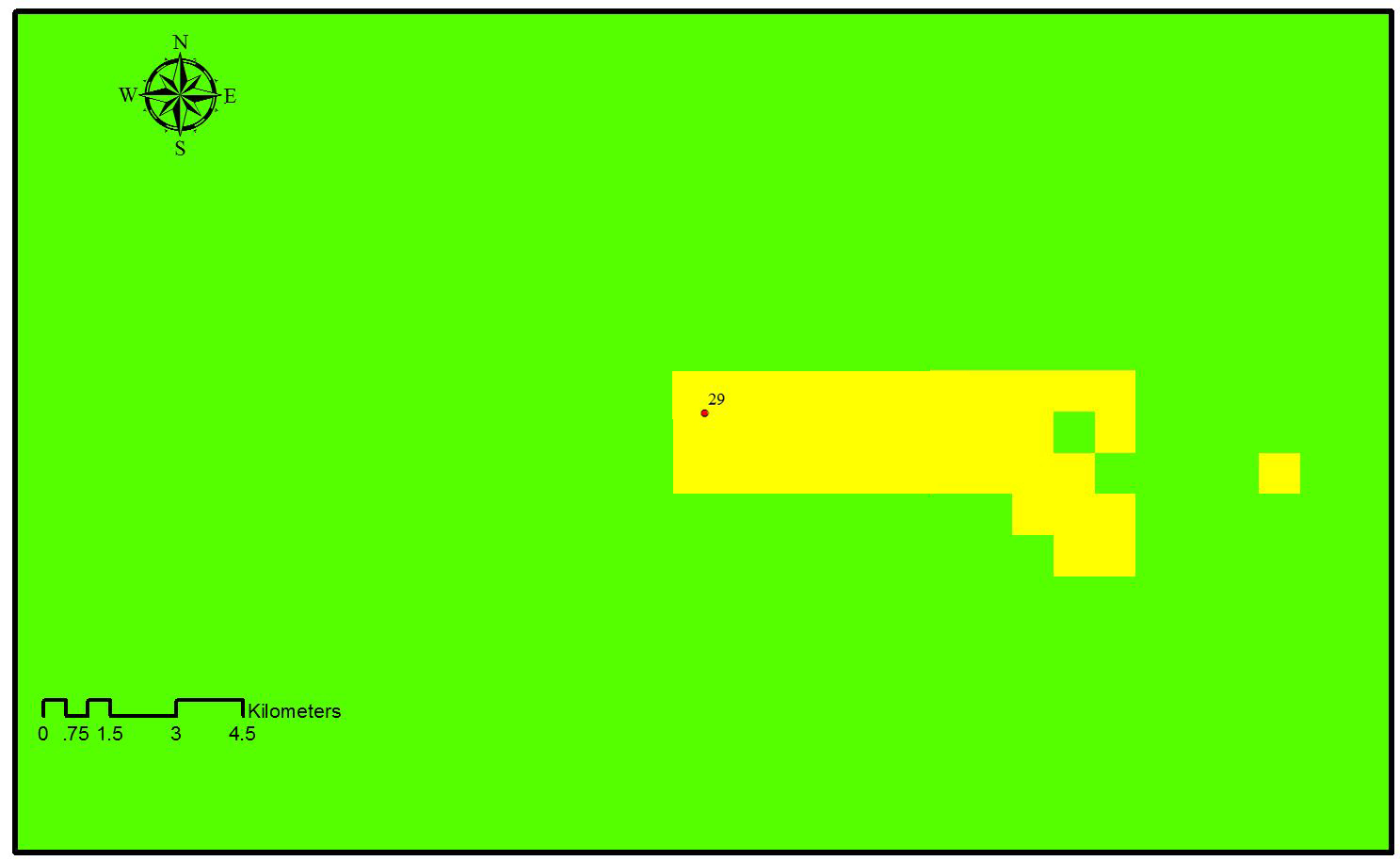


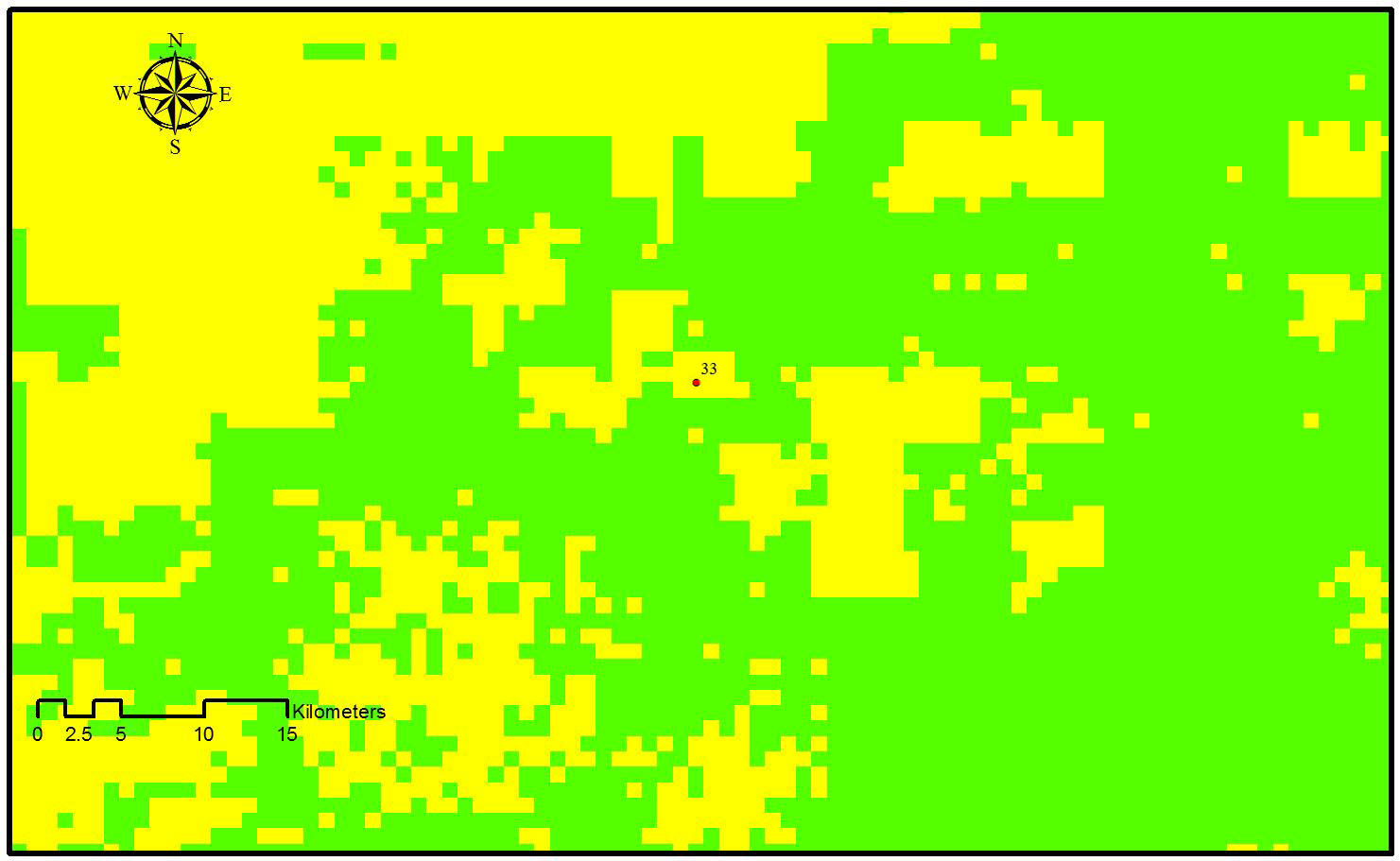

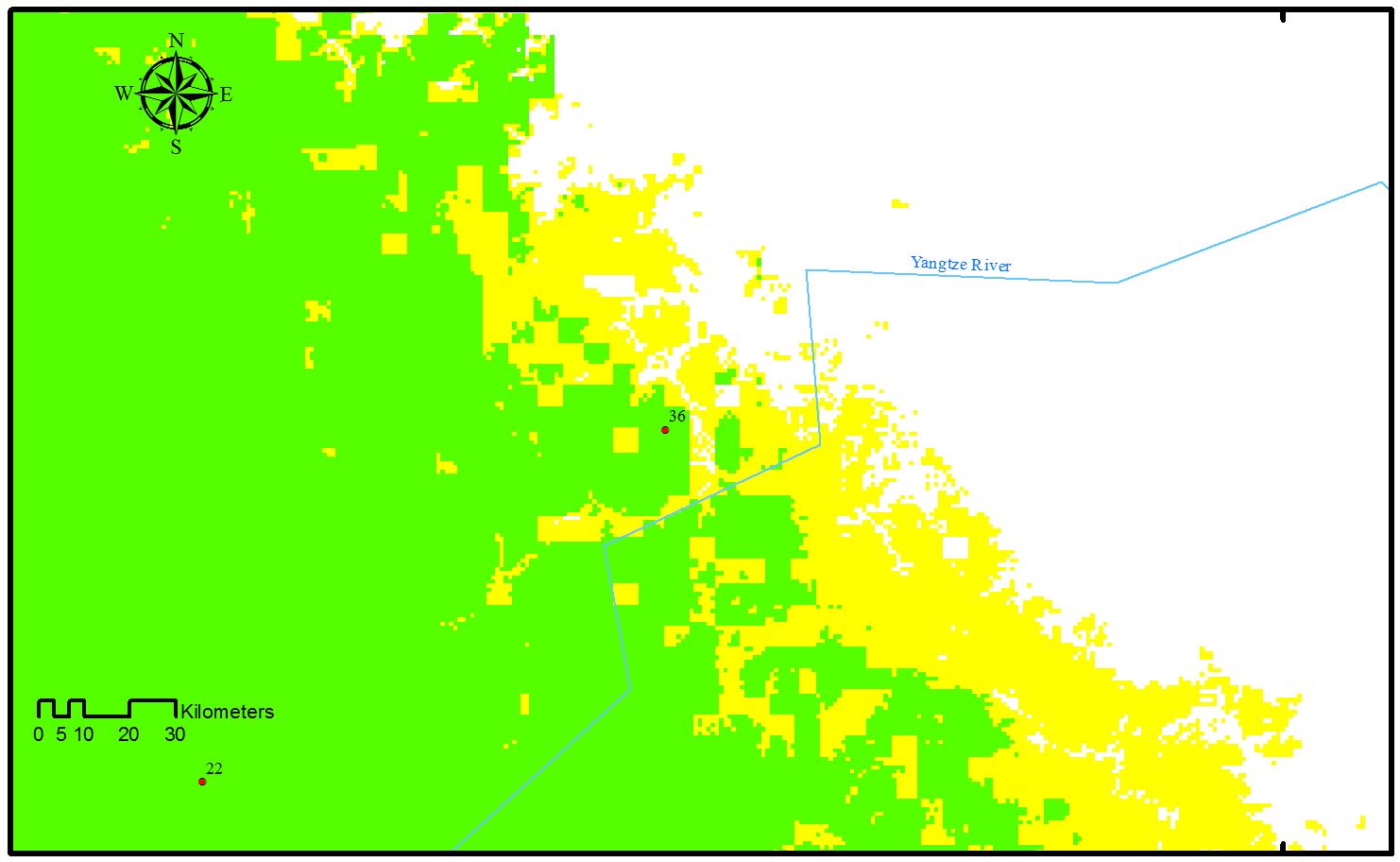


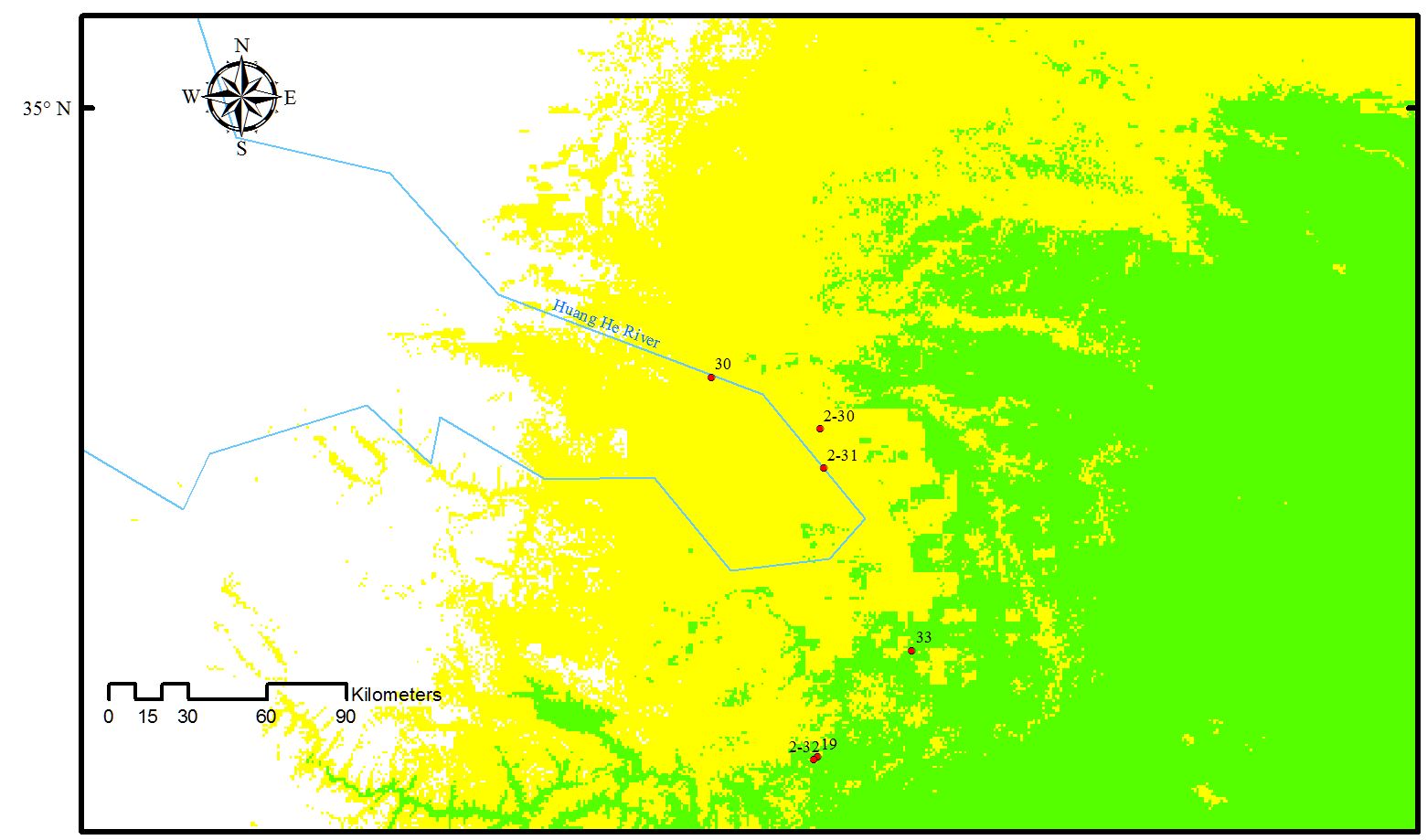

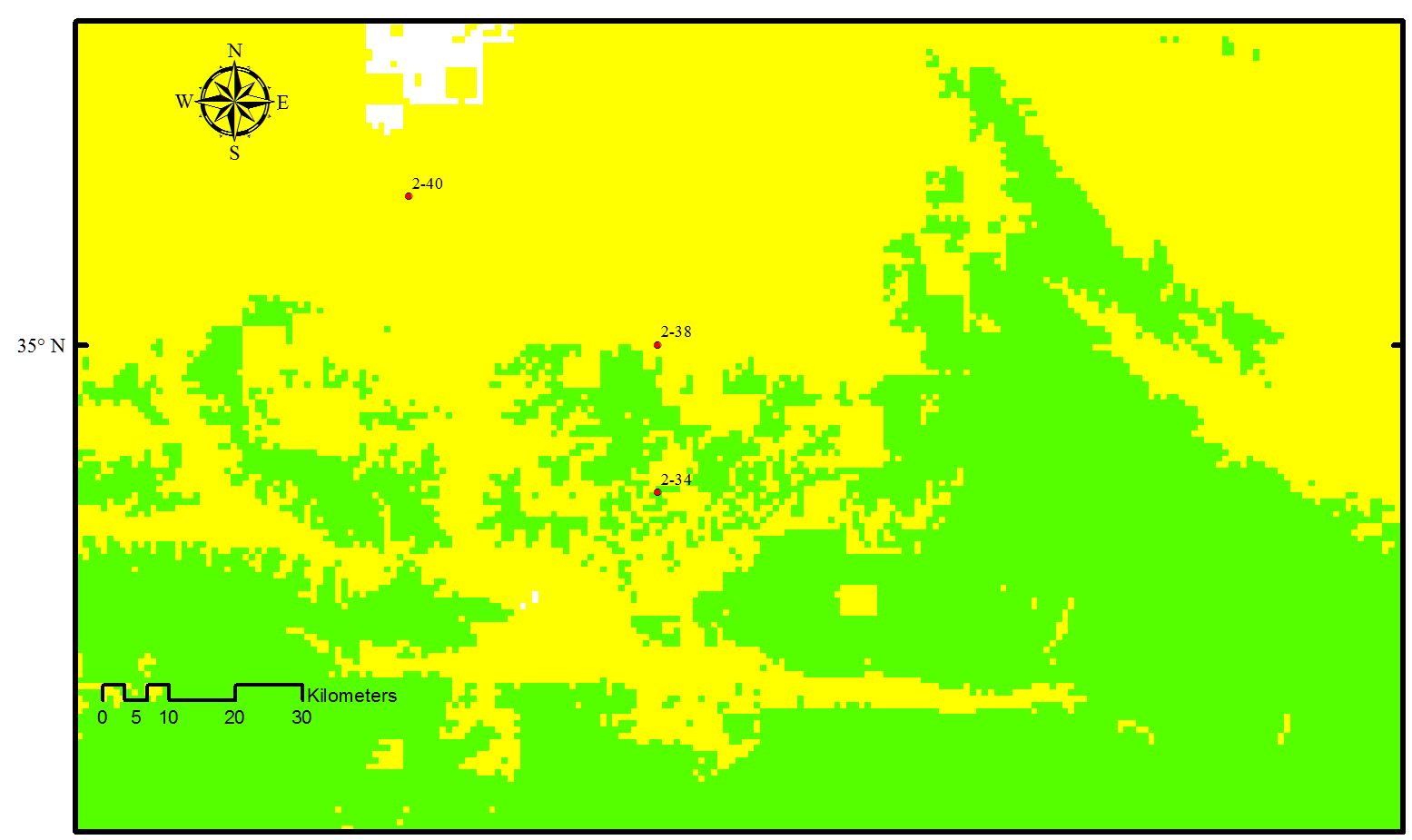


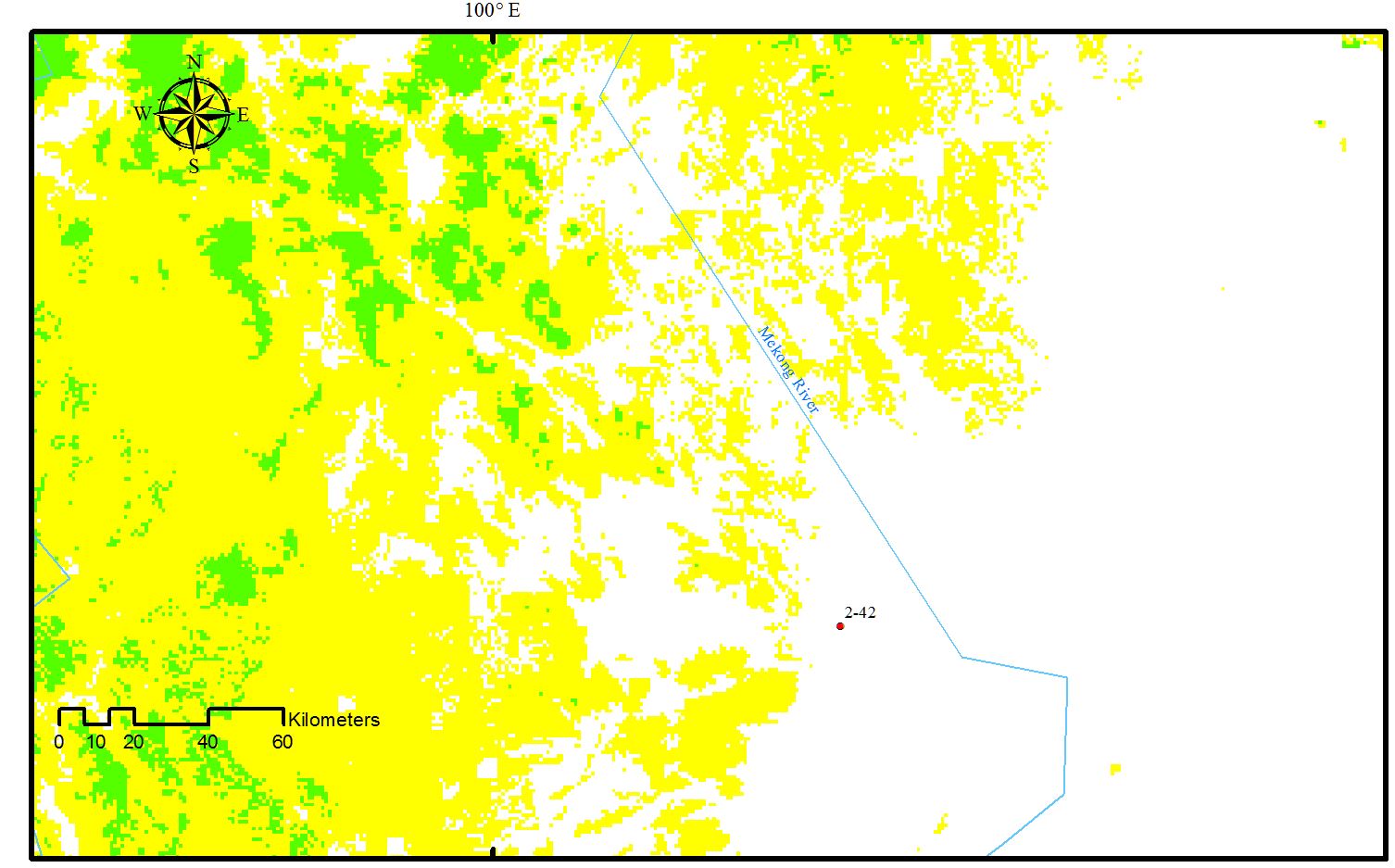

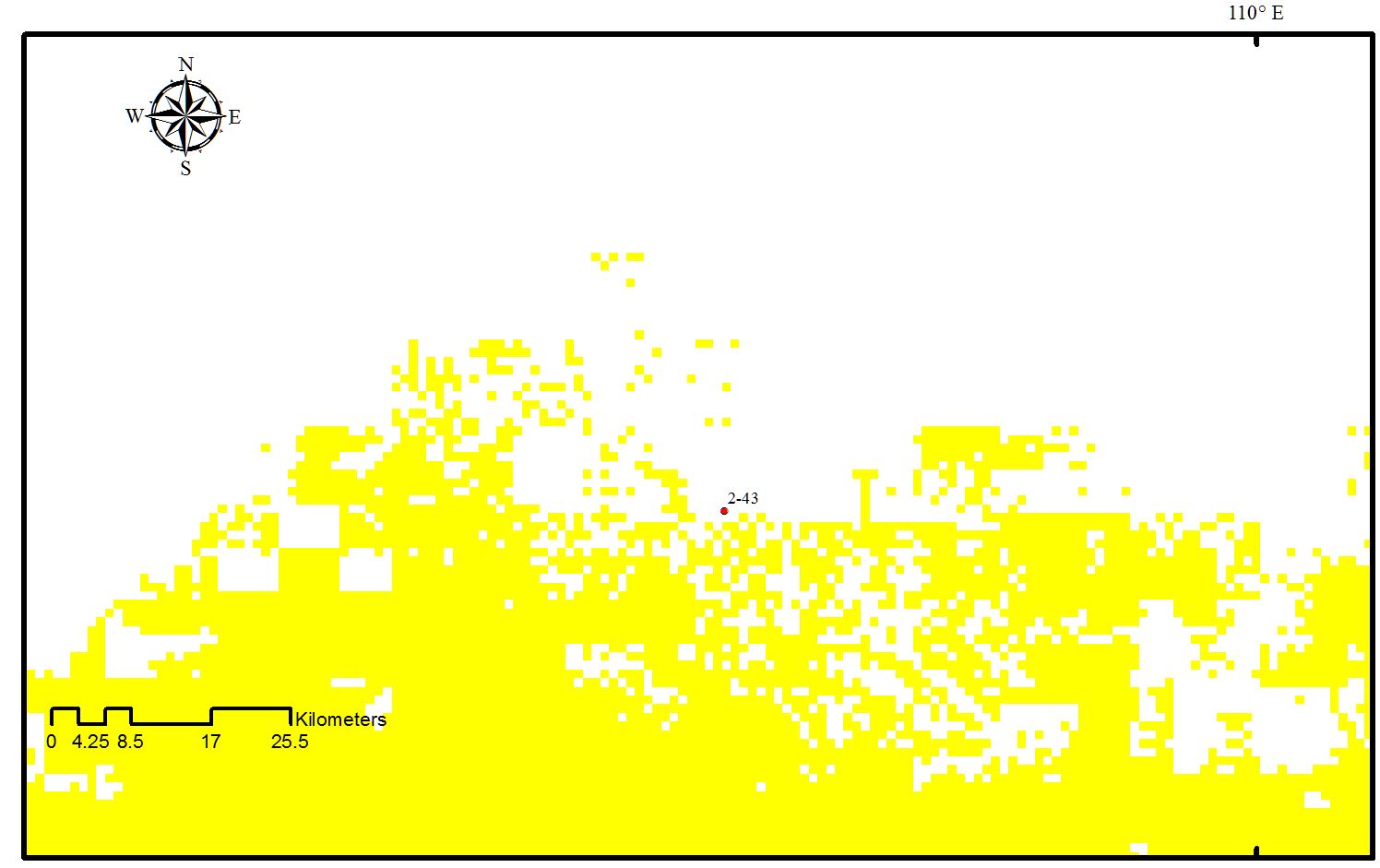


Mid-Holocene-MIROC-ESM


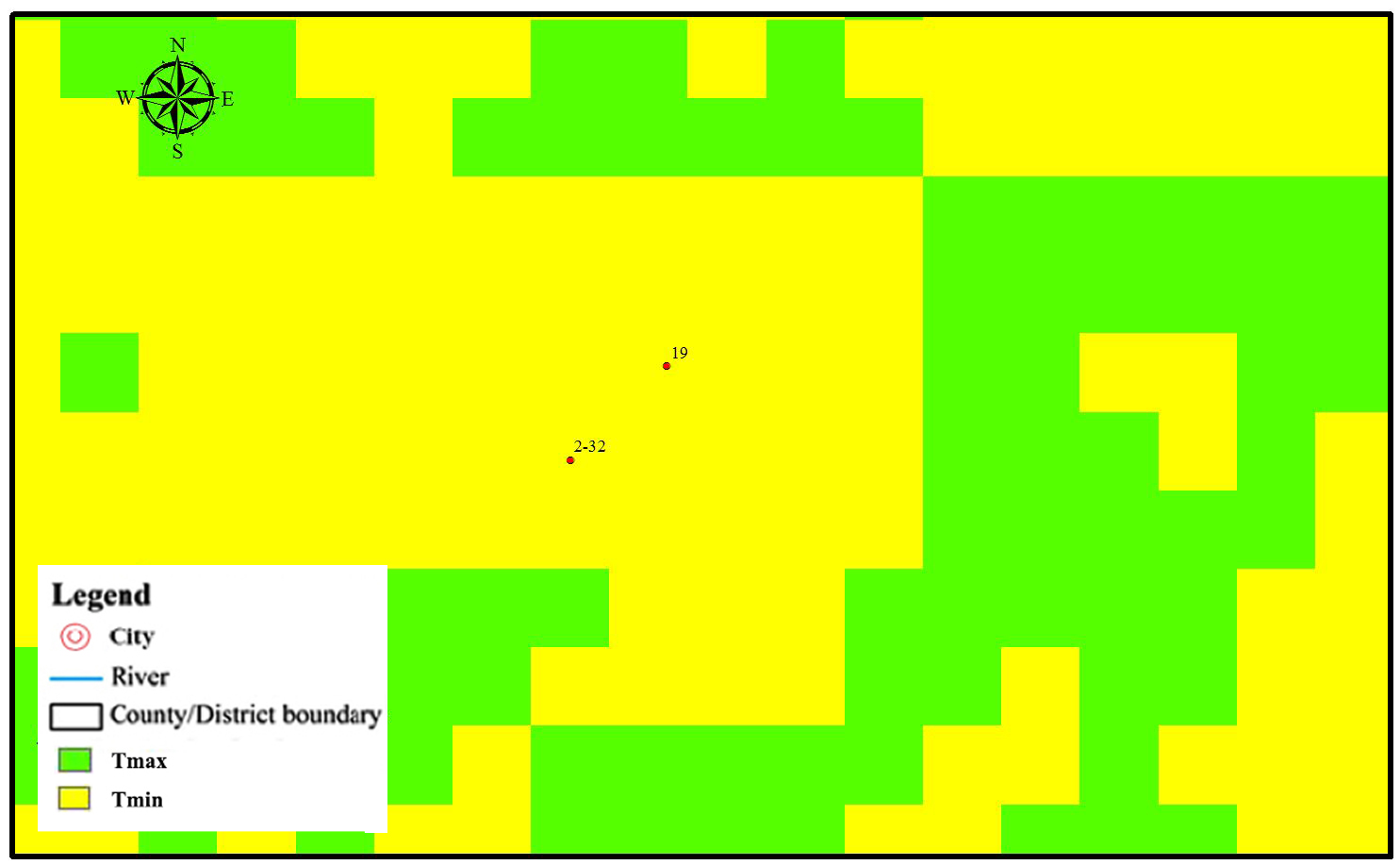

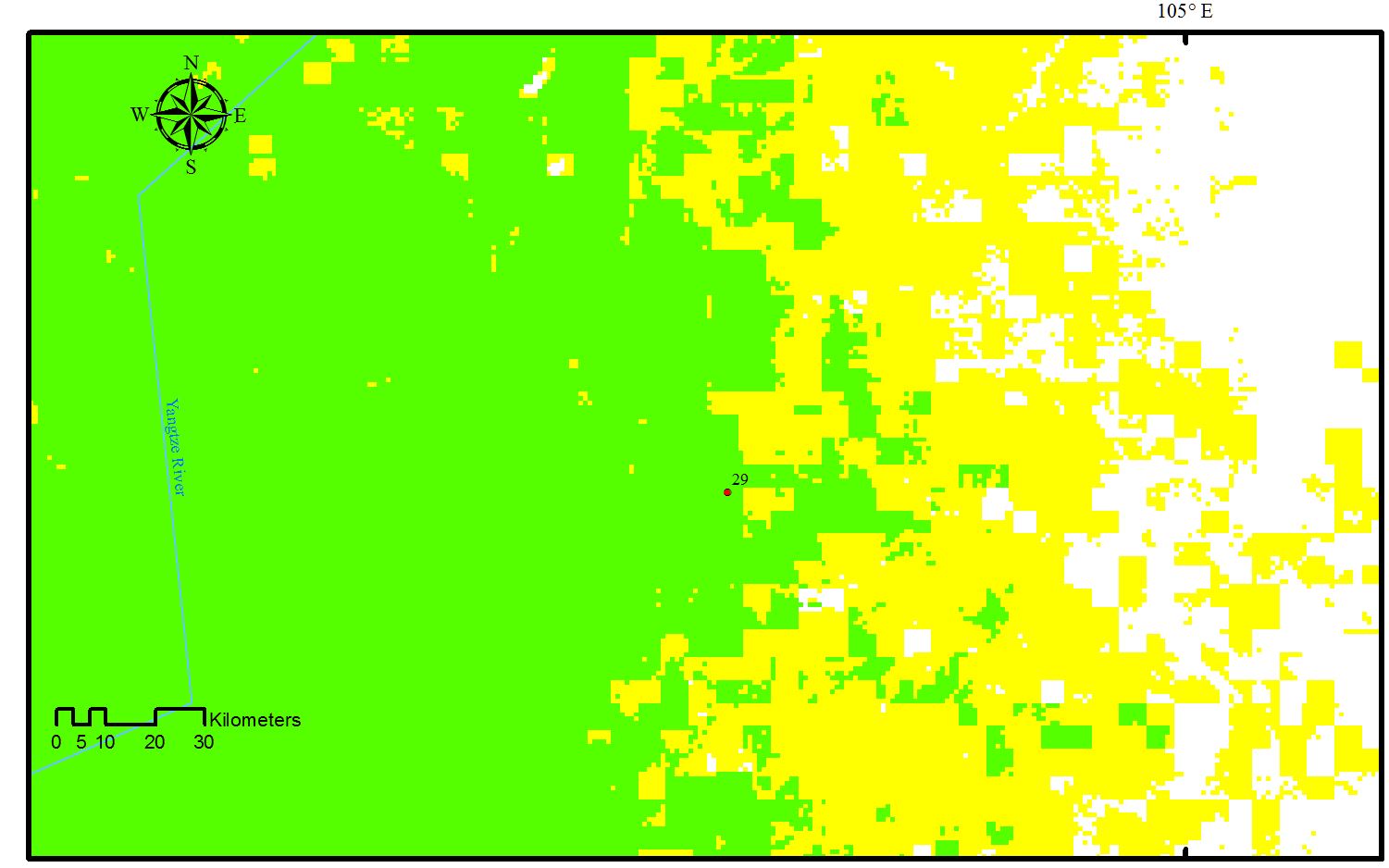


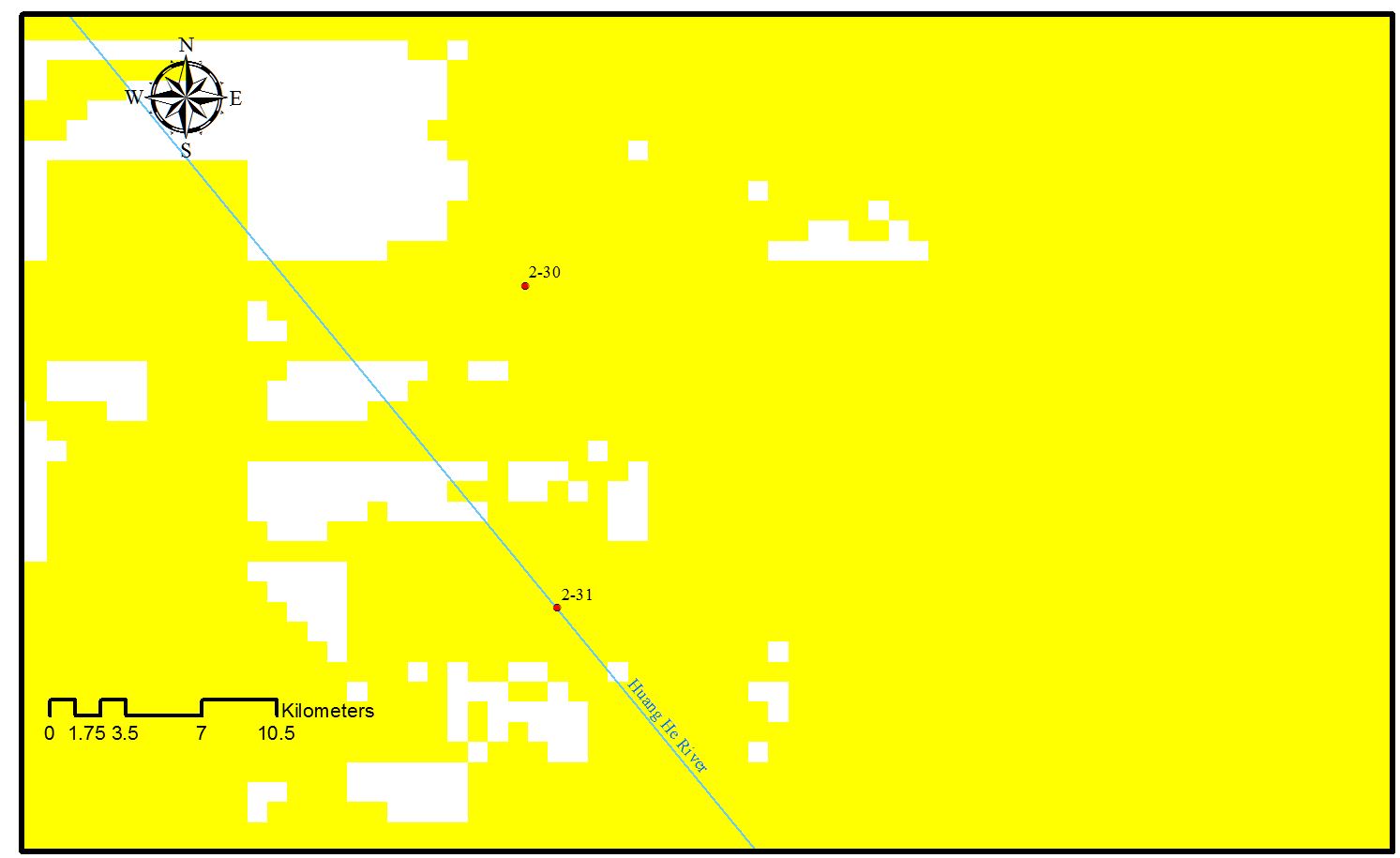

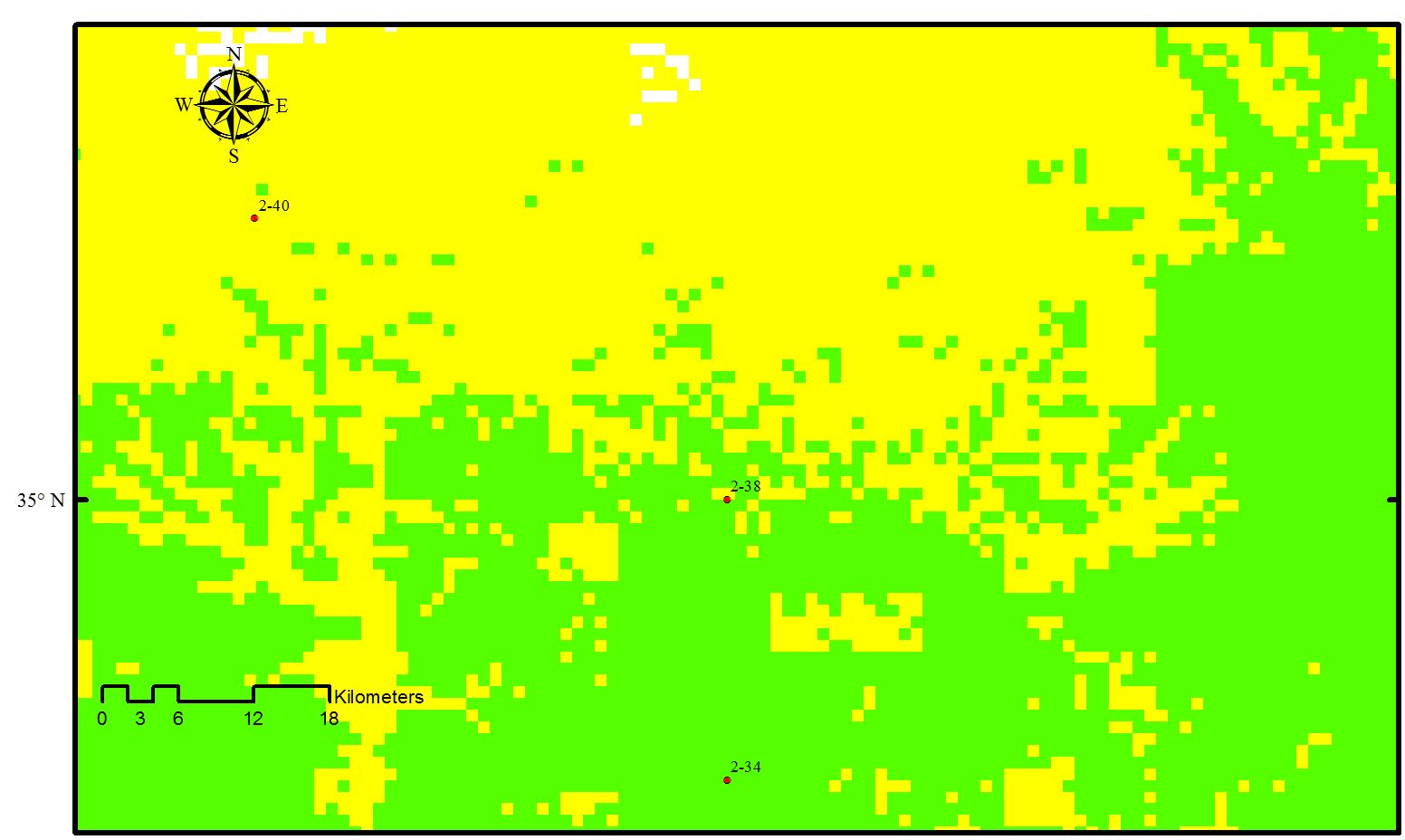


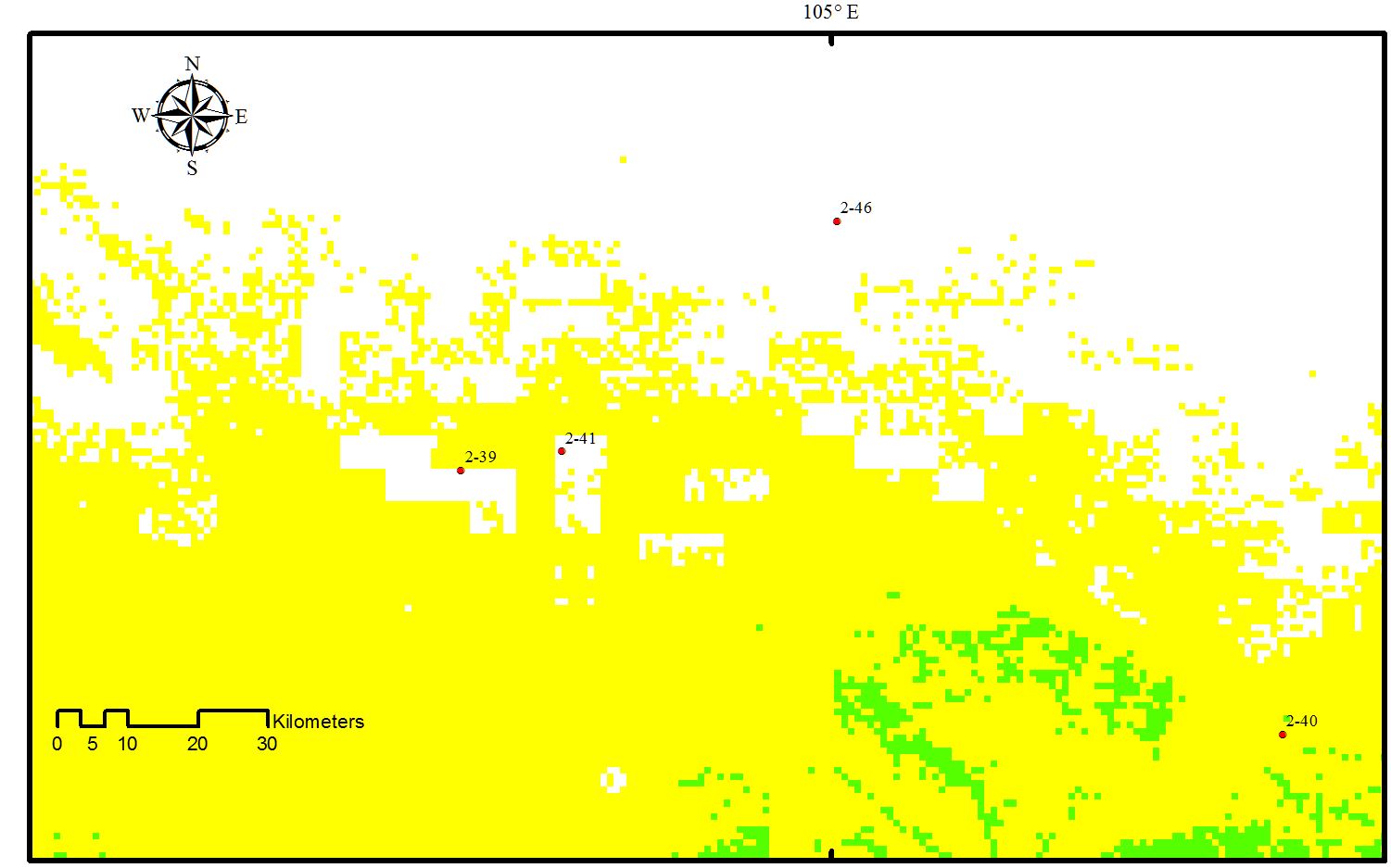

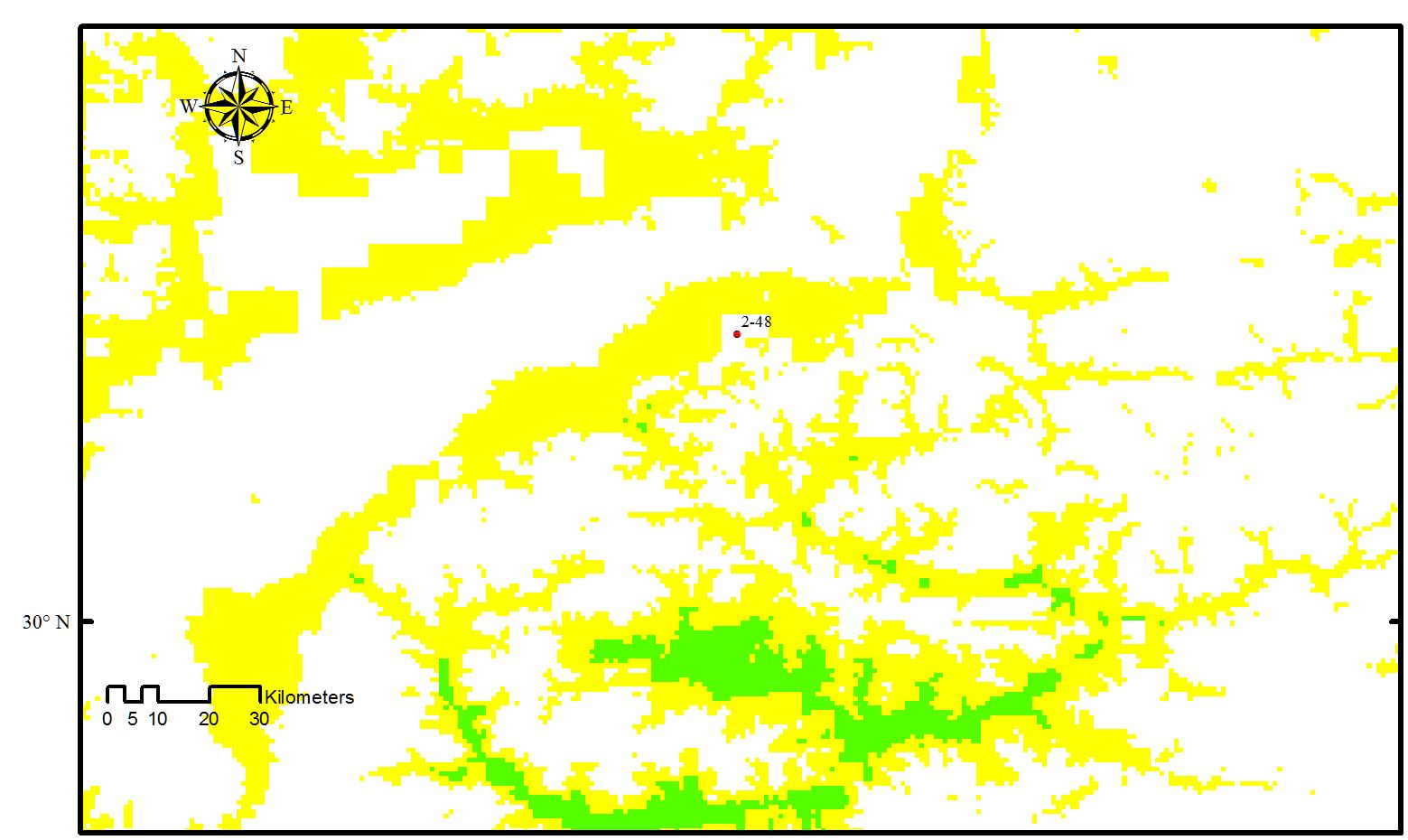


LGM-CCSM4


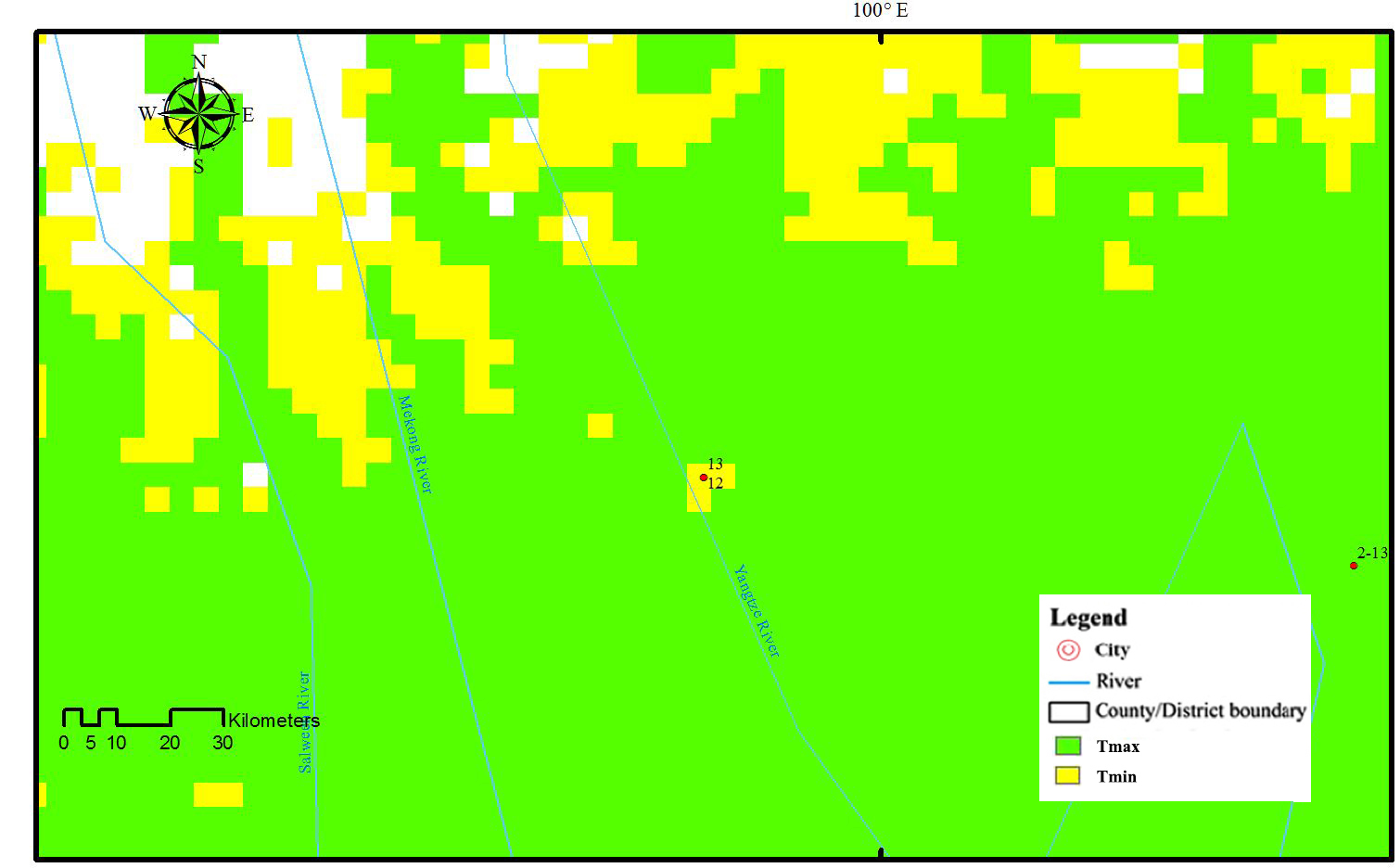

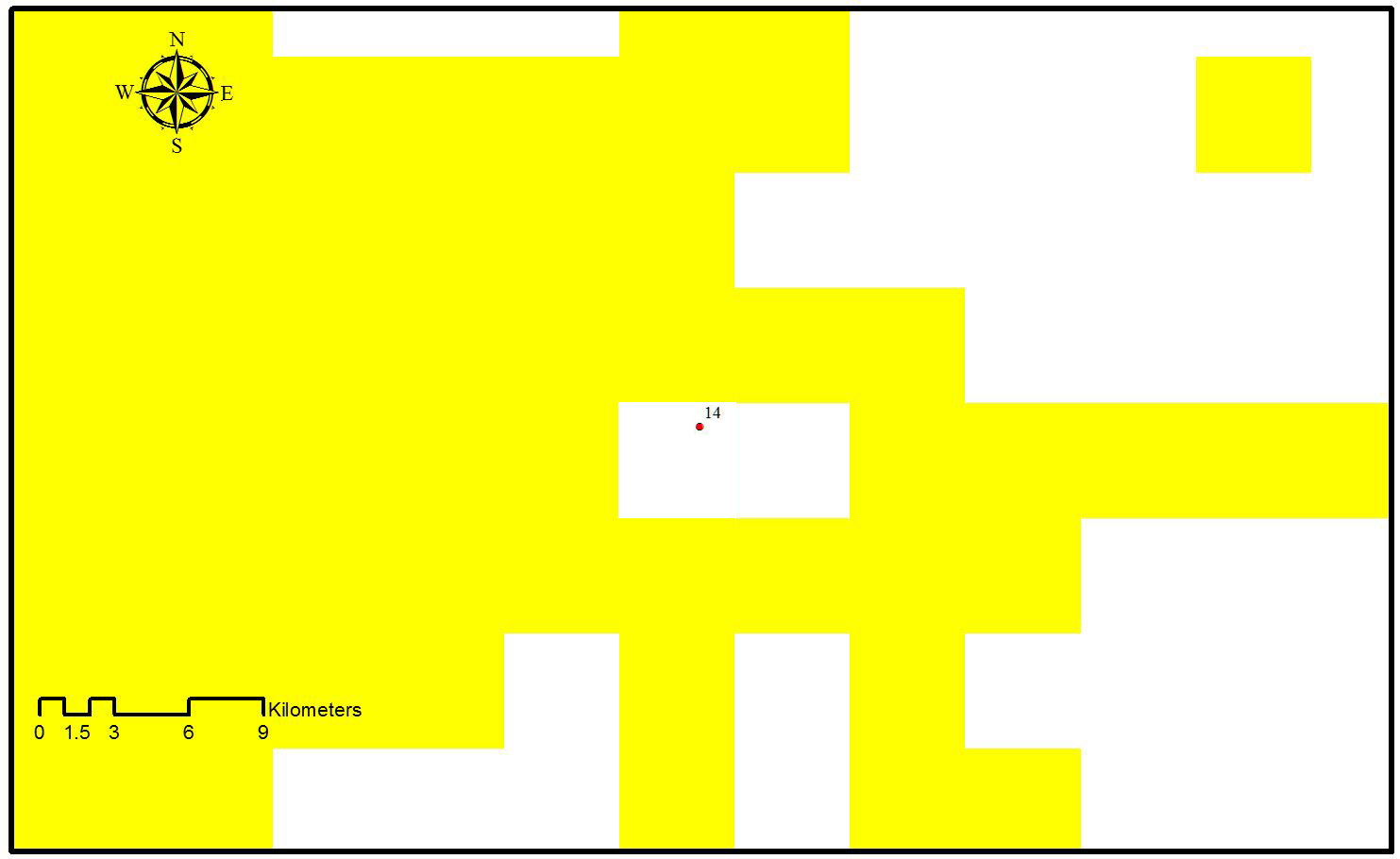


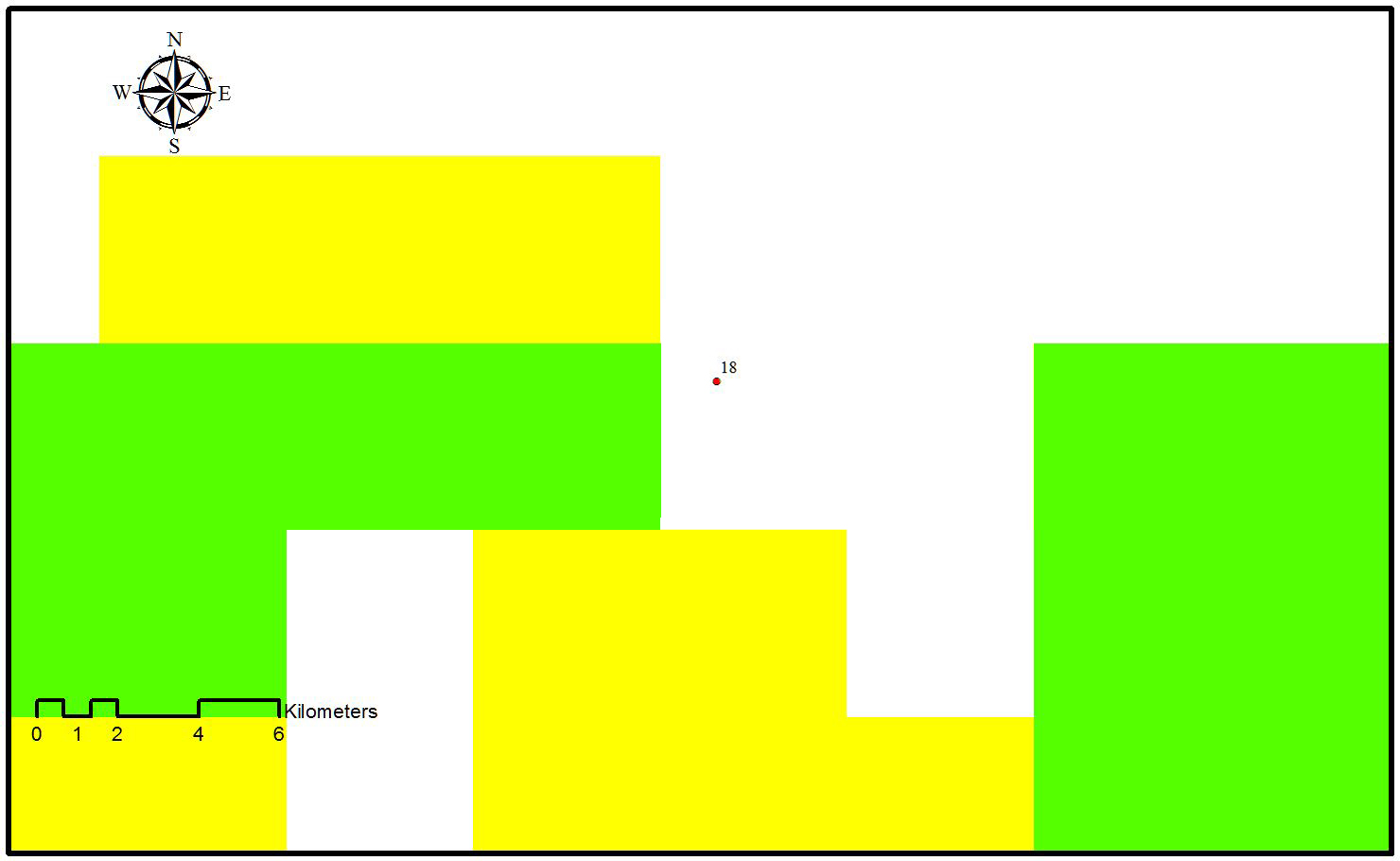

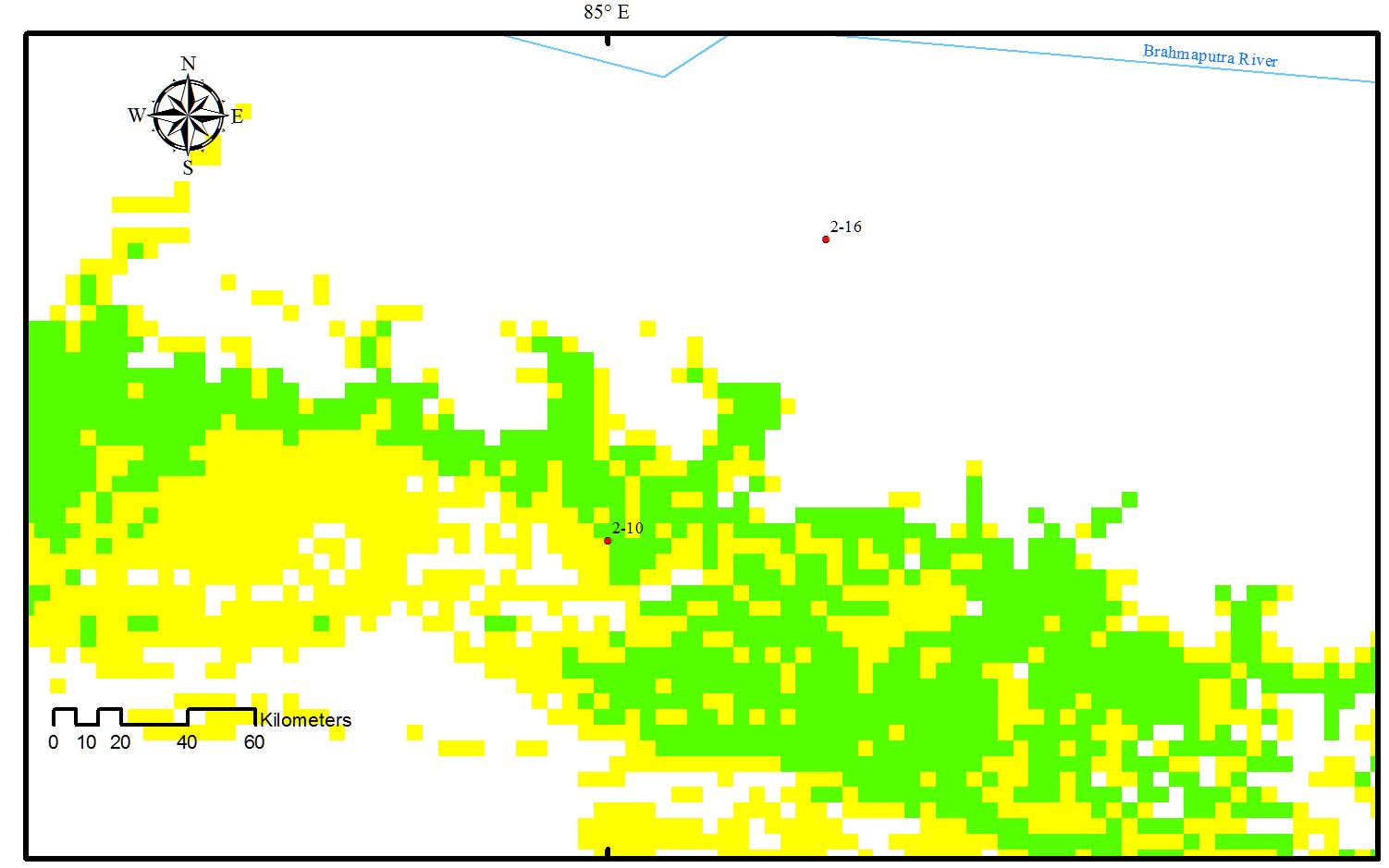


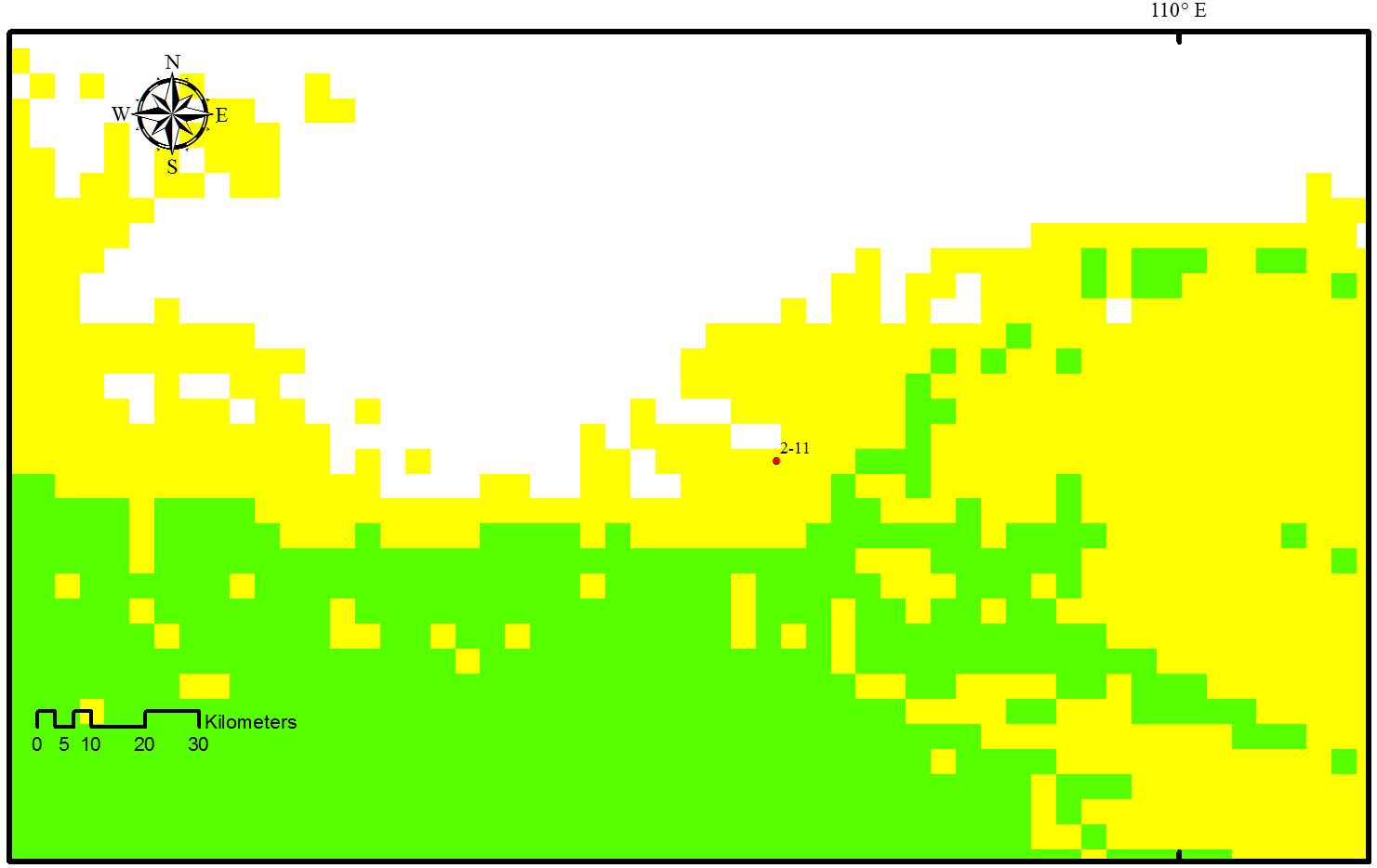

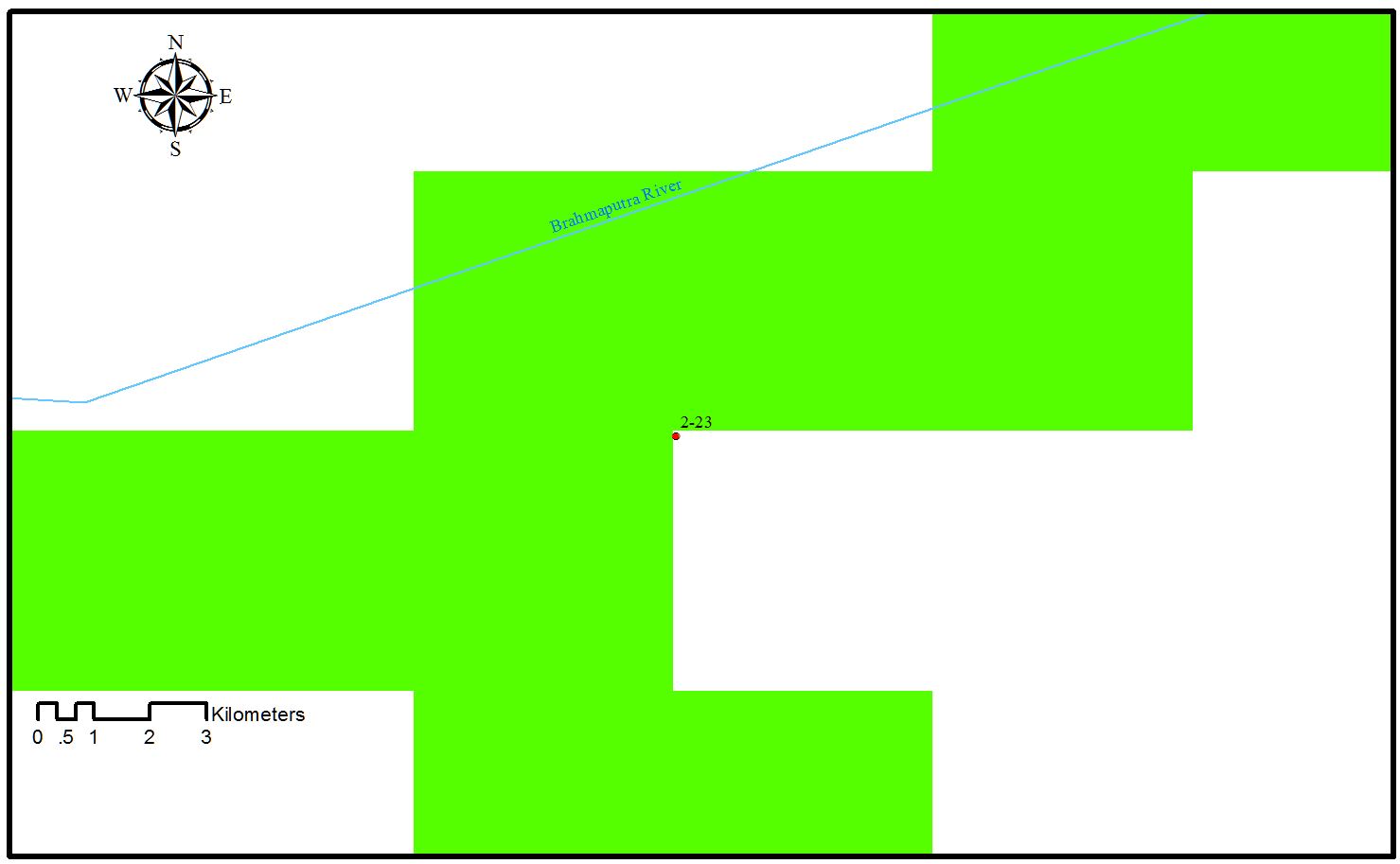


LGM-MIROC-ESM


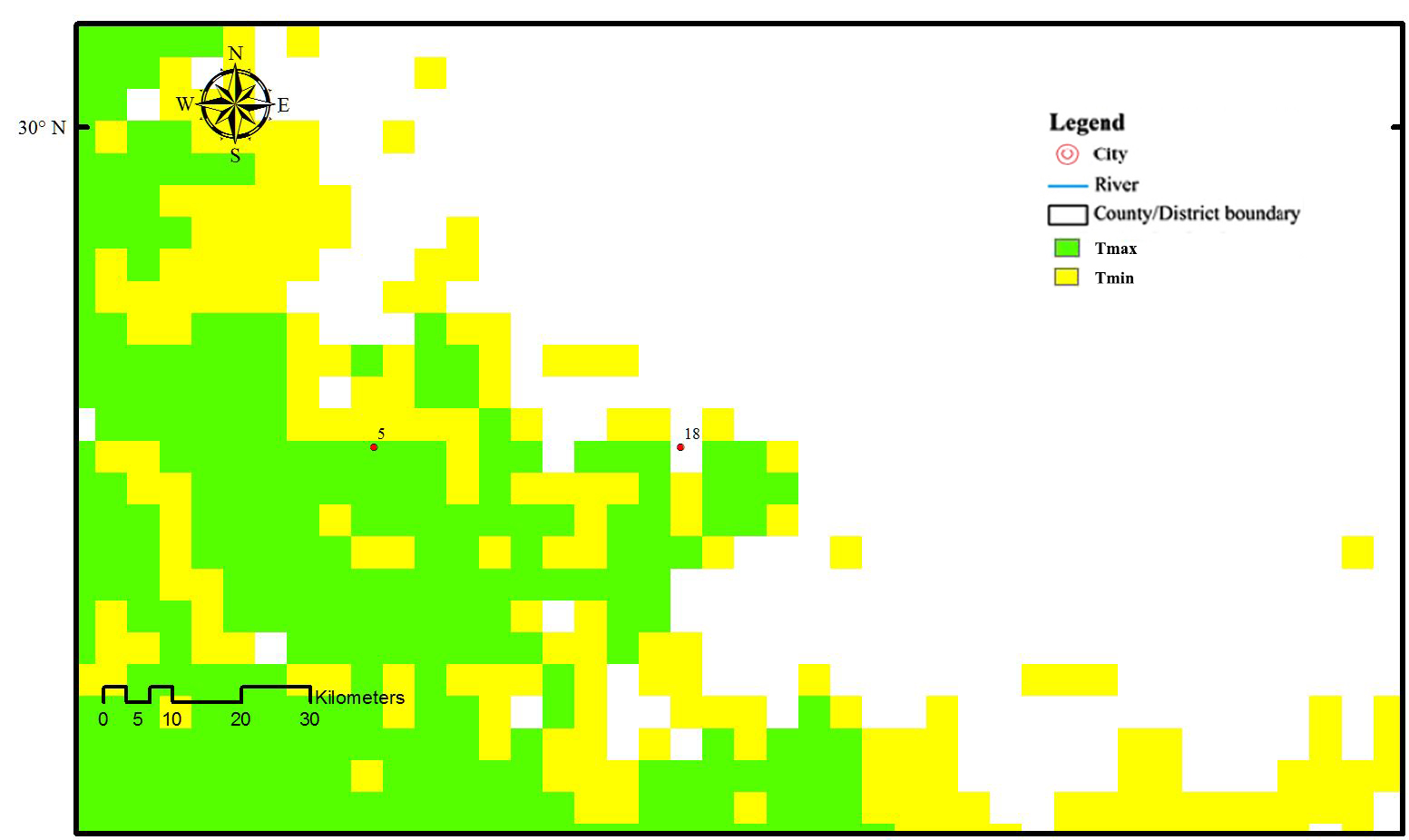

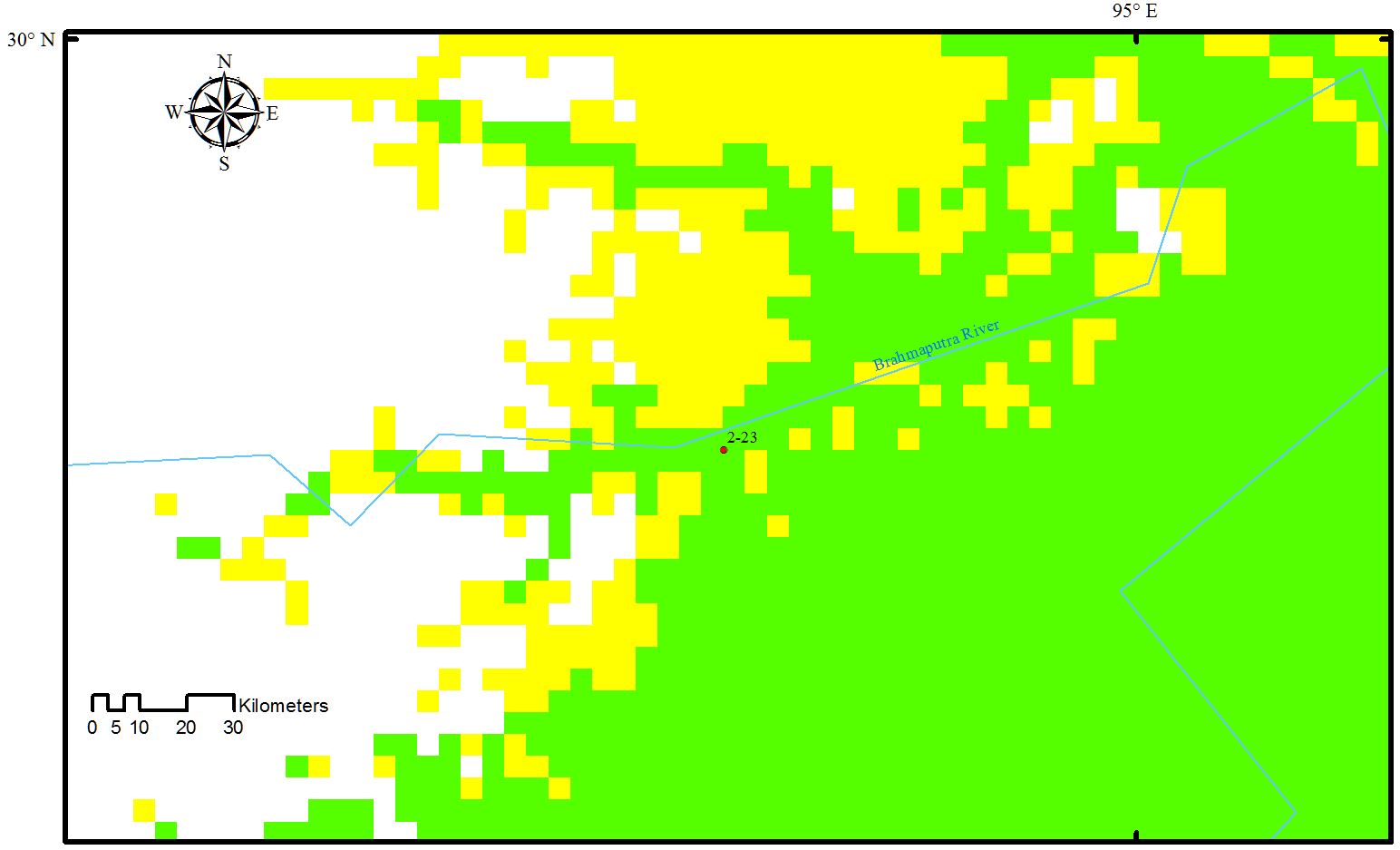


LIG


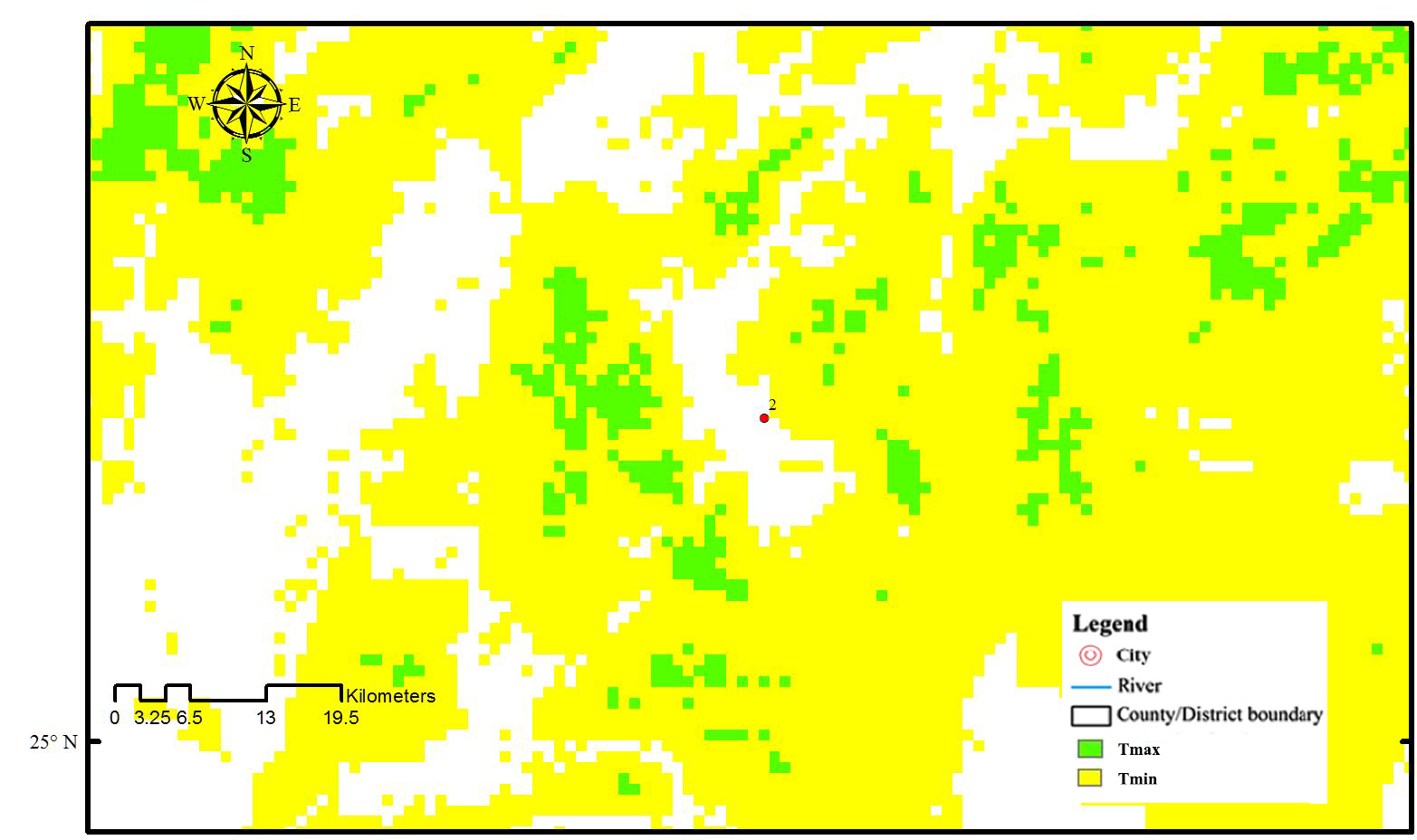


**Figure S5**


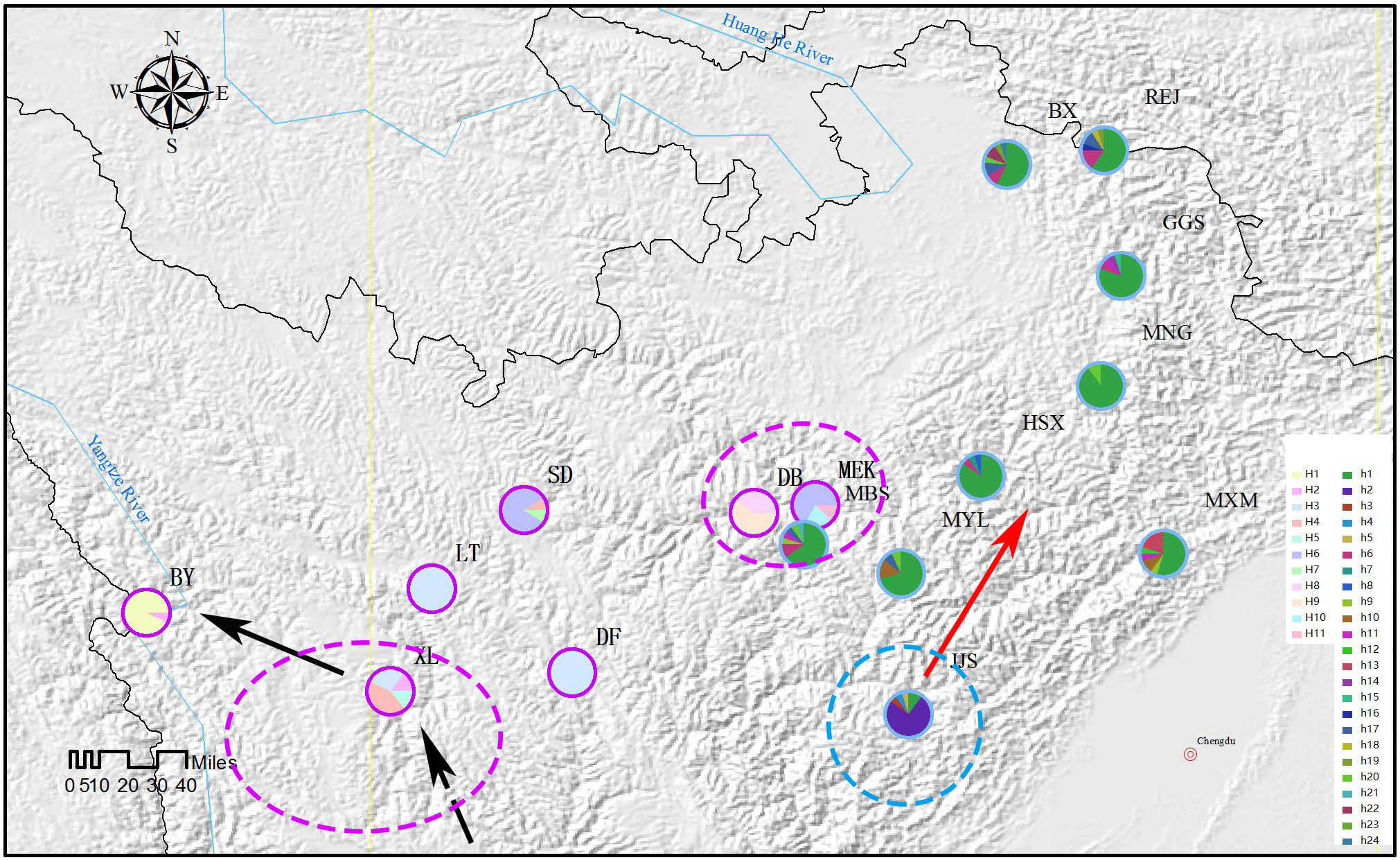


**Longmen refuge**

?

**Figure S6**


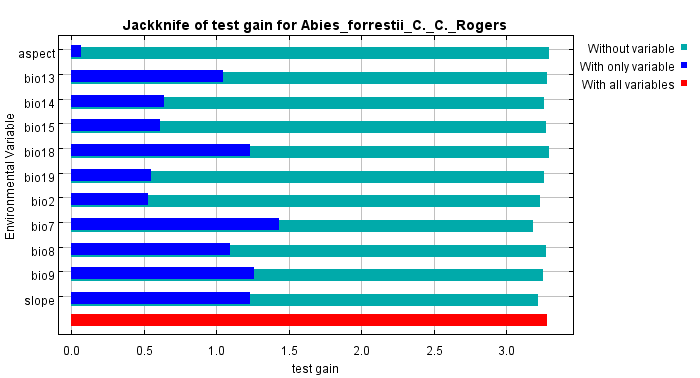

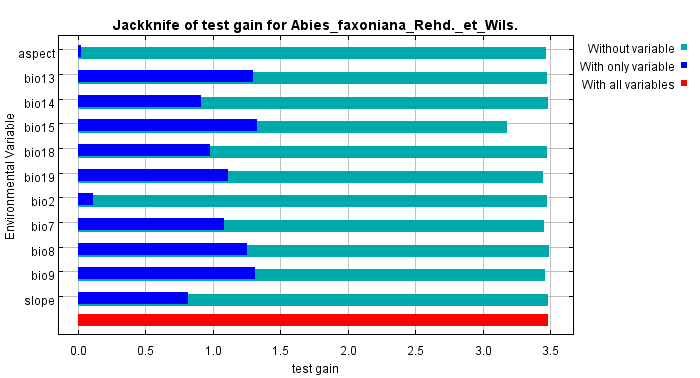


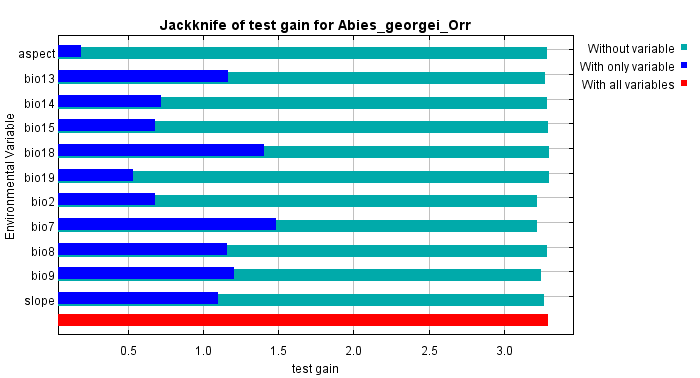


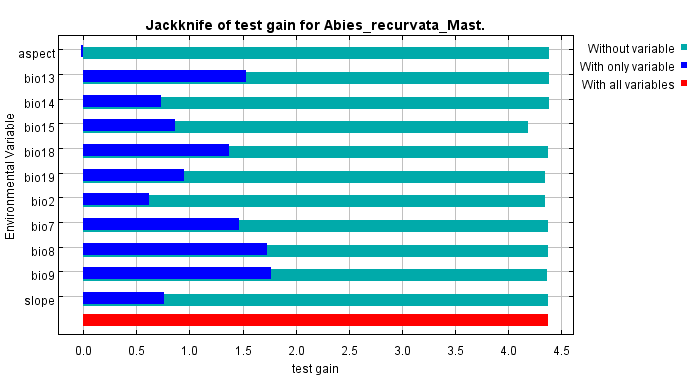


Figure S7

**Table S1** Spearman rank correlation coefficients for 19 bioclimatic variables based on 19 bioclimatic layers on the whole area under current climatic conditions. Green shading indicates uncorrelated variables that were used to model the *Abies* taxa distributions. Spearman p≥0.75 values are highlighted in red.

|  | **bio1** | **bio2** | **bio3** | **bio4** | **bio5** | **bio6** | **bio7** | **bio8** | **bio9** | **bio10** | **bio11** | **bio12** | **bio13** | **bio14** | **bio15** | **bio16** | **bio17** | **bio18** |
| --- | --- | --- | --- | --- | --- | --- | --- | --- | --- | --- | --- | --- | --- | --- | --- | --- | --- | --- |
| **bio2** | -0.33 |  |  |  |  |  |  |  |  |  |  |  |  |  |  |  |  |  |
| **bio3** | 0.55 | -0.01 |  |  |  |  |  |  |  |  |  |  |  |  |  |  |  |  |
| **bio4** | -0.68 | 0.39 | -0.86 |  |  |  |  |  |  |  |  |  |  |  |  |  |  |  |
| **bio5** | 0.88 | -0.07 | 0.22 | -0.27 |  |  |  |  |  |  |  |  |  |  |  |  |  |  |
| **bio6** | 0.96 | -0.46 | 0.67 | -0.84 | 0.72 |  |  |  |  |  |  |  |  |  |  |  |  |  |
| **bio7** | -0.65 | 0.59 | -0.76 | 0.97 | -0.21 | -0.83 |  |  |  |  |  |  |  |  |  |  |  |  |
| **bio8** | 0.79 | -0.30 | 0.20 | -0.28 | 0.81 | 0.67 | -0.29 |  |  |  |  |  |  |  |  |  |  |  |
| **bio9** | 0.93 | -0.25 | 0.66 | -0.78 | 0.75 | 0.94 | -0.73 | 0.53 |  |  |  |  |  |  |  |  |  |  |
| **bio10** | 0.92 | -0.22 | 0.24 | -0.33 | 0.98 | 0.78 | -0.31 | 0.86 | 0.77 |  |  |  |  |  |  |  |  |  |
| **bio11** | 0.96 | -0.38 | 0.70 | -0.85 | 0.73 | 0.99 | -0.81 | 0.67 | 0.95 | 0.78 |  |  |  |  |  |  |  |  |
| **bio12** | 0.55 | -0.68 | 0.43 | -0.63 | 0.25 | 0.65 | -0.71 | 0.40 | 0.50 | 0.36 | 0.62 |  |  |  |  |  |  |  |
| **bio13** | 0.54 | -0.53 | 0.44 | -0.59 | 0.28 | 0.61 | -0.63 | 0.40 | 0.49 | 0.37 | 0.60 | 0.93 |  |  |  |  |  |  |
| **bio14** | 0.20 | -0.66 | -0.16 | -0.15 | 0.08 | 0.27 | -0.31 | 0.17 | 0.15 | 0.18 | 0.20 | 0.46 | 0.25 |  |  |  |  |  |
| **bio15** | 0.06 | 0.46 | 0.21 | -0.04 | 0.12 | 0.00 | 0.10 | 0.09 | 0.06 | 0.06 | 0.06 | -0.12 | 0.13 | -0.57 |  |  |  |  |
| **bio16** | 0.54 | -0.56 | 0.44 | -0.61 | 0.28 | 0.62 | -0.65 | 0.40 | 0.50 | 0.37 | 0.61 | 0.96 | 0.99 | 0.28 | 0.09 |  |  |  |
| **bio17** | 0.26 | -0.69 | -0.07 | -0.24 | 0.11 | 0.34 | -0.40 | 0.20 | 0.22 | 0.21 | 0.28 | 0.54 | 0.33 | 0.98 | -0.56 | 0.36 |  |  |
| **bio18** | 0.26 | -0.59 | 0.15 | -0.35 | -0.01 | 0.33 | -0.47 | 0.27 | 0.17 | 0.12 | 0.30 | 0.78 | 0.68 | 0.44 | -0.17 | 0.72 | 0.49 |  |
| **bio19** | 0.20 | -0.32 | 0.18 | -0.23 | 0.10 | 0.26 | -0.29 | 0.04 | 0.25 | 0.14 | 0.23 | 0.38 | 0.38 | 0.37 | -0.20 | 0.35 | 0.40 | 0.12 |

bio1, annual mean temperature; bio2, mean diurnal range; bio3, isothermality; bio4, temperature seasonality; bio5, max temperature of warmest month; bio6, min temperature of coldest month; bio7, temperature annual range; bio8, mean temperature of wettest quarter; bio9, mean temperature of driest quarter; bio10, mean temperature of warmest quarter; bio11, mean temperature of coldest quarter; bio12, annual precipitation; bio13, precipitation of wettest month; bio14, precipitation of driest month; bio15, precipitation of seasonality; bio16, precipitation of wettest quarter; bio17, precipitation of driest quarter; bio18, precipitation of warmest quarter; bio19, precipitation of coldest quarter.

**Table S2** Comparison between GCMs obtained by CCSM4 and MIROC-ESM simulation and paleoclimate which were obtained by sporopollen, calcium carbonate and ice cores in the Tibetan Plateau

| Code | Locality | Latitude  longitude | Estimated  chronology (kyr BP) | Tann by  CCSM4 (MIROC-ESM) | Tann by reference | Tmax by  CCSM4  (MIROC-ESM) | Tmax by reference | Tmin by CCSM4 (MIROC-ESM) | Tmin by refere-nce | Pann by CCSM4 (MIROC-ESM) | Pann by refere-nce | Reference* |
| --- | --- | --- | --- | --- | --- | --- | --- | --- | --- | --- | --- | --- |
| **Mid-Holocene (6 kyr BP)** | | | | | | | | | | | | |
| 1 | Zoige, Sichuan Province | 33.454N  102.640E | 7.94-6.42 | **1.0**  (**0.6**) | 0.72 | 19.2  (18.1) | 10.17 | -21.3  (-22.4) | -10.37 | **624**  (**661**) | 650.34 | Guo *et al*., 2012 |
| 2 | Hekuo, Xundian, Yunnan Province | 25.833N  103.500E | 8-4 | **13.7**  (**13.3**) | 14.4 | - | No data | - | No data | **1013**  (**1065**) | 1045 | Li *et al.*,  2013 |
| 3 | Gaodian, Hongya, Sichuan Province | 20.583N  103.267E | 11.9-5.0 | **13.9**  (**13.0**) | 12.9 | - | No data | - | No data | 1327  (1484) | 1680 | Shi *et al*.,  2012 |
| 4 | Baxiu&Mozh-gongka, Tibet | 29.827N  92.544E | 6.4 | -2.9  (-3.6) | 3.5 | 12.2  (12.4) | 15.1 | -22.2  (-23.7) | -18.7 | **336**  (**376**) | 394 | Tang *et al*.,  1999 |
| 5 | Bazijiantang, Yunnan Province | 29.816N  99.705E | 6.6-4.9 | 0.9  (0.1) | 6.6 | - | No data | - | No data | **654**  (**668**) | 670 | Shi *et al.*,  2011 |
| 6 | Xiahexi, Baish-ui, Shaanxi Province | 35.140N  109.690E | 5.05-4.87 | **12.7**  (**12.8**) | 12.9 | - | No data | - | No data | 532  (563) | 758.4 | Sun *et al.*,  2014 |
| 7 | Lop Nur, Xinjiang Province | 43.792N  87.624E | 6 | 5.9  (6.8) | 10.2 | 32.6  (35.0) | 27.5 | -20.4  (-19.4) | -16.7 | **232**  (**201**) | 227 | Hao *et al.*, 2012 |
| 8 | Shangri-la, Yunnan Province | 27.817N99.700E | 5.82 | 6.1  (5.7) | 8.2 | - | No data | - | No data | **878**  (**917**) | 830 | Shi, 2012 |
| 9 | Dazhongdianbazi, Yunnan Province | 27.817N  99.705E | 6.6-4.9 | **6.2**  (**5.8**) | 6.6 | - | No data | - | No data | **881**  (**921**) | 950 | Shi *et al*.,  2011 |
| **LGM (21 kyr BP)** | | | | | | | | | | | | |
| 10 | Gaodian, Hongya, Sichuan Province | 20.583N  103.267E | 23.7-21.1 | **9.7**  (11.3) | 9.7 | - | No data | - | No data | 1243  (998) | 1390 | Shi *et al*.,  2012 |
| 11 | Kaiyou, Duyun, Guizhou Province | 26.067N  107.267E | 25.9-15.7 | 12.4  (**14.9**) | 15.3 | - | No data | - | No data | **1080**  (**1067**) | 1089.3 | Li *et al.*,  2013 |
| 12 | Shibazhai, Lijiang, Yunnan Province | 27.500N  99.500E | 49.9-20.3 | **6.8**  (8.2) | 5.6 | 18.2  (22.3) | 26.5 | -7.9  (-6.7) | -19.4 | **858**  (900) | 849.8 | Zhang *et al.*, 2012 |
| 13 | Baxiu&Mozhgongka, Tibet | 29.827N  92.544E | 21-16 | **-7.1**  (-8.7) | -7.5 | **7.9**  (**9.5**) | 8.1 | **-26.9**  (-28.4) | -26.7 | **244**  (198) | 250 | Tang *et al*.,  1999 |
| 14 | Gaodian, Hongya, Sichuan Province | 20.583N  103.267E | 21 | **9.7**  (11.3) | 9.8 | - | No data | - | No data | **1243**  (998) | 1310 | Shi, 2012 |
| **Paleoclimate data for LIG by Otto-Bliesner *et al.*, 2006** | | | | | | | | | | | | |
| 15 | Wugong, Xianyang, Shaanxi Province | 34.262N  108.196E | 120-100 | **13.8** | 15 | - | No data | - | No data | 168 | 800 | Liu, 1989 |

Compared with paleoclimates obtained by sporopollen, calcium carbonate and ice cores, the red font meant error was larger than the black font in GCMs obtained by CCSM and MIROC simulation results. Bold numbers represent temperature difference and rainfall within 1.5 °C, within 100mm, respectively. Tann (annual mean temperature); Tmax (max temperature of warmest month); Tmin (minimum temperature of the coldest month); Pann (annual precipitation). The unit of temperature is °C. The unit of precipitation is mm.

*All references of this table are shown in the supplementary data.

**Table S3** Analysis of phylogeography data available for *A. faxoniana* and *A. recurvata*. (sample size (n), genetic diversity (Pi))

| Species | Site code | Latitude  longitude | Altitude (m) | n | Haplotypes (individuals) | Haplotype diversity, *h* | Nucleotide diversity, Pi |
| --- | --- | --- | --- | --- | --- | --- | --- |
| *A. fargesii* var. *faxoniana* | JJS | 30.867N  102.670E | 3574 | 20 | h1(2)h2(15)h3(1)h4(1)h5(1) | 0.442 | 0.0007 |
|  | HSX | 32.011N  103.029E | 3270 | 20 | h1(17)h6(1)h7(1)h8(1) | 0.284 | 0.0002 |
|  | MXM | 31.665N  103.936E | 3479 | 20 | h1(11)h9(1)h10(2)h11(1)h12(1)h13(4) | 0.442 | 0.0003 |
|  | GGS | 33.045N  103.725E | 3442 | 20 | h1(16)h6(1)h11(1)h14(1)h15(1) | 0.368 | 0.0003 |
|  | REJ | 33.667N  103.485E | 3300 | 20 | h1(12)h6(3)h16(1)h17(2)h18(1)h19(1) | 0.632 | 0.0007 |
|  | MBS | 31.710N  102.307E | 3983 | 20 | h1(13)h6(2)h9(1)h11(1)h17(1)h20(1) h21(1) | 0.579 | 0.0006 |
|  | BX | 33.597N  103.158E | 3230 | 20 | h1(12)h6(2)h17(2)h22(2)h23(1)h24(1) | 0.637 | 0.0005 |
|  | MNG | 32.498N  103.626E | 3000 | 20 | h1(18)h20(2) | 0.189 | 0.0001 |
|  | MYL | 31.567N  102.633E | 3300 | 17 | h1(10)h10(2)h17(1)h20(1)h25(1)h26(1)h27(1) | 0.558 | 0.0004 |
| *A. recurvata* | BY | 31.372N  99.885E | 2979 | 14 | H1(13)H2(1) | 0.143 | 0.0102 |
|  | LT | 31.491N | 3001 | 10 | H3(10) | 0.000 | 0.0000 |
|  | XL | 30.982N  100.097E | 3068 | 7 | H2(1)H3(2) H4(3)H5(1) | 0.809 | 0.0816 |
|  | SD | 31.881N  100.760E | 3302 | 11 | H4(1)H6(9)  H7(1) | 0.346 | 0.0364 |
|  | DF | 31.075N  100.999E | 3060 | 3 | H3(3) | 0.000 | 0.0000 |
|  | DB | 31.868N  101.949E | 2826 | 5 | H8(2)H9(3) | 0.600 | 0.0429 |
|  | MEK | 31.906N  102.206E | 3400 | 9 | H6(6)H10(2)H11(1) | 0.557 | 0.0635 |
|  |  |  |  |  |  |  |  |
|  | H_S_ | H_T_ | G_ST_ | N_ST_ | Fu’s Fs | Tajima’s D | Date of the expansion (kyr BP) |
| *A. farges-ii* var. *faxon-iana* | 0.497 ±  0.060 | 0.610 ± 0.087 | 0.195 ± 0.114 | 0.276 ± 0.141 | -4.519  (P < 0.05) | -1.095  (P < 0.05) | 11.542 |
| *A. recur-vata* | 0.350 ± 0.120 | 0.897 ± 0.076 | 0.609 ± 0.119 | 0.633 ± 0.135 | -11.356  (P < 0.001) | -0.106  (P = 0.427) | 8.086 |

Haplotypes correspond to those in Figure S6. n, number of sampled individuals.

**Table S4** Value range for the different climate scenarios in study area.

|  | Present | | Mid-Holocene | | | | LGM | | | | LIG | |
| --- | --- | --- | --- | --- | --- | --- | --- | --- | --- | --- | --- | --- |
|  |  |  | CCSM | | MIROC | | CCSM | | MIROC | |  |  |
| Climate | Value range | | Value range | | Value range | | Value range | | Value range | | Value range | |
| Tann | 27.5 | -19.4 | 25.8 | -20.6 | 25.4 | -20.7 | 23.7 | -19.6 | 28.5 | -22.2 | 25.6 | -22.6 |
| Tmax | 43.4 | -8.0 | 41.5 | -8.4 | 40.1 | -8.6 | 38.5 | -6.4 | 50.6 | -3.3 | 48.6 | -5.0 |
| Tmin | 15.1 | -36.1 | 13.3 | -38.1 | 13.3 | -38.4 | 10.8 | -39.3 | 11.0 | -40.6 | 11.4 | -47.9 |
| Pann | 11401 | 17 | 12124 | 17 | 13876 | 13 | 9931 | 8 | 12518 | 6 | 11488 | 30 |

Tann bio1, annual mean temperature; bio5, max temperature of warmest month; bio6, min temperature of coldest month; bio12, annual precipitation.
